# Transcriptional responses are oriented towards different components of the rearing environment in two *Drosophila* sibling species

**DOI:** 10.1186/s12864-022-08745-9

**Published:** 2022-07-16

**Authors:** D. De Panis, H. Dopazo, E. Bongcam-Rudloff, A. Conesa, E. Hasson

**Affiliations:** 1 Instituto de Ecología, Genética y Evolución de Buenos Aires, Consejo Nacional de Investigaciones Científicas y Técnicas, Ciudad Autónoma de Buenos Aires, Argentina; 2 Departamento de Ecología, Genética y Evolución, Facultad de Ciencias Exactas y Naturales, Universidad de Buenos Aires, Ciudad Autónoma de Buenos Aires, Argentina; 3 SLU-Global Bioinformatics Centre, Department of Animal Breeding and Genetics, Swedish University of Agricultural Sciences, Uppsala, Sweden; 4 Microbiology and Cell Science Department, University of Florida, Gainesville, Florida USA

**Keywords:** Comparative transcriptomics, Adaptation, Plasticity, *Drosophila*, Mescaline

## Abstract

**Background:**

The chance to compare patterns of differential gene expression in related ecologically distinct species can be particularly fruitful to investigate the genetics of adaptation and phenotypic plasticity. In this regard, a powerful technique such as RNA-Seq applied to ecologically amenable taxa allows to address issues that are not possible in classic model species. Here, we study gene expression profiles and larval performance of the cactophilic siblings *Drosophila buzzatii* and *D. koepferae* reared in media that approximate natural conditions and evaluate both chemical and nutritional components of the diet. These closely related species are complementary in terms of host-plant use since the primary host of one is the secondary of the other. *D. koepferae* is mainly a columnar cactus dweller while *D. buzzatii* prefers *Opuntia* hosts.

**Results:**

Our comparative study shows that *D. buzzatii* and *D. koepferae* have different transcriptional strategies to face the challenges posed by their natural resources. The former has greater transcriptional plasticity, and its response is mainly modulated by alkaloids of its secondary host, while the latter has a more canalized genetic response, and its transcriptional plasticity is associated with the cactus species.

**Conclusions:**

Our study unveils a complex pleiotropic genetic landscape in both species, with functional links that relate detox responses and redox mechanisms with developmental and neurobiological processes. These results contribute to deepen our understanding of the role of host plant shifts and natural stress driving ecological specialization.

**Supplementary Information:**

The online version contains supplementary material available at 10.1186/s12864-022-08745-9.

## Background

For many insects, host plants constitute the fundamental environmental factor, decisively affecting stages of their life cycles [1]. Since plants can synthesize a large variety of secondary metabolites as defences to herbivores, insect populations undergoing a host-plant shift can be exposed to chemical environments that may be dramatically different from the ancestral host [2]. Therefore, shifting to new hosts may impose novel selective pressures driving phenotypic and genetic change, promoting divergence and, eventually, speciation as consequences of adaptation to the new environment [3, 4]. Thus, acquisition of new hosts may lead to ecological specialization producing biological diversity, enabling the coexistence of multiple species [5]. Such specialization may be a consequence of adaptive changes in mechanisms that allow an insect to face the potential chemical stress inherent to a host-plant shift and define the range of resource exploitation [6]. In this context, the transcriptional plasticity that enables phenotypic accommodation to the novel host may facilitate the posterior adaptation [7] since plastic traits can be canalized through the fixation of genetic changes that modulate environmental responsiveness to optimize performance [8].

Genomic and post-genomic approaches provide unprecedented opportunities to understand host shifts and the consequent potential to survive environmental changes [9]. Studying transcriptomic responses to alternative host plants helps to identify genetic elements deployed and unravel the complexity of the gene network involved in each case [10]. A detailed understanding of gene expression differences between related species adapted to alternative hosts can shed light on the role of plasticity in adaptation to novel environments, as well as the evolution of host plant specialization and species divergence [11]. Nowadays, the maturity of high-throughput sequencing platforms allows unprecedented resolution in these kinds of studies in organisms exploiting particularly interesting natural environments, opening new dimensions to ecology and evolutionary biology research [12, 13].

In the genus *Drosophila*, species of the *repleta* group are particularly tractable for eco-evolutionary studies [14]. Most of these flies can use decaying cactus tissues as breeding and feeding substrates and be classified in two main groups, one breeding chiefly on cacti of the genus *Opuntia* and the other mainly in columnar cacti of the subfamily Cactoideae [15]. Moreover, regarding evolution of host plant use in these flies, the evidence points to *Opuntia* cacti as the ancestral state [16]. Switches to columnar cacti of greater chemical complexity occurred independently on several occasions along the evolutionary history of the group. Some columnar specialists lost the ability to use *Opuntia* as alternative hosts, while others can use both types of plants. This highlights the potential of ecological specialization during the radiation of cactophilic flies in American deserts.

The cactophilic sibling species *Drosophila buzzatii* and *Drosophila koepferae* are members of the *buzzatii* cluster (buzzatii complex, repleta group), a guild of seven closely-related species endemic to semiarid zones of South America. A recent study pointed out that *D. buzzatii* and *D. koepferae* diverged 2–3 million years ago [17]. The former is the only species of the cluster that became sub-cosmopolitan following the expansion of its main hosts across the world, *Opuntia* cacti [18]. *D. koepferae* inhabits the eastern slopes of pre-Andean mountain ranges in northwestern Argentina and southern Bolivia, where columnar cacti of the genera *Trichocereus*, *Cereus* and *Neoraimondia* are its primary hosts [15]. These species have partially overlapping distributions across most of *D. koepferae* geographic range, where the primary host of one species is the secondary host of the other. In some areas, the most common hosts of *D. buzzatii* and *D. koepferae* are *Opuntia sulphurea* and *Trichocereus terscheckii*, respectively [15]. Studies addressing the nutritional composition of *O. sulphurea* and *T. terscheckii* showed that the former has slightly more free sugars and total fats [19, 20]. However, the most distinctive compositional difference between these cacti is that *T. terscheckii* has ten times more alkaloids than *O. sulphurea*. Moreover, the characterization of alkaloid fractions isolated from each species revealed phenethylamine derivatives, mainly mescaline (3,4,5-trimethoxyphenethylamine) and trichocerein (N,N-dimethylmescaline) in *T. terscheckii*, and proline derivatives of unreported toxicity in *O. sulphurea* [21].

Field and laboratory studies evaluated the influence that developing in alternative cactus hosts has on several fitness-related traits in both species. The general conclusion is that these hosts impose differential selective pressures on both species suggesting that performance traits have probably evolved as adaptations to exploit resources with different ecological (spatial and temporal predictability) and compositional (nutritional quality and allelochemical) properties (reviewed in [15]). Moreover, *T. terscheckii* alkaloids were shown to be less harmful to *D. koepferae* than to *D. buzzatii* [22, 23], suggesting its prominent role in host-plant specificity. More recently, we showed that the switch from *O. sulphurea* to *T. terscheckii* triggers in *D. buzzatii* a wide transcriptomic response modulated mainly by the presence of alkaloids [21]. Such transcriptional plasticity involved detox and stress-response genes, but also genes related to redox and developmental processes. Based on this background, the study of transcriptional strategies deployed in response to alternative breeding environments may help to understand the adaptations that evolved in each species in response to the use of their host plants in nature.

Intra and interspecific transcriptomic comparisons between closely-related species adapted to novel host plants provide an excellent opportunity to study processes driving divergence [11]. Inferring the adaptive value of plastic responses to new environments in the complex transcriptional landscapes shown by whole insects in their hosts remains challenging [10]. To tackle this problem, looking at the functional links of differentially expressed genes (DEGs) in ecologically amenable species proves fundamental [9].

In this paper, we report the results of a comparative transcriptomic study aimed to understand the genetic responses deployed by the recently diverged species *D. buzzatii* and *D. koepferae* to breeding in alternative host cacti under different chemical (added alkaloids or native concentration) and nutritional conditions (addition of protein extract) (see Table 2 in Material and Methods section for details of the experimental design). To this end, we assessed expression profiles in both species under various rearing conditions. We also performed an exploratory analysis of expression profiles between species within each treatment. We hypothesise that *D. buzzatii* deploys more plastic transcriptomic responses than its sibling *D. koepferae* upon changes in rearing conditions. These expectations are based on the fact that *D. koepferae* is a phylogenetically derived species [24] that experienced a shift to more chemically hostile host plants, like columnar cacti, and therefore should show canalized gene expression patterns in comparison to *D. buzzatii*, a dweller of the benignant ancestral host plants. In addition, we report the first de novo genome assembly for *D. koepferae* to use as reference.

Our survey revealed quite different transcriptional responses between these sibling species, as gene expression profiles were mainly modulated by alkaloids in *D. buzzatii*, while alternative host cacti were the main factor driving differential gene expression in *D. koepferae*. These results point to divergent genetic outcomes resulting from ecological specialization. Finally, we discuss the functional relationships of transcriptional responses in the ecological context of both species.

## Results

### Fitness-related trait variation across treatments

**Fig. 1:**
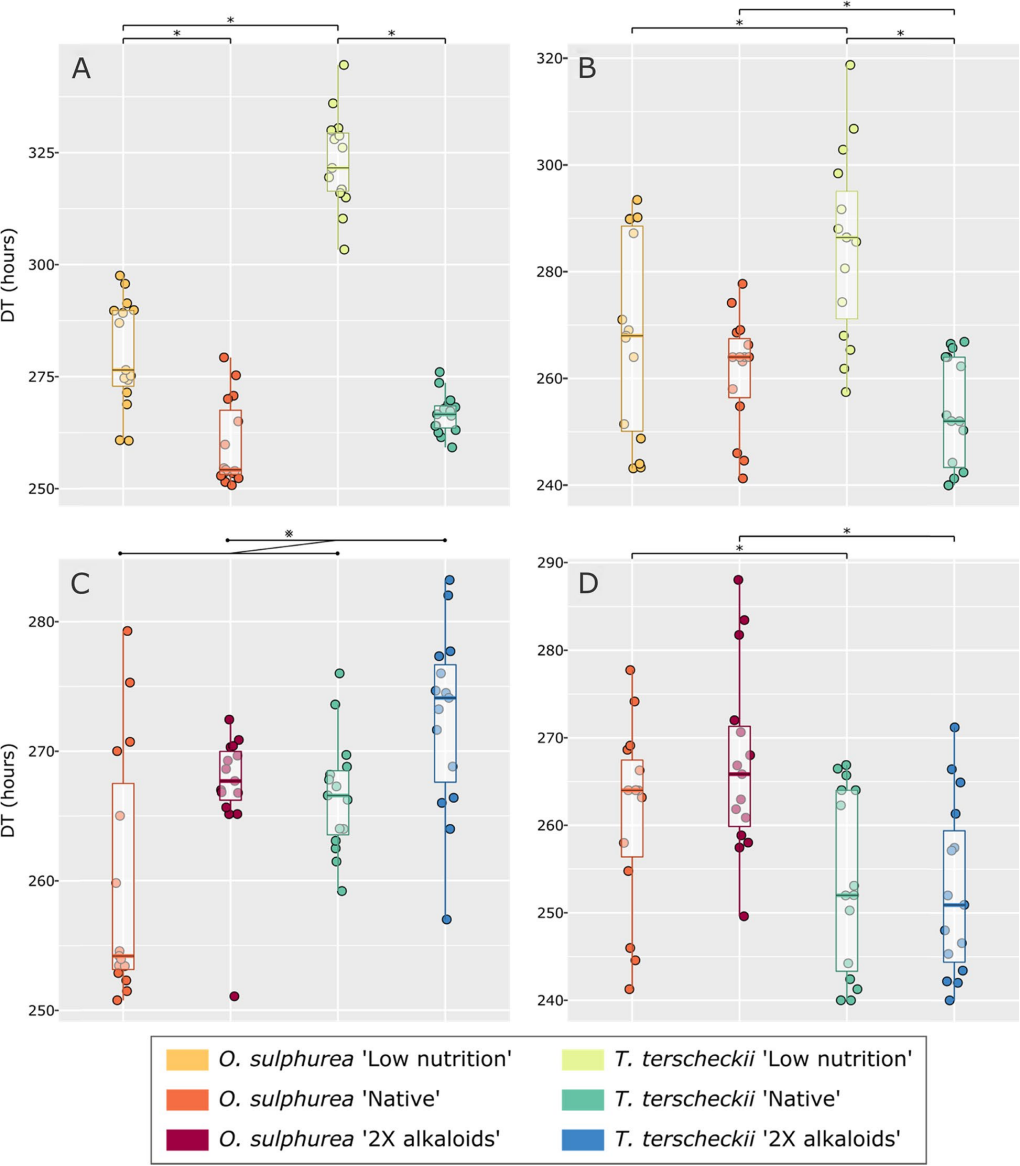
Developmental time (DT) expressed in hours; each dot represents one of the 5 vials (experimental units) assessed per genotype (biological replicates). **A-B** Analysis of the nutritional component related to the protein fraction evaluated in the experimental design, in *D. buzzatii* (A) and *D. koepferae* (B). **C-D** Analysis of the chemical component related to phenethylamine alkaloids fraction evaluated in the experimental design, in *D. buzzatii* (C) and *D. koepferae*. (D). Statistical significance between treatments is *p* < 0.05 (*), and a trend is shown in panel C between the two assessed conditions with 0.05 < *p* < 0.10 (⨳)

Analyzes of Developmental Time (DT) as a proxy of larval performance in the same semi-natural conditions aimed to assess gene expression showed that *D. buzzatii* reared in *T. terscheckii* 'Low nutrition' took significantly more time to reach adulthood than in the remaining treatments. Moreover, we found that this species significantly extended DT when raised in 'Low nutrition' treatments (Fig. 1.A). Finally, we observed a trend (*p* < 0.095) towards longer DT in treatments with higher alkaloids concentration (Fig. 1.C). In turn, *D. koepferae* reared in *O. sulphurea* took significantly longer time to reach adulthood than in *T. terscheckii*, both in 'Native' and '2X alkaloids' conditions (Fig. 1.D). However, this trend was reversed in flies raised in 'Low nutrition' condition, since DT was significantly longer in *T. terscheckii* than in *O. sulphurea* (Fig. 1.B).

### Genome assembly, annotation and mapping

Assuming a genome size similar to *D. buzzatii*, we obtained a total 263X coverage for *D. koepferae* after quality control and filtering of genomic reads (Supporting Information, C.3). Using the assembly protocol schematized in Fig. S1, we produced an assembly of size and quality similar to the available for *D. buzzatii* (Table 1). We annotated 14,134 protein-coding genes in *D. koepferae* genome, an amount in the range of the available in *D. buzzatii* (13,567 protein-coding genes, Supporting Information E.1). Next, we functionally annotated both genomes using the same strategy. The percentages of fully annotated genes were 84% for *D. koepferae* (of the remaining, up to 10% with annotation hints) and 80% for *D. buzzatii* (of the remaining, up to 13% with annotation hints) (Fig. S2).

**Table 1:** Scaffolds length distribution and classic contiguity indicators to evaluate the genome assembly of *D. koepferae* (genotype F) and its functional annotation. The BUSCO parameters are used to estimate the assembly completeness based on the expected gene content. All values were also calculated for the reference genome of *D. buzzatii* for comparative purposes

Assembly	*D. koepferae* (genotype F)	*D. buzzatii* (reference)
Total length (bp)	169,200,914	160,803,536
Total scaffolds	1,373	722
Scaffolds ≥ 5000 bp	772	588
Scaffolds ≥ 50,000 bp	347	256
Longest scaffold (bp)	5,773,295	16,306,990
N50 (bp)	807,095	1,380,942
L50	63	30
Total Ns (bp)	5,747,328	14,818,525
% GC	38.0	38.5
BUSCO Completeness (%)	98.0	97.0
BUSCO Fragmented (%)	1.3	1.7
BUSCO Missing (%)	0.2	0.4
Fully annotated genes	11,804	10,983

RNA-Seq reads were mapped to the reference genomes after quality control and filtering. Mapping efficiency was within the 3rd quantile (> 40–60%) across all genotypes of both species using *D. mojavensis* as reference in exploratory INTER-specific analyzes and did not differ between species (F_1,30_ = 2,841; p = 0.102). In turn, mapping efficiency was within the 5th quantile (> 80–100%) for the respective genotypes using *D. buzzatii* or *D. koepferae* in INTRA-specific analyzes (Supporting Information, F.1).

### INTER-specific gene expression analyzes

A total of 2972 genes were differentially expressed considering all INTER-specific pairwise comparisons within treatments (*i.e.* between fly species for each treatment). Both in exploratory two-replicate as three-replicate comparisons, we found more genes overexpressed in *D. buzzatii* than in *D. koepferae*, while the largest expression asymmetry was observed in the treatment *O. sulphurea* '2X alkaloids' (Fig. S3).

**Fig. 2:**
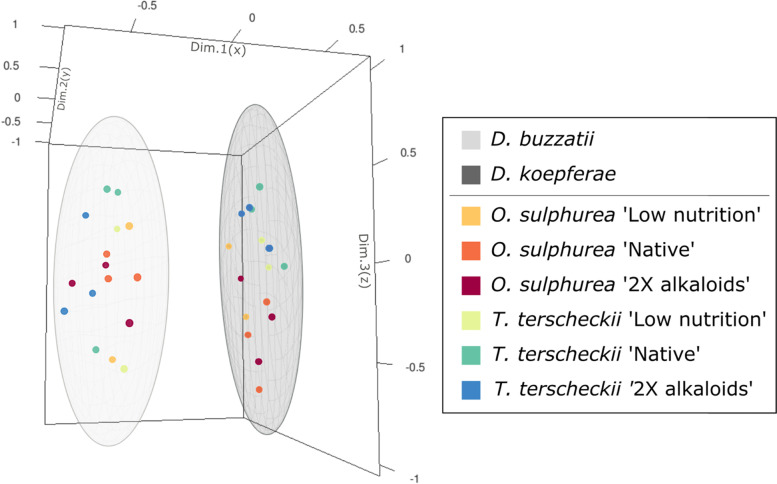
Multidimensional Scaling of expression values of all DEGs between *D. buzzatii* and *D. koepferae* across treatments in exploratory INTER-specific analyzes. Ellipses of concentration with a confidence of 95% are represented around the dataset of each species. Dots represent biological replicates

To visualize general patterns of gene expression in comparisons between species, expression values of each one of the DEGs within each treatment were reduced to 3 dimensions by means of MDS. With this methodology, we observed that the two fly species occupy clearly separated expression spaces within the set of evaluated treatments (Fig. 2). Moreover, it can be seen that treatments are arranged according to cactus species in *D. koepferae* (Fig. 2). Similar results were obtained using an analytical approach based on principal component analysis (Fig. S4).

### Comparative characterization of transcriptomic responses

Functional enrichment analyzes of INTER-specific comparisons revealed marked differences between the sets of DEGs in each species exposed to the same treatments. For exploratory 'Low nutrition' treatments, we obtained only a few GO enriched terms and therefore a clear picture of the DEGs functionality cannot be ascertained. On the contrary, the remaining treatments provided a clearer idea about the processes in which the sets of DEGs are involved (Table S1). Genes with higher comparative expression in *D. buzzatii* raised in *O. sulphurea* 'Native' were mainly related to aromatic amino acid metabolism, protein degradation, cuticle development, and pigmentation. In contrast, genes overexpressed in *D. koepferae* were mainly enriched in cellular respiration and energy metabolism, synthesis of amino acids, peptides and nucleic acids. In *O. sulphurea* '2X alkaloids', the set of genes comparatively more expressed in *D. buzzatii* was strongly enriched in functions related to oxidation–reduction and detoxification processes (though some terms observed in *O. sulphurea* 'Native' were also detected). Instead, the functional profiling of genes overexpressed in *D. koepferae* in *O. sulphurea* '2X alkaloids' was mainly related to muscle development, besides some other terms also observed in *O. sulphurea* 'Native'. Interestingly, the functional enrichment of DEGs with higher comparative expression in *D. buzzatii* raised in *T. terscheckii* 'Native' showed a strong redox-detox component as well as other previously obtained terms. In turn, signal of functions related to muscle development was observed in genes overexpressed in *D. koepferae* in *T. terscheckii* 'Native'. Finally, genes comparatively more expressed in *D. buzzatii* raised in *T. terscheckii* '2X alkaloids' exhibited a strong signal of redox-detox related terms as well as peptide synthesis. In contrast, the genes overexpressed in *D. koepferae* involved terms related to development.

Heatmaps with DEGs belonging to six enzymatic groups related to xenobiotic metabolism were built to explore the relative differential expression analysis between species (Fig. S5). The general pattern emerging throughout all comparisons from most groups of genes showed that the numbers of overexpressed genes were greater in *D. buzzatii* than in *D. koepferae*, except for Glycosyltransferases that exhibited roughly the same amount of DEGs in both species, and GSTs that only showed overexpression in *D. buzzatii*. Also, we constructed another heatmap including the 100 most-variable DEGs. Among these, 17 belong to the enzymatic groups mentioned above and 17 were genes with an unknown associated function (Fig. S6).

### INTRA-specific differential gene expression

Transcriptional responses in each species were evaluated by means of pairwise comparisons between cacti for each condition (*i.e*., *O. sulphurea* vs *T. terscheckii* for 'Low nutrition', 'Native' and '2X alkaloids'), and between conditions for each cactus (*i.e.*, 'Low nutrition' vs 'Native', and 'Native' vs '2X alkaloids', for *O. sulphurea* and *T. terscheckii*).

The three-replicate contrasts revealed higher levels of differential gene expression in the comparisons between treatments that differed in alkaloids concentration in *D. buzzatii* (Table S2), whereas differential expression was higher in comparisons between cacti in *D. koepferae* (Table S3).

**Fig. 3:**
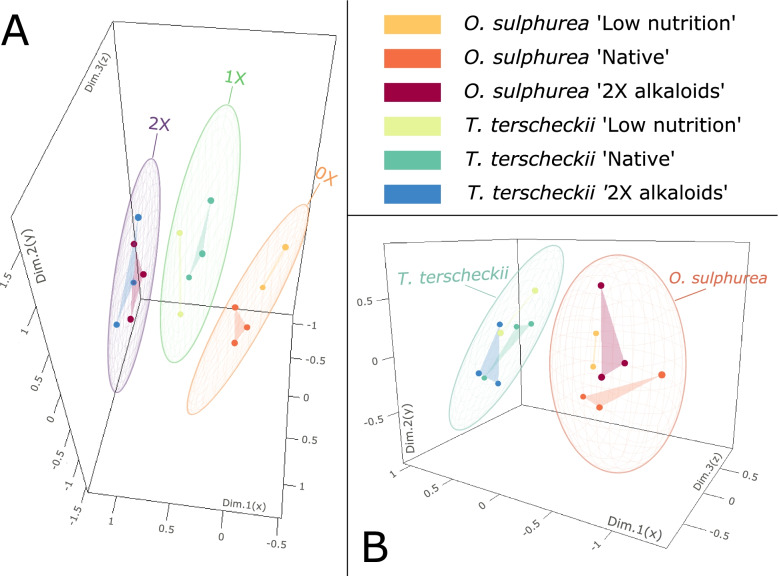
Multidimensional Scaling based on gene expression values using all DEGs in *D. buzzatii* (**A**) and *D. koepferae* (**B**) across treatments in INTRA-specific analyzes. Biological replicates represented by dots are linked for each treatment by a line or a triangular plane (depending on the number of replicates) for better visualization. **A** Ellipses of concentration with a confidence of 95% are represented around data for a given alkaloid concentration (0X, 1X or 2X). **B** Ellipses of concentration with a confidence of 95% are represented around data for a given cactus species (*O. sulphurea* or *T. terscheckii*)

In the MDS obtained for each species using all DEGs in all pairwise comparisons, treatments are clearly separated in the gene expression space according to alkaloids concentration (*i.e.*, 0X, 1X or 2X) in *D. buzzatii*, and, as glimpsed in the exploratory INTER-specific analysis, depending on the cactus host in *D. koepferae* (Fig. 3).

### Characterization of each species transcriptional profiles

We observed enrichment of GO terms related to energy metabolism and development in *D. buzzatii* raised in *O. sulphurea* 'Native', and mainly to detox and redox processes in treatments with higher alkaloids concentration (Tables S4, S6). In *D. koepferae* we detected enrichment in terms associated with energy metabolism, protein synthesis and development in *O. sulphurea* and regulation of pigmentation and neurobiological processes in *T. terscheckii* across 'Native' conditions and '2X alkaloids' conditions. In turn, in the exploratory comparison between cacti across 'Low nutrition' conditions we observed detox terms in both cacti, and energy metabolism terms only in *T. terscheckii* (Tables S5,S7). In addition, for the main ontology terms of each species taking into account all DEGs, we observed a greater component of catalytic functions (*e.g.*, redox) in *D. buzzatii* and development and regulation components besides response to chemical stimuli and stress in *D. koepferae* (Fig. S7).

To refine the characterization of transcriptional responses we constructed heatmaps involving different sets of DEGs. The trends described in the previous paragraph can be observed in such heatmaps. First, we selected the subset of DEGs belonging to any of the six enzymatic groups related to xenobiotic metabolism to build heatmaps. For *D. buzzatii*, it included 3 SLC transporters, 1 Carboxylesterase, 43 Oxidoreductases, 6 GSTs, and 4 Glycosyltransferases (no ABC transporters were found differentially expressed considering the employed cutoff). The heatmap for *D. koepferae* included 15 SLC transporters, 4 Carboxylesterases, 54 Oxidoreductases, 3 GSTs, 6 Glycosyltransferases, and 3 ABC transporters (Fig. S8-S9). Second, we constructed heatmaps including the 100 most-variable DEGs in each species, to extend the analysis and explore not necessarily-expected genes (Fig. S10).

## Discussion

During adaptation to new hosts, genetic changes cause the evolution of multiple traits. However, a phenotype is not entirely controlled by the genotype since environmental conditions can also contribute to variation. Moreover, the genetic background affects the resulting phenotype, suggesting that compensatory mechanisms can buffer the environmental effects. Thus, the resulting phenotype in a particular environment may be a trade-off between phenotypic plasticity and canalization [25]. The model system defined by the sibling species *D. buzzatii* and *D. koepferae* in their natural breeding resources is particularly attractive for comparative studies aimed to understand the role of host plant shifts and the stress associated with specialization to novel environments. The results of the analyzes of larval performance in the semi-natural media are in line with previous field and laboratory reports (reviewed in [15]), validating our experimental design. This suggests that each species’ performance was differentially affected by the hosts' chemical and nutritional conditions in which they were reared, the latter being an aspect not previously investigated in this model.

The transcriptional plasticity observed in *D. buzzatii*, particularly in alkaloid-rich media, offers a plausible explanation of its ability to exploit a wide host-range. In contrast, *D. koepferae*'s transcriptome is more canalized towards this challenge, a likely outcome of specialization to chemically complex columnar cacti that constitute the core of its diet. Assuming *D. buzzatii* as representative of the ancestral state of host-plant utilization, *D. koepferae* represents the derived state entailing adaptation to chemically-complex hosts. In this scenario, the host shift to columnar cacti experienced by *D. koepferae* seems to have involved the assimilation of variants of detox-related genes. Such divergent patterns of host use are dramatically reflected in our study by the distinct gene expression spaces occupied by both species. Moreover, our comparative study shows differential transcriptional plasticity between *D. buzzatii* and *D. koepferae*. The first exhibited plastic responses to varying alkaloids concentrations, while the latter to alternative host plants.

INTER-specific exploratory analyzes revealed a large volume of DEGs overexpressed in both species, suggesting that each one has transcriptional programs involving different genetic elements in the same rearing conditions. These comparisons also showed that *D. koepferae* overexpressed more genes in *T. terscheckii* than in *O. sulphurea*, while *D. buzzatii* in nutritionally supplemented media with higher alkaloids concentration. The higher amount of DEGs in some of the INTRA-specific comparisons involving an alkaloids-free treatment in *D. buzzatii* suggests that in media with an equivalent protein component, transcriptomic responses are largely conditioned by the presence of alkaloids. Indeed, numbers of DEGs in comparisons involving *O. sulphurea* 'Native' were several times lower than in comparisons between hosts with added alkaloids and *T. terscheckii* 'Native' vs '2X alkaloids', pointing that *D. buzzatii* is more sensitive to alkaloids presence rather than any other difference between cacti. Moreover, the number of DEGs in the comparison between *T. terscheckii* treatments differing in alkaloid concentration suggests a subtle dose-sensitive response. *D. koepferae*'s transcriptional responses were clearly different. The amount of DEGs in the comparison between hosts with added alkaloids and supplemented with dead-yeast extract indicates a higher sensitivity towards the type of cactus, neither related to the chemical nor the protein component.

The general picture emerging from these results points to species-specific transcriptional strategies, with *D. buzzatii* responding to the chemical challenge imposed by alkaloids with a classic redox-detox imprint, and *D. koepferae* to the type of cactus involving elements linked to development. By exploring functional relationships and the background of DEGs, we found evidence that points to a wide-transcriptomic response linking diverse processes.

Though we are interested in genome-wide gene expression patterns, it is relevant to point out that we employed lines homozygous for different chromosomal arrangements as biological replicates in each species. Therefore, these lines may potentially have different gene combinations co-expressed due to physical proximity (or even behave like ‘supergenes’) in non-collinear chromosomes. However, to investigate this possible effect and properly evaluate co-expression of DEGs, we may need not only chromosome-level genomes assemblies for gene mapping with higher-resolution in both species but also additional lines homozygous for the same arrangements.

In the subsequent sections, we delve into the main processes shown to be overrepresented to give context and possible clues about previously observed phenotypes.

### Detox component of transcriptomic responses

In the exploratory INTER-specific comparisons, more genes related to xenobiotic metabolism were overexpressed in *D. buzzatii* than in *D. koepferae* (Fig. S5), which may be indicative of a comparatively greater modulation of the detox genetic response in the former.

INTRA-specific analyzes showed some DEGs with the same annotation in both *D. buzzatii* and *D. koepferae*. Many are Phase I genes related to xenobiotic metabolism, like Cytochrome P450s, and Phase II genes, like Glutathione S-transferases and Glycosyltransferases (Fig. S8-S9). CYP enzymes are fundamental elements in insect adaptation due to their role in metabolism and detoxification [26]. This function is shared especially with the insect-specific Delta and Epsilon classes of GSTs [27] and with UGTs that play a role in detoxification and resistance by regulating bioactivity and solubility of different compounds [28]. These genes were similarly overexpressed in media containing alkaloids in both species.

Other detox-related genes exhibited species-specific patterns. In *D. buzzatii*, we found overexpression in alkaloids-containing treatments of CYP and GST genes, and the gene related to pesticide resistance *CHKov1* [29]. Though we expected to detect these kinds of genes differentially expressed under chemical stress conditions because they are associated with responses to alternative hosts or xenobiotics like insecticides [30], our study highlights the connection between these genes and the phenethylamines-rich *T. terscheckii* (Fig. S8). Also, many genes related to redox processes were overexpressed at higher alkaloids concentration, posing a direct functional link with detox processes, like *Fmo-2* known to be recruited during insect adaptation to plants that accumulate toxic alkaloids [31]. Additionally, we found other genes related to cellular redox-state homeostasis or counterparts of detoxification [32], like peroxidases, overexpressed in alkaloids-containing treatments. Interestingly, some genes related to detox-redox were also overexpressed in treatments a priori considered less chemically stressful (*e.g.*, *O. sulphurea* 'Native').

In *D. koepferae*, we detected a few genes directly related to detoxification processes (Fig. S9). Among these, *Ugt301D1* was overexpressed in *T. terscheckii* treatments, while *GstD10* and the esterase *α-Est7* in *O. sulphurea* treatments. Likewise, we found other genes linked to detox processes through the response to oxidative stress and redox homeostasis. In this group, some genes like the *Jafrac* thioredoxin peroxidases were overexpressed in alkaloid-containing media, while others were overexpressed in *O. sulphurea*.

These results illustrate that *D. buzzatii* and *D. koepferae* can regulate coordinated transcriptional responses to xenobiotics as reported in other insects [33]. Moreover, DEGs related to detoxification in alkaloids-rich treatments may be considered as candidate genes involved in adaptation to natural resources containing mescaline-like alkaloids. Furthermore, genes sharing similar expression patterns may be part of detox responses common to both species. Nevertheless, species-specific expression involving alternative sets of genes was triggered in alkaloid-rich media. Lastly, reactive oxygen species (ROS) are produced by CYP enzymes under stress conditions induced by xenobiotic substances, and excessive ROS can modify the cellular redox state, leading to oxidative stress and protein damage [32]. Thus, some detox-related redox genes seem to be involved in physiological countermeasures to cope with that kind of disruption, suggesting a detox-derived oxidative-stress scenario [21].

### Host change and genes associated with development

Several DEGs related to xenobiotic metabolism are also associated with developmental processes. Perhaps the most prominent case can be found among CYP enzymes since many also participate in pathways related to insect hormones that regulate growth and development [26]. Oxidoreductases like *Aldh*, involved in juvenile hormone (JH) biosynthesis, and *Jheh2*, involved in JH hydrolysis, were overexpressed in alkaloids-containing treatments in both species. Insect hormones are not only instrumental in orchestrating development but are also involved in stress-response, behaviour and diapause [34].

Other genes related to insect hormones like *Fdx2,* involved in regulation of ecdysteroids synthesis, were overexpressed in alkaloids-containing media, and *Eo*, which encodes an ecdysteroid-inactivating oxidase, underexpressed in '2X alkaloids' treatments in *D. buzzatii*. Likewise, in *D. koepferae* we found an ecdysone-inducible ABC transporter gene related to circadian rhythm regulation, capable of modulating the ecdysone response and linked to phenotypic abnormalities [35], underexpressed in *O. sulphurea* 'Native' and '2X alkaloids' treatments. Similarly, ecdysone-inducible *Eip* genes, involved in response to oxidative stress and transcription regulation, and *Imp* genes, related to imaginal disks morphogenesis, were underexpressed in nutritionally-supplemented *O. sulphurea*. Moreover, in *D. koepferae* hormone-receptor genes related to developmental progression were underexpressed in nutritionally-supplemented *O. sulphurea* treatments. The differential expression of these hormone-related genes, tightly linked to the insect's growth program, is likely to translate into developmental delay [36] and could reasonably be related to our DT results. In this vein, the oxidase *Loxl1* whose inhibition is related to developmental delay [37], was overexpressed in *D. koepferae* larvae raised in *T. terscheckii* treatments, consistent with its longer DT in *O. sulphurea*.

Many of the 100 most-variable DEGs in *D. koepferae* are related to body structures development (Fig. S10). Particularly interesting are those that regulate wing development since they may account for phenotypic syndromes, like abnormalities in wing morphology and venation reported in both *D. buzzatii* and *D. koepferae* reared in alternative hosts and alkaloids-containing media [38]. Moreover, along with DEGs linked to flight behaviour like *Gpdh*, genes involved in wing development offer a plausible explanation for courtship-song plasticity induced by rearing cacti [39].

### Differential expression of cuticle and pigmentation genes

Detection of cuticle-related genes is not surprising since it is a key component in insect-environment interactions [40]. Many of the 100 most-variable DEGs detected in INTRA-specific analysis in both species are related to cuticle development (Fig. S10). In *D. buzzatii*, a few of these genes seemed to be modulated by alkaloids-containing media, and by cactus hosts in *D. koepferae*. Interestingly, differential expression of genes related to chitin metabolism and cuticle may provide signatures of delayed development [36], adding more genetic background to account for our results of DT variation. Besides, cuticle genes are related to increased protection against surface exposure to toxic compounds [33], minimizing xenobiotics entry by thickening the cuticle and stabilizing gut structure [41], providing a link with the detox response.

In addition, several genes instrumental in the biotransformation of precursors into pigment molecules to be later incorporated in the cuticle were differentially expressed in both species. In *D. koepferae*, the genes *pale*, *Ddc* and *yellow* involved in the production of DOPA, dopamine and melanin were underexpressed in nutritionally-supplemented *O. sulphurea*. Dopamine, a monoamine neurotransmitter that notably belongs to the phenethylamines like *T. terscheckii* alkaloids, is a precursor of melanin, the insects' central pigment [42]. In *D. buzzatii*, *Ddc* and *yellow* were overexpressed in *O. sulphurea* 'Native'. Moreover, other genes like serine proteinase inhibitors related to melanization, linked to dopamine biosynthesis or production of precursors involved in pigmentation and cuticle hardening like *black* and *ebony* [43], were differentially expressed in *D. koepferae* and *D. buzzatii* in distinct conditions.

Some of these pigmentation genes are known to exhibit a high degree of transcriptional plasticity modulated by the environment in *Drosophila*, in agreement with our results. Thus, variation in wing development as found in previous studies in *D. buzzatii* and *D. koepferae* in its hosts may impact melanization since precursors diffusion through wing venation is related to pigmentation patterns [44]. Therefore, our results offer a likely genetic mechanism to explain abnormalities in wing melanization observed in *D. buzzatii* reared in alkaloids-containing media [45]. Further, since different stressing conditions can modulate expression of some of these genes [46] and *T. terscheckii* is a stressful environment for *D. buzzatii* larvae [23], a breeding environment effect on some of these genes is plausible, either directly or indirectly.

### Cactus, alkaloids and neuro-related processes

Pigmentation is intrinsically related to fly's neurobiology. Dopamine is a widely conserved neurotransmitter, responsible for the control of voluntary movement, arousal, sleep, male courtship behaviour and learning in *Drosophila* [47]. Furthermore, genes essential in melanin pathways like *pale*, *Ddc*, *ebony* and *black*, also regulate dopamine synthesis and availability in the insect brain [48], affecting neural functions.

Many alkaloids found in columnar cacti are known to affect neurotransmission [1]. *T. terscheckii*'s mescaline and trichocereine are psychoactive substituted phenethylamines. Interestingly, these alkaloids are biosynthesized from dopamine, which is also present in lesser quantities in *T. terscheckii* [49]. This provides another potential point of chemical modulation by the rearing medium since ingested dopamine can affect fly's nervous system [48]. Additionally, some DEGs like *Aldh* that degrades dopamine metabolites as well as other toxic species [50] link detox-redox with neuro functions, while others could play a role in mescaline inactivation by mediating dopamine clearance in the synaptic cleft [48]. Furthermore, the fact that dopamine self-oxidizes generating ROS [51], points to a relationship between genes with protective roles in oxidative damage and dopaminergic neuron function [52]. Dopamine is also a neuromodulator and neurohormone that can impact insect development [53], providing another glimpse of the complexity of the interactions between processes affected by the rearing environment in *Drosophila*.

### Transcriptional profiles and nutritional challenge

Many DEGs mentioned so far are related to oxidative-stress responses, pointing towards a scenario of apparent mitochondrial high-demand. These redox-related responses also share intermediaries and mechanisms with processes of energy metabolism. Additionally, differential expression of other mitochondria-related genes may be interpreted as nutritional stress responses [36].

Several DEGs were related to nutritional metabolism, either linked to catabolism and energy generation along with the regeneration of both redox potential (e.g. NAD +) and citric acid cycle intermediates, or to processes like biosynthesis and storage. Interestingly, some of these genes were overexpressed in alkaloids-containing treatments in both *D. buzzatii* and *D. koepferae*. Such expression patterns may indicate that the xenobiotic detox-redox response can also have consequences in nutrition-related processes, an example being the already mentioned gene *Jafrac* in *D. koepferae*, which is also linked to starvation response.

Nutritionally relevant genes related to proteolysis, that were differentially expressed in *Opuntia sulphurea* treatments in both *D. buzzatii* and *D. koepferae*, may be involved in adaptation to alternative host plants that could contain different sets of protease inhibitors [41]. Though there is evidence of protease inhibitors in some *Opuntia* species [54], whether the host cacti used in our experiments produce these inhibitors is unknown.

All in all, the genes associated with nutritional challenges and other stress conditions [36], such as those posed by alternative host-cacti, suggest common elements and pathways. For instance, SLCs that play a role in dietary absorption in addition to xenobiotics excretion were differentially expressed across treatments particularly in *D. koepferae*.

### Concluding remarks and perspectives

Transcriptomic responses to natural breeding environments are nothing less than wide and complex, making genetic landscapes hard to interpret. Our study explores the biological responses underlying patterns of differential gene expression, shedding light on how different physiological processes may be interlinked. Such multigenic response involved in adaptation to challenging conditions spreads genome-wide over interconnected pathways in a way not fully understood and with outcomes hard to predict [55]. For instance, differential expression of redox genes involved in antioxidant response can impact downstream phenomena like lifespan [56]. *Jheh2* is an example, since it is associated with pesticide resistance and oxidative-stress response in addition to regulating JH and, therefore, having multiple effects on development and physiology. Our study suggests a transcriptomic and physiological cross-talk between the detox-redox response to xenobiotics and developmental programs. Another example of genes affecting diverse traits can be found among those involved in determining body colour. These highly pleiotropic pigmentation genes affect multiple processes and its interplays, like vision or mating behaviour [57].

Altogether, our study contributes new insights into the biological functions associated with particular conditions and the genes involved. Moreover, it helps to deepen our understanding of the genetic and ecological factors implied in host plant shifts and the role of transcriptional plasticity in adaptation and specialization in a group of recently diverged *Drosophila*.

## Methods

### Samples collection and alkaloids extraction

*D. buzzatii* and *D. koepferae* isofemale lines used in this study were obtained during a summer collection trip to sites of north-western Argentina where both species coexist. Flies were recovered (in different proportions) from rotting pieces of both *O. sulphurea* and *T. terscheckii*. Posteriorly, the progenies of wild inseminated females were used to establish inbred lines fixed for the most frequent second chromosomal arrangements by sib-mating for eight generations. At the end of this procedure, we obtained three lines homozygous for arrangements *standard*, *j* and *jz*^*3*^ (genotypes A, B and C, respectively) of *D. buzzatii*, and three *D. koepferae* lines homozygous for arrangements *l*^*9*^*m*^*9*^, *l*^*9*^*m*^*9*^*n*^*9*^ and *l*^*9*^*m*^*9*^ (genotypes D, E and F, respectively). Genotype F was also homozygous for inversions *k*^*2*^, *m* and *w* in chromosomes 3, 4 and 5, respectively. We considered each isofemale line as a particular genotype containing different genetic backgrounds and used them as biological replicates. Chromosomal arrangements that went fixed as a result of inbreeding allowed us to control the maintenance of the genotype before the experiments described below (Supporting Information, A.1).

Fresh pieces of *O. sulphurea* and *T. terscheckii* were collected in northwestern Argentina where native cacti are abundant and *D. buzzatii* and *D. koepferae* are sympatric. Plant material was identified [58] and stored frozen since collection (Supporting Information, A.2). Finally, an alkaloid fraction enriched in phenethylamines was obtained from fresh tissues of *T. terscheckii* as described in [21].

### Experimental design and treatments

The treatments used in the present study were conceived as good approximations to evaluate the effects of cactus hosts and phenethylamine alkaloids on gene expression profiles. We avoided adding fractions over which we cannot exert clear control like the microflora associated with decomposing cacti. Instead, we exploratorily investigated a nutritional component through the addition of a supplement composed of dead yeast to mimic the protein fraction contributed by cactophilic yeasts to flies' natural diet. In this vein, treatments without the nutritional supplement can be thought of as a scenario of early colonization of potential breeding sites, where the necrotic cactus pocket is young and microorganisms' load low. To this end, batches of 50 individuals of the different genotypes of each species (biological replicates) were exposed to six rearing media (treatments) from the first to the third instar larval stages (Supporting Information, B.1).

Thus, two treatments aimed to assess the host plant effect consisted in rearing larvae in semi-natural media prepared with fresh tissues of *O. sulphurea* or *T. terscheckii*, supplemented with a dead-yeast extract (hereafter, 'Native' condition). The dead-yeast extract was added to emulate the protein contribution made by cactophilic yeasts involved in plant's decaying process in flies' natural diet, avoiding a possible nutritional deficiency due to lack of this macronutrient [59]. Two other treatments aimed to evaluate the effects of *T. terscheckii* alkaloids involved the same semi-natural media described above plus the addition of the proper amount of the alkaloids extract to reach a final concentration of two-fold the native in fresh *T. terscheckii* (hereafter, '2X alkaloids' condition). Finally, we included two treatments to exploratorily investigate the effect of the manipulated nutritional component, which consisted of media elaborated with fresh tissues of *O. sulphurea* or *T. terscheckii*, without dead-yeast extract (hereafter, 'Low nutrition' condition). All treatments are summarized in Table 2.

**Table 2:** Experimental design. Summary of the treatment. Since *O. sulphurea* contains no phenethylamine alkaloids, its basal concentration is 0X, whereas for *T. terscheckii* the basal concentration is 1X

	Treatments					
Cactus	*O. sulphurea*			*T. terscheckii*		
Condition	**Low** **nutrition**	**Native**	**2X** **alkaloids**	**Low** **nutrition**	**Native**	**2X** **alkaloids**
Cactus host of (primary / secondary)	*D. buzzatii* / *D. koepferae*	*D. buzzatii* / *D. koepferae*	*D. buzzatii* / *D. koepferae*	*D. koepferae* / *D. buzzatii*	*D. koepferae* / *D. buzzatii*	*D. koepferae* / *D. buzzatii*
Dead-yeast extract added	No	Yes	Yes	No	Yes	Yes
Alkaloids added	No	No	Yes	No	No	Yes
Total alkaloids	0X	0X	2X	1X	1X	2X

For each treatment, groups of 50 first-instar larvae were transferred to vials with the corresponding rearing medium (Supporting Information, B.2). From a total of 15 vials for each combination of treatment, species and genotype, 10 were randomly set apart to obtain batches of third-instar larvae for RNA-Seq and the remaining 5 were assigned for DT measurement. All vials were incubated at 25 ± 1 °C, 12:12 h light:dark photoperiod and 60 ± 10% relative humidity. Vials for RNA-Seq were incubated until larvae reached the third-instar stage (diagnosed with the beginning of the wandering phase) and were gently removed, rapidly washed thrice in sterile PBS, snap-frozen with liquid nitrogen and finally stored at -80 °C until RNA extraction. Vials for DT were incubated until adults’ emergence.

### Developmental time measurement

DT is widely used as an indicator of the degree of adaptation of an organism to a particular environment [60]. We measured DT as the time elapsed from the transfer of first-instar larvae to vials until adult emergence.

A generalized linear mixed-effects model (GLMM) was fitted to test DT differences across treatments, using vials as experimental units (mean DT per vial as dependent variable), and “Cactus” (*O. sulphurea*, *T. terscheckii*) and “Condition” (Native, 2X alkaloids, Low nutrition) as fixed crossed-factors. Each fly species dataset was analyzed separately due to significant interactions among factors (Supporting information, G). Specific random terms used in the final model for each species included biological replicates (genotypes) and were selected using the Akaike Information Criterion (AIC) and significant in Likelihood Ratio. Finally, to evaluate the effects of the nutritional and chemical components on DT, the previous data sets were split. Thus, for each fly species, the data were fitted to two new models: one including 'Low nutrition' and 'Native' conditions and another 'Native' and '2X alkaloids' conditions (Supporting information, G). All statistical analyzes and respective visualizations were carried out in R 3.6 [61].

### Genome sequencing, assembly and annotation

Because there is not genomic data available, we sequenced the genome of *D. koepferae* to use as mapping reference for RNA-Seq reads in the respective gene expression analysis. To this end, high-molecular-weight DNA was extracted from adult flies of genotype F using a purification protocol optimized for *D. melanogaster* (Gentra® Puregene® Cell Kit) coupled with a standard Phenol:Chloroform extraction for final clean-up. DNA was sequenced in an Illumina HiSeq 2000 platform using paired-end libraries (2 × 101 bp, insert size = 400 bp, coverage > 100X), and a mate-pair library (2 × 50 bp, insert size = 5 Kbp, coverage > 25X). Another sample was sequenced in a Pacific Biosciences (PacBio) RS II platform employing 2 SMRT cells (P6/C4), using a library suitable to obtain an average read size of 10 Kbp and coverage > 5X (Supporting Information, C.2).

After reads were quality controlled and processed accordingly, we worked on a de novo genome assembly protocol that integrates data obtained with different technologies using available software (Supporting information, C.3-D.1). The protocol is depicted in Fig. S1. The assembled genome of *D. koepferae* was structurally-annotated using GenSAS v5.0 [62]. The identification of protein-coding genes and other features along the scaffolds was based on evidence from multiple sources, and posteriorly integrated into a single final annotation (Supporting information, D.2-E.1). Functional annotation of protein-coding genes of *D. koepferae* and *D. buzzatii* genomes was performed with Blast2GO 5 [63]. The high-quality functional annotation available for *D. melanogaster* [64] was used to assign identity to all the genes to facilitate the understanding of the transcriptional landscape and the comparison of results (Supporting information, E.2).

### Transcriptome sequencing and gene expression analysis

Total RNA was extracted from third-instar larvae samples using a combined TRIzol®/RNeasy® protocol optimized for Drosophila [65]. RNA concentration, quality and integrity were checked before libraries preparation (Supporting Information, C.3). Thirty-two paired-end libraries (2 × 101 bp, insert size = 150 bp) were sequenced in an Illumina HiSeq 2000 platform. RNA samples of genotypes C (*D. buzzatii*) and F (*D. koepferae*) reared in 'Low nutrition' treatments were not sequenced because of logistical reasons.

After quality control and processing, we mapped RNA-Seq reads to the *D. buzzatii* or *D. koepferae* genome to estimate gene expression of each genotype within each species (INTRA-specific) under the evaluated conditions using the program STAR v2.6 [66]. To obtain unbiased between-species exploratory comparisons (INTER-specific), we also mapped RNA-Seq reads of each genotype to the *D. mojavensis* reference genome v1.04 [67], a species of the repleta group that is phylogenetically equidistant from *D. buzzatii* and *D. koepferae* [24]. Thus, though a lower mapping efficiency may be expected, it would be the same for both species. To evaluate this, we checked for mapping bias in the dataset using Qualimap v2.2.1 [68] (Supporting Information, F.1). The quantification of gene expression in each case was calculated with StringTie v1.3.5 [69]. Differential gene expression analyzes were performed with the NOISeqBIO method implemented in the R package NOISeq v2.18 [70] using TMM-normalized raw reads counts. The statistical strategy of this package considers both differences in mean expression and orders of magnitude of differences to measure the change in gene expression between conditions and therefore identify DEGs. NOISeqBIO is optimized for the use of at least two biological replicates per condition and has a sensitivity (proportion of true DE calls out of the total number of DEGs) of 90% and 95% and FDR slightly above 5% and lower than 5% with two and three replicates per condition, respectively (see Supporting information F.2 for further insight on the method). However, it is worth mentioning that all comparisons involving 'Low nutrition' treatments (two replicates each) were considered as exploratory. In all cases, a False Discovery Rate (FDR) < 0.01 was used as stringent cut-off value.

### Analysis of gene expression patterns

We used the Multi-Dimensional Scaling (MDS) function in the SMACOF R package [71] to visualise transcriptional similarities across treatments and biological replicates. Functional enrichment analyzes of Gene Ontology (GO) terms and biological pathways were performed on the DEG sets across the evaluated comparisons to characterize the transcriptional profiles (Supporting information, F.2). The identity assigned to each DEG was based on the respective *D. melanogaster* homologue. Since searching without regard to particular candidate genes should reveal a wider spectrum of genes that would have otherwise been ignored [10], our analysis was not limited to specific genes. However, based on previous studies that showed a strong presence of DEGs related to detoxification processes, we looked for DEGs belonging to classes and families involved in the four phases of general xenobiotic transport and metabolism [72, 73]. These correspond mainly to six enzymatic groups: Solute carriers (SLC) transporters involved in Phase 0; Carboxylesterases and Oxidoreductases like Cytochrome P450s (CYP) and Alcohol dehydrogenases (ADH) in Phase I; Glutathione S-transferases (GST) and Glycosyltransferases like UDP-glucuronosyltransferases (GT) in Phase II; and ATP-binding cassette (ABC) transporters in Phase III. Finally, to study possible trends emerging from expression patterns within treatments and conditions, we visualized groups of DEGs using the functions hclust (ward.D2 clustering method) and heatmap from the R packages GRAPHICS and STATS, respectively.

## Supplementary Information

**Additional file 1.** Supporting document: Detailed methods, procedures, and protocols.

**Additional file 2.** Supporting tables: **TableS1-S7**.

**Additional file 3.** Supporting figures: **Fig.S1-S10**.

## Data Availability

The datasets generated and analysed during the current study have been deposited in NCBI with BioProject ID: PRJNA731932 (https://www.ncbi.nlm.nih.gov/bioproject/731932), PRJNA858943 (https://www.ncbi.nlm.nih.gov/bioproject/858943) and PRJNA314520 (https://www.ncbi.nlm.nih.gov/bioproject/314520).

## References

[1] Schoonhoven LM, van Loon JJA, Dicke M (2005). Insect-Plant Biology.

[2] Matzkin LM (2012). Population transcriptomics of cactus host shifts in Drosophila mojavensis. Mol Ecol.

[3] Specialization, Speciation, and Radiation: The Evolutionary Biology of Herbivorous Insects. 1st edition. University of California Press; 2008. https://www.jstor.org/stable/10.1525/j.ctt1pnq3k.

[4] Nosil P. Ecological Speciation. Oxford University Press; 2012. 10.1093/acprof:osobl/9780199587100.001.0001

[5] Forister ML, Dyer LA, Singer MS, Stireman JO, Lill JT (2012). Revisiting the evolution of ecological specialization, with emphasis on insect–plant interactions. Ecology.

[6] Gloss AD, Vassão DG, Hailey AL, Nelson Dittrich AC, Schramm K, Reichelt M (2014). Evolution in an Ancient Detoxification Pathway Is Coupled with a Transition to Herbivory in the Drosophilidae. Mol Biol Evol.

[7] Malka O, Santos-Garcia D, Feldmesser E, Sharon E, Krause-Sakate R, Delatte H (2018). Species-complex diversification and host-plant associations in Bemisia tabaci: A plant-defence, detoxification perspective revealed by RNA-Seq analyses. Mol Ecol.

[8] Schneider RF, Meyer A (2017). How plasticity, genetic assimilation and cryptic genetic variation may contribute to adaptive radiations. Mol Ecol.

[9] Markow TA (2019). Host use and host shifts in Drosophila. Current Opinion in Insect Science.

[10] Etges WJ (2019). Evolutionary genomics of host plant adaptation: insights from Drosophila. Current Opinion in Insect Science.

[11] Birnbaum SSL, Abbot P (2020). Gene Expression and Diet Breadth in Plant-Feeding Insects: Summarizing Trends. Trends Ecol Evol.

[12] Hoang K, Matzkin LM, Bono JM (2015). Transcriptional variation associated with cactus host plant adaptation in Drosophila mettleri populations. Mol Ecol.

[13] Orsucci M, Audiot P, Nidelet S, Dorkeld F, Pommier A, Vabre M (2018). Transcriptomic response of female adult moths to host and non-host plants in two closely related species. BMC Evol Biol.

[14] Markow TA, O’Grady PM (2007). Drosophila Biology in the Genomic Age. Genetics.

[15] Hasson E, De Panis D, Hurtado J, Mensch J (2019). Host Plant Adaptation in Cactophilic Species of the Drosophila buzzatii Cluster: Fitness and Transcriptomics. J Hered.

[16] Oliveira DCSG, Almeida FC, O’Grady PM, Armella MA, DeSalle R, Etges WJ (2012). Monophyly, divergence times, and evolution of host plant use inferred from a revised phylogeny of the Drosophila repleta species group. Mol Phylogenet Evol.

[17] Moreyra NN, Mensch J, Hurtado J, Almeida F, Laprida C, Hasson E (2019). What does mitogenomics tell us about the evolutionary history of the Drosophila buzzatii cluster (repleta group)?. PLoS ONE.

[18] Manfrin MH, Sene FM (2006). Cactophilic Drosophila in South America: A Model for Evolutionary Studies. Genetica.

[19] Padro J, Soto IM (2013). Exploration of the nutritional profile of Trichocereus terscheckii (Parmentier) Britton & Rose stems. J Professional Assoc Cactus Develop.

[20] Carreira VP, Padró J, Koch NM, Fontanarrosa P, Alonso I, Soto IM (2014). Nutritional composition of Opuntia sulphurea G. Don cladodes Haseltonia.

[21] De Panis DN, Padró J, Furió-Tarí P, Tarazona S, Carmona PSM, Soto IM (2016). Transcriptome modulation during host shift is driven by secondary metabolites in desert Drosophila. Mol Ecol.

[22] Corio C, Soto IM, Carreira V, Padró J, Betti MIL, Hasson E (2013). An alkaloid fraction extracted from the cactus Trichocereus terscheckii affects fitness in the cactophilic fly Drosophila buzzatii (Diptera: Drosophilidae). Biol J Linn Soc.

[23] Soto IM, Carreira VP, Corio C, Padró J, Soto EM, Hasson E (2014). Differences in Tolerance to Host Cactus Alkaloids in Drosophila koepferae and D. buzzatii. PLoS One.

[24] Hurtado J, Almeida F, Revale S, Hasson E (2019). Revised phylogenetic relationships within the Drosophila buzzatii species cluster (Diptera: Drosophilidae: Drosophila repleta group) using genomic data. Arthropod Syst Phylogeny..

[25] Gibert J-M, Peronnet F, Schlötterer C (2007). Phenotypic Plasticity in Drosophila Pigmentation Caused by Temperature Sensitivity of a Chromatin Regulator Network. PLoS Genet.

[26] Feyereisen R (1999). Insect P450 Enzymes. Annu Rev Entomol.

[27] Li L, Gao X, Lan M, Yuan Y, Guo Z, Tang P (2019). De novo transcriptome analysis and identification of genes associated with immunity, detoxification and energy metabolism from the fat body of the tephritid gall fly Procecidochares utilis. PLOS ONE.

[28] Huang F-F, Chai C-L, Zhang Z, Liu Z-H, Dai F-Y, Lu C (2008). The UDP-glucosyltransferase multigene family in Bombyx mori. BMC Genomics.

[29] Aminetzach YT, Macpherson JM, Petrov DA (2005). Pesticide Resistance via Transposition-Mediated Adaptive Gene Truncation in Drosophila. Science.

[30] Després L, David J-P, Gallet C (2007). The evolutionary ecology of insect resistance to plant chemicals. Trends Ecol Evol.

[31] Sehlmeyer S, Wang L, Langel D, Heckel DG, Mohagheghi H, Petschenka G (2010). Flavin-Dependent Monooxygenases as a Detoxification Mechanism in Insects: New Insights from the Arctiids (Lepidoptera). PLoS ONE.

[32] He L, He T, Farrar S, Ji L, Liu T, Ma X (2017). Antioxidants Maintain Cellular Redox Homeostasis by Elimination of Reactive Oxygen Species. CPB.

[33] Misra JR, Horner MA, Lam G, Thummel CS (2011). Transcriptional regulation of xenobiotic detoxification in Drosophila. Genes Dev.

[34] Dubrovsky EB (2005). Hormonal cross talk in insect development. Trends Endocrinol Metab.

[35] Hock T, Cottrill T, Keegan J, Garza D (2000). The E23 early gene of Drosophila encodes an ecdysone-inducible ATP-binding cassette transporter capable of repressing ecdysone-mediated gene activation. PNAS.

[36] Fernández-Ayala DJM, Chen S, Kemppainen E, O’Dell KMC, Jacobs HT (2010). Gene Expression in a Drosophila Model of Mitochondrial Disease. PLoS ONE.

[37] Molnar J, Ujfaludi Z, Fong SFT, Bollinger JA, Waro G, Fogelgren B (2005). Drosophila Lysyl Oxidases Dmloxl-1 and Dmloxl-2 Are Differentially Expressed and the Active DmLOXL-1 Influences Gene Expression and Development. J Biol Chem.

[38] Padró J, Carreira V, Corio C, Hasson E, Soto IM (2014). Host alkaloids differentially affect developmental stability and wing vein canalization in cactophilic Drosophila buzzatii. J Evol Biol.

[39] Iglesias PP, Soto EM, Soto IM, Colines B, Hasson E (2018). The influence of developmental environment on courtship song in cactophilic Drosophila. J Evol Biol.

[40] Vogel H, Musser RO, Celorio‐Mancera M de la P. Transcriptome Responses in Herbivorous Insects Towards Host Plant and Toxin Feeding. In: Annual Plant Reviews. John Wiley & Sons, Ltd; 2014. p. 197–233. 10.1002/9781118829783.ch6.

[41] de la Celorio-Mancera MP, Wheat CW, Vogel H, Söderlind L, Janz N, Nylin S (2013). Mechanisms of macroevolution: polyphagous plasticity in butterfly larvae revealed by RNA-Seq. Molecular Ecol.

[42] Wittkopp PJ, Beldade P (2009). Development and evolution of insect pigmentation: Genetic mechanisms and the potential consequences of pleiotropy. Semin Cell Dev Biol.

[43] Rawls JM (2006). Analysis of Pyrimidine Catabolism in Drosophila melanogaster Using Epistatic Interactions With Mutations of Pyrimidine Biosynthesis and β-Alanine Metabolism. Genetics.

[44] Massey JH, Wittkopp PJ. Chapter Two - The Genetic Basis of Pigmentation Differences Within and Between Drosophila Species. In: Orgogozo V, editor. Current Topics in Developmental Biology. Academic Press; 2016. p. 27–61. 10.1016/bs.ctdb.2016.03.004. 10.1016/bs.ctdb.2016.03.004 PMC5002358 27282023

[45] Mongiardino-Koch N, Hasson E (2012). Differences in wing melanization and pigmentation pattern in Drosophila buzzatii (Diptera: Drosophilidae) under chemical stress. Revista de la Sociedad Entomológica Argentina.

[46] Rauschenbach IY, Karpova EK, Adonyeva NV, Andreenkova OV, Faddeeva NV, Burdina EV (2014). Disruption of insulin signalling affects the neuroendocrine stress reaction in Drosophila females. J Exp Biol.

[47] Cichewicz K, Garren EJ, Adiele C, Aso Y, Wang Z, Wu M (2017). A new brain dopamine-deficient Drosophila and its pharmacological and genetic rescue. Genes Brain Behav.

[48] Yamamoto S, Seto ES (2014). Dopamine Dynamics and Signaling in *Drosophila*: An Overview of Genes Drugs and Behavioral Paradigms. Experimental Animals.

[49] Padro, De Panis, Luisi et al. Ortholog genes from cactophilic Drosophila provide insight into human adaptation to hallucinogenic cacti. 2022. Submitted. 10.1038/s41598-022-17118-x PMC9343604 35915153

[50] Martin CA, Barajas A, Lawless G, Lawal HO, Assani K, Lumintang YP (2014). Synergistic effects on dopamine cell death in a Drosophila model of chronic toxin exposure. Neurotoxicol.

[51] Hanna ME, Bednářová A, Rakshit K, Chaudhuri A, O’Donnell JM, Krishnan N (2015). Perturbations in dopamine synthesis lead to discrete physiological effects and impact oxidative stress response in Drosophila. J Insect Physiol.

[52] Whitworth AJ, Theodore DA, Greene JC, Beneš H, Wes PD, Pallanck LJ (2005). Increased glutathione S-transferase activity rescues dopaminergic neuron loss in a Drosophila model of Parkinson’s disease. PNAS.

[53] Hodgetts RB, O’Keefe SL (2006). DOPA DECARBOXYLASE: A Model Gene-Enzyme System for Studying Development, Behavior, and Systematics. Annu Rev Entomol.

[54] Torres Castillo JA, Varela Martínez K, Blanco-Labra A, Mondragón Jacobo C. PROTEASE INHIBITORS PRESENT IN OPUNTIA SPP. Acta Hortic. 2009;:293–8. 10.17660/ActaHortic.2009.811.39

[55] Seong KM, Coates BS, Sun W, Clark JM, Pittendrigh BR (2017). Changes in Neuronal Signaling and Cell Stress Response Pathways are Associated with a Multigenic Response of Drosophila melanogaster to DDT Selection. Genome Biol Evol.

[56] Orr WC, Radyuk SN, Sohal RS (2013). Involvement of Redox State in the Aging of Drosophila melanogaster. Antioxid Redox Signal.

[57] Massey JH, Akiyama N, Bien T, Dreisewerd K, Wittkopp PJ, Yew JY (2019). Pleiotropic Effects of ebony and tan on Pigmentation and Cuticular Hydrocarbon Composition in Drosophila melanogaster. Front Physiol..

[58] Saint Esteven A, Benedictto M, Garolla FA, Padró J, Soto IM (2021). A survey of cacti richness in a biodiversity hotspot of Western Argentina. Bradleya.

[59] Mongiardino-Koch N, Soto IM, Galvagno M, Hasson E, Iannone L (2015). Biodiversity of cactophilic microorganisms in western Argentina: community structure and species composition in the necroses of two sympatric cactus hosts. Fungal Ecol.

[60] Parsons PA, Stanley SM, Spence GE (1979). Environmental Ethanol at Low Concentrations: Longevity and Development in the Sibling Species Drosophila Melanogaster and D. Simulans Aust J Zool.

[61] R Core Team. R: A language and environment for statistical computing. R Foundation for Statistical Computing. Vienna, Austria; 2019. https://www.R-project.org/.

[62] Humann JL, Lee T, Ficklin S, Main D, Kollmar M (2019). Structural and Functional Annotation of Eukaryotic Genomes with GenSAS. Gene Prediction: Methods and Protocols.

[63] Conesa A, Götz S, García-Gómez JM, Terol J, Talón M, Robles M (2005). Blast2GO: a universal tool for annotation, visualization and analysis in functional genomics research. Bioinformatics.

[64] Thurmond J, Goodman JL, Strelets VB, Attrill H, Gramates LS, Marygold SJ (2019). FlyBase 2.0: the next generation. Nucleic Acids Res.

[65] Bogart K, Andrews J (2006). Extraction of total RNA from Drosophila. Center Genomics Bioinform CGB Tech Rep.

[66] Dobin A, Davis CA, Schlesinger F, Drenkow J, Zaleski C, Jha S (2013). STAR: ultrafast universal RNA-seq aligner. Bioinformatics.

[67] Clark AG, Eisen MB, Smith DR, Bergman CM, Oliver B, Markow TA (2007). Evolution of genes and genomes on the Drosophila phylogeny. Nature.

[68] Okonechnikov K, Conesa A, García-Alcalde F (2016). Qualimap 2: advanced multi-sample quality control for high-throughput sequencing data. Bioinformatics.

[69] Pertea M, Pertea GM, Antonescu CM, Chang T-C, Mendell JT, Salzberg SL (2015). StringTie enables improved reconstruction of a transcriptome from RNA-seq reads. Nat Biotechnol.

[70] Tarazona S, Furió-Tarí P, Turrà D, Pietro AD, Nueda MJ, Ferrer A (2015). Data quality aware analysis of differential expression in RNA-seq with NOISeq R/Bioc package. Nucleic Acids Res.

[71] De Leeuw J, Mair P (2009). Multidimensional Scaling Using Majorization: SMACOF in R. J Stat Softw.

[72] Li X, Schuler MA, Berenbaum MR (2007). Molecular Mechanisms of Metabolic Resistance to Synthetic and Natural Xenobiotics. Annu Rev Entomol.

[73] Döring B, Petzinger E (2014). Phase 0 and phase III transport in various organs: combined concept of phases in xenobiotic transport and metabolism. Drug Metab Rev.

