# Supplementary material for: Transcriptional responses are oriented towards different components of the rearing environment in two Drosophila sibling species
Source: BMC Genomics. 2022 Jul 16;23:515. doi: 10.1186/s12864-022-08745-9 (PMC9288027; doi:10.1186/s12864-022-08745-9)
Supplement: Supplementary file 1 — Additional file 1. Supporting document: Detailed methods, procedures, and protocols. [file 12864_2022_8745_MOESM1_ESM.pdf]

Transcriptional responses are oriented towards different components of the rearing environment in two *Drosophila* sibling species. De Panis et al. 2021.

Supporting Information

INDEX

A.1. Flies collection 02

A.2. Cactus samples 02

B.1. Treatments preparation 03

B.2. Experiment setup 03

C.1. Transcriptomes sequencing 04

C.2. *Drosophila koepferae* genotype F genome sequencing 04

C.3. Reads processing 05

D.1. De novo genome assembly pipeline 06

D.2. De novo genotype F transcriptome assembly 07

E.1. Structural annotation of *D. koepferae* genome 07

E.2. Functional annotation of *D. buzzatii* and *D. koepferae* genomes 08

F.1. RNA-seq reads mapping 11

F.2. Differential gene expression analyzes 15

G. Developmental time 17

H. References 27

A.1. Flies collection

The *D. buzzatii* and *D. koepferae* lines used in this study were obtained by J. Hurtado and E. Hasson during a summer collection trip in February of 2010. The lines were collected in sites of northwestern Argentina where both species coexist and were recovered (in different proportions) from rotting pieces of both *O. sulphurea* and *T. terscheckii*. Posteriorly, the lines were fixed for the most frequent chromosomal arrangements by sib mating (see Ruiz & Wasserman 1993 for a description of the arrangements).

Briefly, the progenies of wild inseminated females of *D. buzzatii* and *D. koepferae* collected in the field were used to establish inbred lines (hereafter, genotypes) monomorphic for the most frequent second chromosome arrangements of each species by sib-mating for eight generations. Crosses in which the progeny had the desired karyotype were selected for further inbreeding. Three genotypes homozygous for the arrangements *standard*, *j* and jz^3 (genotypes A, B and C, respectively) of *D. buzzatii*, and three *D. koepferae* genotypes homozygous for the arrangements l^9m^9, $l^9m^9n^9$ and l^9m^9 (genotypes D, E and F, respectively) were employed in the experiments. Genotype F was also homozygous for inversions k^2, *m* and *w* in chromosomes 3, 4 and 5, respectively. After establishment of the genotypes, flies were maintained in standard laboratory medium under controlled conditions with frequent inspections to verify homozygosity.

A.2. Cactus samples

Fresh and healthy cladodes of *O. sulphurea* and whole arms of *T. terscheckii* were gently removed from the plants using a knife in the field during a summer campaign in 2012 in Valle Fértil reserve, a protected area in western Argentina (San Juan province). The area, adjacent to the Ischigualasto Provincial Park and the Talampaya National Park, comprises 800000 hectares in the ecoregions known as Monte de Sierras y Bolsones (80% of the area) and Chaco Seco (the remaining 20%). It is an area of important biodiversity and endemism (Roig-Juñent et al. 2001), where the flora is identified with the vegetation of the mountains and Prepunean cacti of the genera *Trichocereus*, *Cereus* and *Opuntia*. Fresh cactus collections were carried out in a valley within the reserve (coordinates 30°41'S, 67°29'O, and 1200 meters above sea level), where native cacti are found in high density and *D. buzzatii* and *D. koepferae* are sympatric. Plant material was identified in situ by Saint Esteven et al. (2021). Cactus was frozen *in situ*, kept chilled on transit, and once in the laboratory, tissues were manipulated in an aseptic cabin and stored fractionated (only the chlorenchyma in the case of *T. terscheckii*) at -80 °C. At the time of use, stored cacti were thawed and kept at 8

°C. This procedure aimed to preserve the cactus tissues chemically intact, and at the same time, reduce the presence of any type of microorganisms coming from the field.

Cactus collections were made with the authorization of the corresponding local authorities (file No. 1300-0236-13, Directorate of Conservation and Protected Areas, SAyDS, San Juan province).

B.1. Treatments preparation

For each 'Native' treatment, 5.8 g of one cactus and 0.2 g of yeast extract were added to the respective culture vials (see De Panis *et al*. 2016 for details). '2X alkaloids' treatments were set up by the addition of 2.4 mg of the alkaloid extract in 0.1 mL of ethanol in the vials containing *T. terscheckii*, and 4.8 mg in 0.1 mL of ethanol in *O. sulphurea* vials. The same volume of ethanol (0.1 mL) was added to the vials to which the alkaloid fraction was not added. The vials of 'Low nutrition' treatments contained 6 g of the respective cactus to match the total mass used in the other treatments, plus the addition of the same volume of ethanol as used in treatments without added alkaloids.

B.2. Experiment setup

First-instar larvae of each genotype were obtained by releasing batches of sexually mature flies in egg-collecting chambers (acrylic boxes of 5x10x15 cm) with a Petri dish containing an egg-laying medium (2% agar). Petri dishes were removed after 12 h, inspected for the presence of eggs, and incubated for 24 h at 25 °C to allow hatching and immediate larvae transfer. For each treatment, groups of freshly hatched 50 first-instar larvae were randomly sampled and transferred to vials with the corresponding rearing medium. From the total of 15 vials (replicates) for each combination of treatment, species and genotype, 10 were set apart to obtain batches of third-instar larvae for RNA-Seq (the purpose of this high number of vials is having backup material if problems during RNA extraction or sequencing arose), and the remaining 5 were assigned for developmental time measurement as a proxy of larval performance. Vials were incubated at 25±1 °C, 12:12 h light:dark photoperiod and 60±10 % relative humidity. Replicates for RNA-seq were incubated until larvae reached the third-instar stage. This stage was determined by the beginning of the wandering phase, a period where the larva wanders out of the food, begins wandering and climbs. Under optimum conditions at 25 °C, this is reported to occur approximately 24 hours after the start of the third instar larval stage (Ashburner, 1989). Larvae were gently removed from each vial, rapidly washed thrice in sterile PBS, randomly picked and snap-frozen in groups of five with liquid nitrogen, to finally storing at -80 °C until RNA extraction.

C.1. Transcriptomes sequencing

Total RNA was extracted from a pool of 15 third-instar larvae sampled randomly from 3 replicate-vials (5+5+5, to minimize vial effect) in all combinations of species, genotype and treatment using a combined TRIzol®/RNeasy® protocol optimized for Drosophila (Bogart & Andrews 2006). RNA concentration and quality were initially assessed by measuring A260/280 nm and A260/230 nm absorbance ratios using a NanoDrop® spectrophotometer. RNA integrity was checked in 1% agarose bleach-gel (Aranda et al. 2012) and before library preparation in a 2100 Bioanalyzer system (Agilent Technologies).

All RNA samples were sequenced by the Illumina HiSeq 2000 platform using paired-end libraries (2x101 bp, insert size=150 bp), except genotypes C (*D. buzzatii*) and F (*D. koepferae*) reared in 'Low nutrition' treatments that were not sequenced because of logistical reasons. Both library preparation and sequencing, totalling 32 transcriptomes, were performed at Centre Nacional d'Anàlisi Genòmica (Barcelona, Spain).

C.2. Drosophila koepferae genome sequencing

We sequenced the genome of *D. koepferae* to use as mapping reference for RNA-Seq reads since it was not previously available. To this end, genomic DNA was extracted from adult flies of genotype F combining two protocols to obtain DNA of high molecular weight and quality, suitable for high-throughput sequencing. The extraction was carried out using a purification protocol optimized for *D. melanogaster* (Gentra® Puregene® Cell Kit), and coupled with a standard Phenol:Chloroform extraction for cleaning-up the final extract.

DNA was first sequenced in an Illumina HiSeq 2000 platform using paired-end libraries (2x101 bp, insert size=400 bp) with a coverage > 100X (assuming a genome size similar to *D. buzzatii*). Next, using the same platform, a second sample was sequenced using a mate-pair library (2x50 bp, insert size=5 kbp) with a coverage > 25X. Paired-end library preparation and sequencing were carried out at Centre Nacional d'Anàlisi Genòmica (Barcelona, Spain), while mate-pair libraries were prepared and sequenced at the Centre de Regulació Genòmica (Barcelona, Spain). Finally, a third sample of the same genotype was sequenced at the University of Michigan's DNA Sequencing Core (Michigan, USA) in a Pacific Biosciences (PacBio) RS II platform employing 2 SMRT cells (P6/C4), using a library suitable to obtain an average read size of 10 kb and coverage > 5X.

C.3. Reads processing

All Illumina reads were quality controlled and processed accordingly. Instead, PacBio reads were quality controlled and properly processed by the sequencing service, so they were only filtered for a minimum length ≥ 5 kb. Raw Illumina data was analyzed with the program FASTQC v0.10.1 (Andrews 2010) for quality control. Next, the reads were filtered for a quality score ≥ 25 and a minimum length ≥ 25 bases using the program Trimmomatic v0.33 (Bolger et al. 2014). The Illumina reads from DNA samples were subsequently corrected with the program Lighter (Song et al. 2014) in order to amend any specific sequencing error, and then, any duplicated read was eliminated using the program FastUniq (Xu et al. 2012). The results obtained from these preprocessing steps are detailed in the next two tables:

Post-filtering WGS reads data for *D. kopeferae* genotype F

Technology	Illumina		PacBio
	Paired-end	Mate-pair	
Min read length (bp)	25	25	5000
Max read length (bp)	101	50	42029
Mean read length (bp)	99	49	15106
Mean insert size (bp)	336	4582	-
SD insert size (bp)	0,31	0,17	-
Total (bp)	35306672244	5874515837	951116834
Sequencing depth* (X)	220,67	36,72	5,94

*using a genome size of 160Mbp

RNA sequencing depth (post-filtering) for each genotype in the evaluated treatments.

Treatment	Post-filtering sequencing yield (Gbp)					
	D. buzzatii			*D. koepferae*		
	A	B	C	D	E	F
O. sulphurea 'Low nutrition'	4,62	4,71	0,00	5,08	5,78	0,00
O. sulphurea 'Native'	12,55	10,53	12,91	10,60	11,33	11,16
O. sulphurea '2X alkaloids'	11,09	14,17	14,96	14,61	12,83	13,42
T. terscheckii 'Low nutrition'	4,96	4,42	0,00	4,27	4,64	0,00
T. terscheckii 'Native'	9,53	11,82	23,82	11,72	10,33	11,82
T. terscheckii '2X alkaloids'	11,97	11,63	13,82	14,01	14,22	16,77

D.1. De novo genome assembly pipeline

For the *de novo* genome assembly protocol several programs for contigs assembly were tested, which, based on preliminary results, were reduced to three: ABySS v1.9.0 (Simpson et al. 2009), Platanus v1.2.4 (Kajitani et al. 2014) and SPAdes v3.8.0 (Bankevich et al. 2012). In the case of ABySS, contigs assemblies were made with different Kmer values (odd numbers from 31 to 45) obtaining the best result with a value of 43, while for Platanus and SPAdes the default parameters were used. The final assemblies were evaluated by comparing the parameters obtained by means of standard metrics and from the assembly quality value (REAPR score) reported by REAPR (Hunt et al. 2013). The best contigs assembly was achieved with SPAdes, since in addition to having the highest REAPR score, it had a higher N50 without any gap. In turn, an error checking step was performed with REAPR, whereby the incorrectly assembled contigs were cut in the joint errors. The results are detailed in the next table:

Contigs assembly using different programs. Parameters used to evaluate the quality of the assembly. In bold, the assembler that produced the best results (SPAdes) is highlighted

Assembler	ABySS	Platanus	**SPAdes**
Total length (bp)	176866604	150667435	201047147
Total contigs	46172	86478	55454
Longest contig (bp)	335905	52478	333284
N50 (bp)	6989	3413	19371
Total N's (bp)	641574	0	0
% N's	0,36	0	0
REAPR score	8,77E+11	3,17E+11	1,73E+12

The contigs produced with SPAdes of more than 300 bp in length (and corrected by REAPR splitting function), were decontaminated using the program dc-megablast (Zhang et al. 2000). In this step, the contigs were aligned to the genomes of seven *Drosophila* species (*D. buzzatii*, *D. mojavensis*, *D. virilis*, *D. grimshawi*, *D. pseudoobscura*, *D. sechellia* and *D. melanogaster*). All non-aligned contigs were discarded as considered possible contamination. The next step in the protocol was to reduce redundancy of contigs using the genomic alignment program LAST (Frith et al. 2010), in order to reduce assembly fragmentation. The following steps consisted of an iteration of contigs joining (scaffolding) followed by gaps closing, the latter using the program GapFiller v1.10 (Boetzer & Pirovano 2012). As for the scaffolding, on the one hand it was carried out with the program

SSPACE-longreads v1.1 (Boetzer & Pirovano 2014) using the reads obtained with PacBio technology, and on the other with the program SSPACE-standard (Boetzer et al. 2011) using reads produced from the Illumina sequencing with mate-pair and paired-end libraries. Once this step was completed, a new verification of assembly errors with REAPR was performed to cut the scaffolds where there were joining errors, and a second decontamination with dc-megablast using the genomes of the seven *Drosophila* species previously mentioned. Finally, an internal module of the ABySS assembler that uses transcriptomic information as evidence to carry out a scaffolding stage was used. In this way, we used a de novo transcriptomic assembly of the genotype F of *D. koepferae* in order to obtain a final assembly of scaffolds with a minimal length of 1 kb. The final assembly protocol is schematized in Figure S1.

D.2. De novo genotype F transcriptome assembly

After the quality control and filtering of all RNA-Seq reads, we performed a transcriptomic assembly of the genotype F of *D. koepferae*. The obtained transcriptome was used only for the genomic assembly protocol discussed above and for its subsequent structural annotation. The protocol consisted of the assembly of processed reads using the program Trinity (Grabherr et al. 2011) and a subsequent decontamination of the generated transcripts using dc-megablast. Thus, transcripts longer than 300 bp were aligned to four *Drosophila* reference genomes (*D. buzzatii*, *D. koepferae*, *D mojavensis* and *D. melanogaster*). All unaligned transcripts were discarded as possible contamination. Finally, the program RSEM v1.2.30 (Li & Dewey 2011) was used to estimate the expression of assembled transcripts as evidence of real transcripts, and thus, all transcripts with very low expression (FPKM below 0.5) were discarded. As a result, the genotype F transcriptome of *D. koepferae* contained 33936 transcripts, with the longest being 27456 bp and the mean length being 1490 bp.

E.1. Structural annotation

The structural annotation of the assembled *D. koepferae* genome was carried out with different tools available in the GenSAS platform. First, we performed a masking step of repetitive elements using RepeatMasker and RepeatModeler (tuned for *Drosophila*), for a final merged masking. Next, we annotated different features (other than protein-coding genes) such as tRNAs, rRNAs, SSRs and ORFs with the tools tRNAScan-SE, RNAmmer, SSR Finder and Getorf, respectively. The annotation of protein-coding genes was performed by merging through EvidenceModeler the different results of following tools. On the one hand, we used the gene predictors Augustus (using as evidence for training, the Uniprot

ecdysozoa proteins and the transcripts of *D. mojavensis*, *D. buzzatii*, and the de novo transcriptome assembly of the genotype F of *D. koepferae*, in different runs), SNAP (using the included pre-setting for *D. melanogaster*) and GeneMarkES. On the other hand, we used alignments data from nucleotide-Blast (of transcripts of *D. mojavensis*, *D. buzzatii*, and the de novo transcriptome assembly of the genotype F of *D. koepferae*, in different runs), protein-Blast (using the Uniprot ecdysozoa proteins), and BLAT (of transcripts of *D. mojavensis* and *D. buzzatii*, in different runs). In addition, a transcript reconstruction made with Cufflinks from the mapping of the genotype F reads to its assembled genome was added.

E.2. Functional annotation

The functional annotation of the protein-coding genes of *D. buzzatii* and *D. koepferae* was performed in the same way by Blast2GO, translating the structurally annotated genes in all the reading frames and performing an alignment by means of Blastx against Uniprot proteomes of, on the one hand *D. melanogaster*, and on the other the proteomes of 10 species of *Drosophila* (*D. simulans, D. melanogaster, D. grimshawi, D. sechellia, D. pseudoobscura, D. willistoni, D. ananassae, D. persimilis, D. virilis,* and *D. mojavensis*). At the same time, a search for functional motifs in the translated genes was performed using the different InterPro databases (InterProScan). Finally, through a search in the Gene Ontology database, the terms associated with each gene were annotated (Fig. S2). All these steps in the Blast2GO pipeline were run with default parameters. In this way, the excellent annotation available in *D. melanogaster* was used to assign identity and function to the genes of both species, in order to facilitate the understanding of the transcriptional landscape and the comparison of results. In addition, the use of the other species helped to fill any probable gap or solve possible bias in the assignment of GO terms in the annotation.

Using the functional annotation, the genes assigned to enzymes were classified into 6 classes using the standard code of the International Union of Biochemistry and Molecular Biology (IUBMB) according to the reactions they catalyze: EC.1 Oxidoreductases, EC.2 Transferases, EC.3 Hydrolases, EC.4 Lyases, EC.5 Isomerases, and EC.6 Ligases. The relative enzymatic profile was very similar in both species, although a slightly higher amount of Oxidoreductases, Transferases, and especially Hydrolases, was observed in *D. koepferae*.

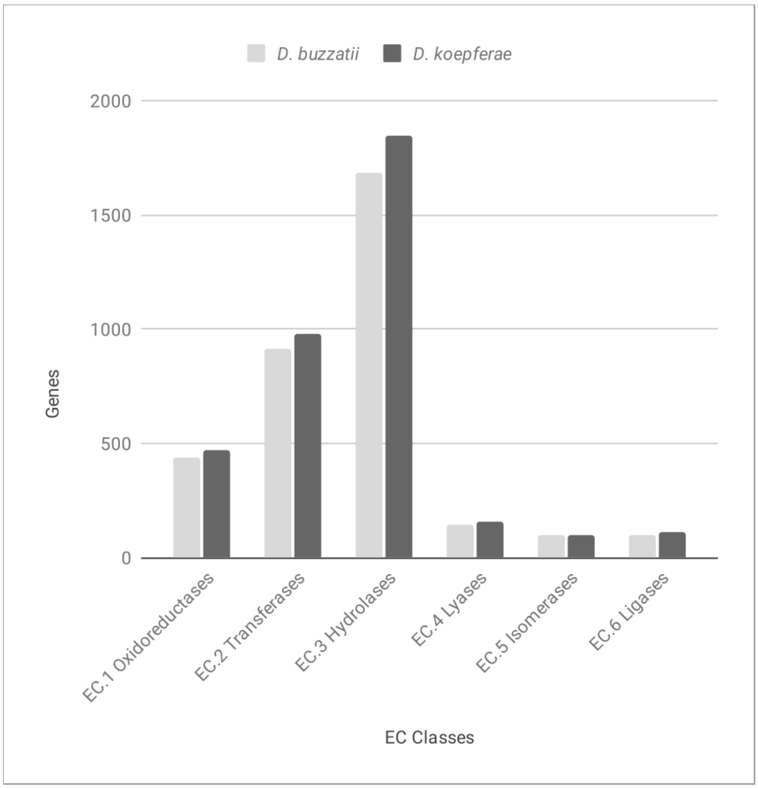

Genes assigned to enzymes classified into 6 classes using the standard code of the International Union of Biochemistry and Molecular Biology (IUBMB) according to its catalytic reaction from the functional annotation.

It should be noted that, although the *D. koepferae* genome has 4% more genes than *D. buzzatti* (567 more genes), it cannot be ruled out that this difference may be due to some annotation bias between both species. Therefore, the difference in the number of enzymes should be carefully considered. In any case, it is worth mentioning that within enzyme classes, there are some subclasses in particular that showed more pronounced differences, mainly with a higher number of enzymes in *D. koepferae*, but some cases were also observed in *D. buzzatii*.

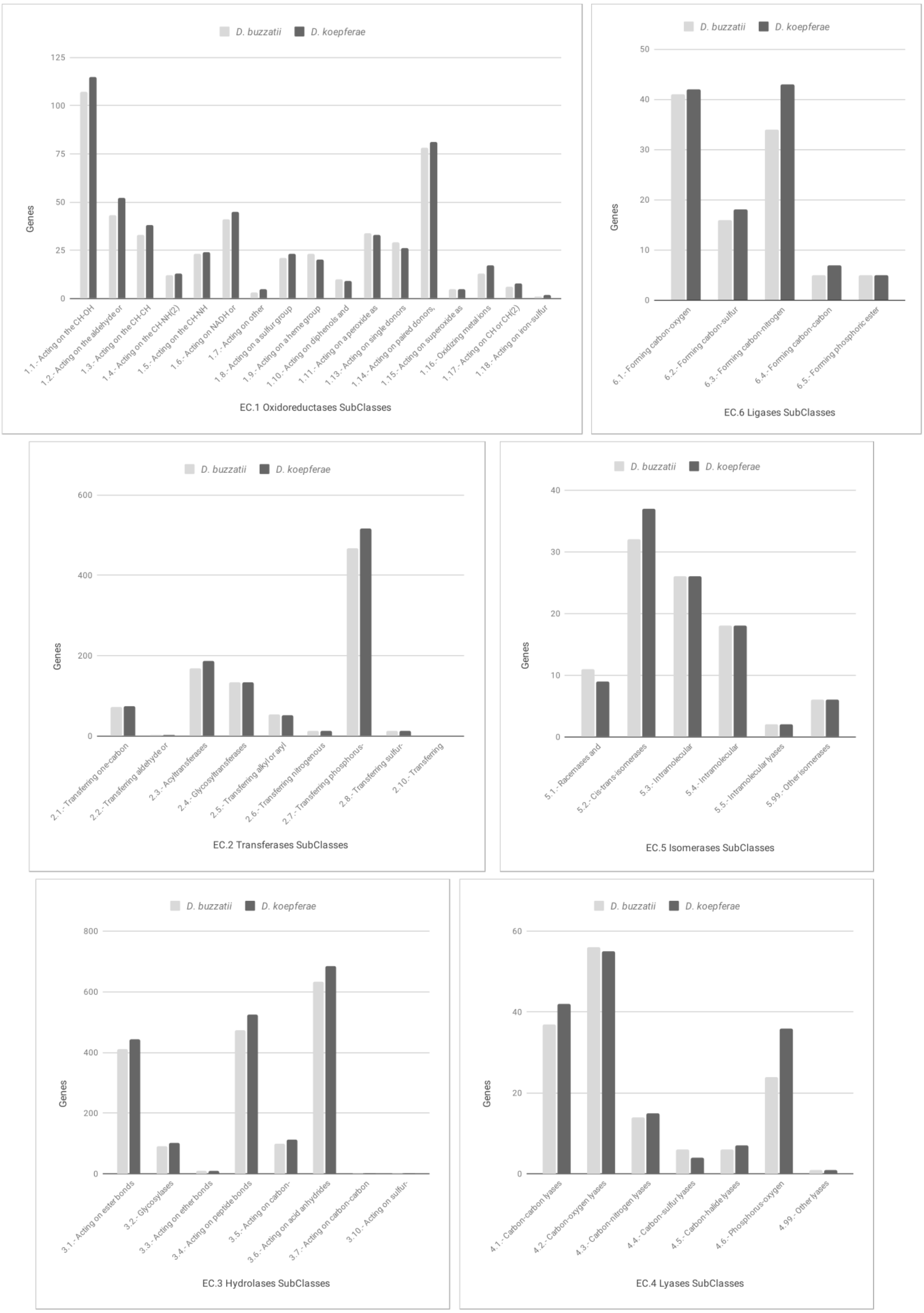

Genes assigned to enzymes classified into 6 classes using the standard code of the International Union of Biochemistry and Molecular Biology (IUBMB) according to its catalytic reaction from the functional annotation. Detail of each enzyme class.

F.1. RNA-seq reads mapping

We choose *D. mojavensis* as a phylogenetically equidistant reference for exploratory INTER-specific analyzes based on previous reports (Oliveira et al. 2012) and particularly based on results of a recent work from our group (Hurtado et al. 2019). In all the cases, the % of RNA-seq reads of each transcriptome mapped to the *D. mojavensis* reference genome was similar and within the 3rd quantile (>40-60%), as seen in the next table:

**Treatment**	**Genotypes**					
	D. buzzatii			***D. koepferae***		
	**A**	**B**	**C**	**D**	**E**	**F**
O. sulphurea 'Low nutrition'	49.56<br>42.64+6.89+0.03	47.9<br>42.64+5.22+0.04	-	48.85<br>41.91+6.90+0.04	47.12<br>41.47+5.61+0.04	-
O. sulphurea 'Native'	47.47<br>43.09+4.34+0.04	47.14<br>42.41+4.69+0.04	47.61<br>42.67+4.91+0.03	52.61<br>45.93+6.65+0.03	48.55<br>42.86+5.66+0.03	46.9<br>41.80+5.06+0.04
O. sulphurea '2X alkaloids'	48.57<br>43.90+4.64+0.03	46.77<br>42.51+4.22+0.04	46.14<br>40.87+5.23+0.04	49.51<br>43.25+6.22+0.04	46.19<br>41.69+4.46+0.04	49.12<br>44.02+5.05+0.05
T. terscheckii 'Low nutrition'	49.4<br>43.93+5.44+0.03	46.51<br>41.12+5.35+0.04	-	47.79<br>41.91+5.83+0.05	47.82<br>41.90+5.88+0.04	-
T. terscheckii 'Native'	45.51<br>41.28+4.16+0.07	45.16<br>40.92+4.20+0.04	46.77<br>41.30+5.43+0.04	48.14<br>42.97+5.12+0.05	48.46<br>43.63+4.78+0.05	46.74<br>42.42+4.27+0.05
T. terscheckii '2X alkaloids'	50.07<br>44.27+5.77+0.03	45.83<br>42.07+3.71+0.05	48.02<br>42.46+5.53+0.03	48.03<br>42.08+5.90+0.05	50.17<br>43.91+6.22+0.04	46.93<br>42.82+4.06+0.05

% of mapped reads (uniquely+multiple loci+too many loci)

We found no significant differences (p>0.1) between species in the mapping efficiency using the *D. mojavensis* genome as reference:

test: aov(lm(ARC_perc_uniquely ~ Species))

	Df	Sum Sq	Mean Sq	F value	Pr(>F)
mapping.efficiency$Species	1	0.000659	0.000659	2.841	0.102
Residuals	30	0.006959	0.000232		

Shapiro-Wilk normality test
data: Dmoj.mapping.efficiency$residuals
W = 0.95369, p-value = 0.1833

Bartlett test of homogeneity of variances
data: Dmoj.mapping$residuals by Dmoj.mapping$Species
Bartlett's K-squared = 0.07876, df = 1, p-value = 0.779

In addition, the quality of the alignments showed an average of 235.5 and 235.4 in *D. buzzatii* and *D. koepferae* genotypes, respectively, as seen in the next table:

**Treatment**	**Genotypes**					
	D. buzzatii			***D. koepferae***		
	**A**	**B**	**C**	**D**	**E**	**F**
O. sulphurea 'Low nutrition'	236.3428	236.4134	-	236.3508	236.0690	-
O. sulphurea 'Native'	235.3308	235.4490	235.5542	235.8063	235.3401	235.0106
O. sulphurea '2X alkaloids'	235.2032	235.0488	235.3475	234.7445	235.0288	234.7409
T. terscheckii 'Low nutrition'	236.5345	236.7255	-	236.7614	236.6526	-
T. terscheckii 'Native'	236.1051	236.0506	234.8147	236.4379	236.8372	235.5137
T. terscheckii '2X alkaloids'	235.2669	235.6447	235.1466	235.1934	235.3670	234.9888

STAR mapping quality mean

We found no significant differences between species in the mapping quality using the *D. mojavensis* genome as reference:

test: aov(lm(mapping.quality ~ Species))

	Df	Sum Sq	Mean Sq	F value	Pr(>F)
mapping.quality$Species	1	0.0010	0.0006	0.001	0.972
Residuals	30	13.481	0.4494		

Shapiro-Wilk normality test
data: Dmoj.mapping.quality$residuals
W = 0.92153, p-value = 0.02288

Bartlett test of homogeneity of variances
data: Mapping_quality_mean by Species
Bartlett's K-squared = 0.72744, df = 1, p-value = 0.3937

Moreover, by means of a PCA produced by Qualimap using the Coverage, GC percentage, Mean mapping quality and Median insert size of the genotypes mapped to *D. mojavensis*, we found no clustering by species, as seen in the next plot (DB: *D. buzzatii*; DK: *D. koepferae*):

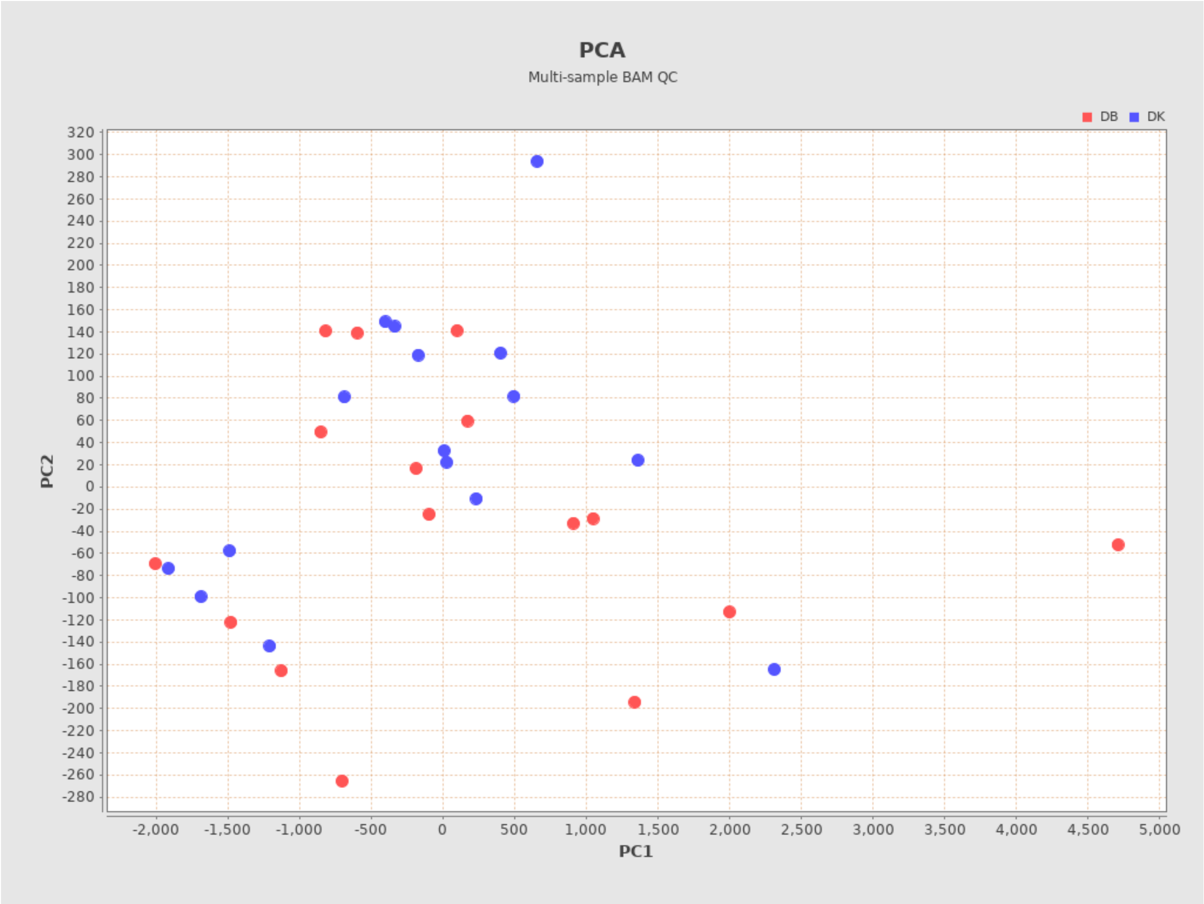

Unsurprisingly, we observed that not normalized coverage influences the clustering of the samples. This source of bias is addressed by the TMM normalization during the differential expression analysis.

Regarding the % of RNA-seq reads of each transcriptome mapped to its respective reference genome for INTRA-specific analyzes, all were similar and within the 5th quantile (>80-100%). These results are detailed in the next two tables:

Treatment	*D. buzzatii* genotype		
	A	B	C
O. sulphurea 'Low nutrition'	88.0	89.4	-
O. sulphurea 'Native'	90.8	90.2	90.1
O. sulphurea '2X alkaloids'	90.3	90.6	90.1
T. terscheckii 'Low nutrition'	89.2	88.5	-
T. terscheckii 'Native'	90.7	89.5	89.6
T. terscheckii '2X alkaloids'	89.8	91.5	89.5

% of uniquely mapped reads, overall mean±SD: 89.9±0.9

Treatment	*D. koepferae* genotype		
	D	E	F
O. sulphurea 'Low nutrition'	88.0	89.7	-
O. sulphurea 'Native'	88.1	89.6	91.5
O. sulphurea '2X alkaloids'	89.1	90.7	91.6
T. terscheckii 'Low nutrition'	88.9	88.5	-
T. terscheckii 'Native'	89.7	89.7	92.1
T. terscheckii '2X alkaloids'	90.3	85.0	92.7

% of uniquely mapped reads, overall mean±SD: 89.7±1.9

F.2. Differential gene expression analyzes

The detailed scripts for intra and inter-species differential gene expression analyzes is available on Github (https://github.com/diegomics/CompRNAseq_paper2020). In the treatments where a condition with 2 biological replicates is compared with a condition with 3 biological replicates, we removed the missing replicate for performing the analysis (genotypes C and F for *D. buzzatii* and *D. koepferae*, respectively). Therefore, the comparisons were always 2vs2, or 3vs3 biological replicates depending on the case, experiment-wide. In the case of INTER-specific analysis, the comparisons for the treatments *O. sulphurea* 'Low nutrition' and *T. terscheckii* 'Low nutrition' used two biological replicates (genotypes A,B vs D,E). For INTRA-specific analysis, the comparisons that used two biological replicates (genotypes A,B for *D. buzzatii*, and D,E for *D. koepferae*) were between cacti for the 'Low nutrition' condition and between 'Low nutrition' and 'Native' conditions for each cactus. The `NOISeqBIO` method implemented in the `R` package `NOISeq v2.18` (Tarazona et al. 2015) uses two statistics differential expression measurement: a log-ratio of average expression values for the two compared conditions and the difference between expression values across conditions. These statistics are corrected by the variability among biological replicates. In this work, Tarazona *et al.* show that the performance of `NOISeqBIO` in simulated scenarios (considering different numbers of genes, replicates per condition, levels of technical noise, proportions of DEGs, and high and low biological variability), combined an average sensitivity (i.e. true DE calls out of the total number of DEGs) of 90% and 95% and FDR slightly above 5% and lower than 5% with two and three replicates per condition, respectively.

In INTER-specific analyzes, the expression of a species in a given treatment is compared against that of the other species in the same treatment, for example: *D. buzzatii* in *O. sulphurea* '2X alkaloids' vs *D. koepferae* in *O. sulphurea* ' 2X alkaloids'. Thus, if a gene is overexpressed in *D. buzzatii* in this comparison, it means that it is expressed more than the same gene of *D. koepferae* in that comparison. The same goes for *D. koepferae*, if a gene is overexpressed in that species, that means that it is more expressed that the same gene in *D. buzzatii* for that particular treatment. Therefore, the overexpression of a gene in one species is always relative to the expression in the other species. The identity of the gene used by both species in INTER-specific analyzes is based on the annotation of the reference genome, in this case, the equidistant *D. mojavensis*.

In INTRA-specific comparisons, the amount of DE genes between treatments is related, on the one hand, to how a species perceives each treatment, and on the other, with the transcriptional response that is capable of producing in each case. If the difference in expression between treatments is small, it is likely that the compared conditions mobilize a similar transcriptional response because the organism perceives apparently different conditions as very similar, and to a lesser extent, to its inability to generate a response. The few DE genes in these cases may be part of a response that is finer and sensitive to some of the environmental components in which the conditions evaluated differ. The opposite occurs when the difference in expression between treatments is not small, suggesting that the organism responds in distinctive ways to alternative environmental challenges. The response in this case may be evenly distributed across conditions, or geared towards one in particular. The latter case may occur due to the overexpression of sets of genes related to the response to specific conditions of the environment (*e.g.* detoxification genes due to the presence of some toxic compound), or because a condition causes the arrest of the expression of some genes exposing the differences with a default transcriptional program.

From the expression scores of each one of the DEGs, each combination of treatment and biological replicate can be conceived as a point in a multidimensional expression space, which in turn can be reduced to 3 dimensions by means of the Multi-Dimensional Scaling method (MDS). This method that uses the structure of global similarity between DEGs to construct a space in which the data structure can be represented, facilitates the visualization and interpretation of transcriptomic similarity across treatments and replicates. The `R` packages `SMACOF` and `RGL` were used to perform different MDS and its visualization.

Functional enrichment analyzes of Gene Ontology (GO) terms and biological pathways were performed on the DE gene sets across the evaluated comparisons. For these analyzes the extensive functional data of *D. melanogaster* was used through the program `g:Profiler` (Reimand et al. 2007). In addition, intraspecific analyzes also used the functional data obtained for each species with `Blast2GO` and analyzed with the `R` package `goseq v1.26.0` (Young et al. 2010), and on the other hand the program `WEGO 2.0` (Ye et al. 2018) was used for categorical visualization. We also employed `REViGO` (Supek et al. 2011) to summarize the GO enrichment lists by removing redundant terms, using the D. melanogaster GO terms database and an allowed similarity (SimRel) ≤ 0.4. Additionally, we manually inspected the results to look for extra redundancy when possible, and to remove too general terms.

G. Developmental time

The experiment consisted of 5 vials (experimental units) per genotype (biological replicates, here called line) per treatment, adding a total of 180 vials.

All statistical analyses and respective visualizations were carried out in `R` using the packages `PHIA` (De Rosario-Martinez 2015), `CAR` (Fox & Weisberg 2011), `LME4` (Bates et al. 2015), `INFLUENCE.ME` (Nieuwenhuis et al. 2012), `MULTCOMP` (Hothorn et al. 2008) and `GGPLOT2` (Wickham et al. 2016).

Complete dataset:

First, we fitted a GLMM to analyze differences in Developmental time (DT) among treatments in both *Drosophila* species. Vials were used as experimental units (mean DT per replicate as dependent variable), and “Cactus” and “Condition” as fixed crossed factors. We verified fitting to a gamma distribution through the following plot of sample quantiles (Y axis) vs theoretical quantiles (X axis) in which the 95% confidence interval is shown:

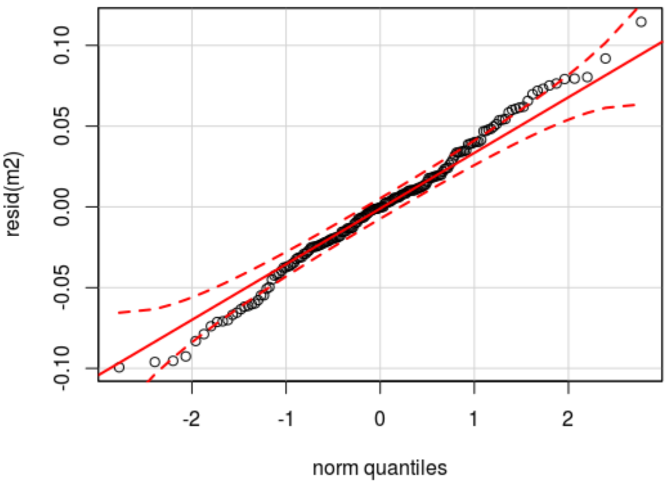

We also verified homoscedasticity by plotting sample residues (Y axis) on adjusted residues (X axis), as follows:

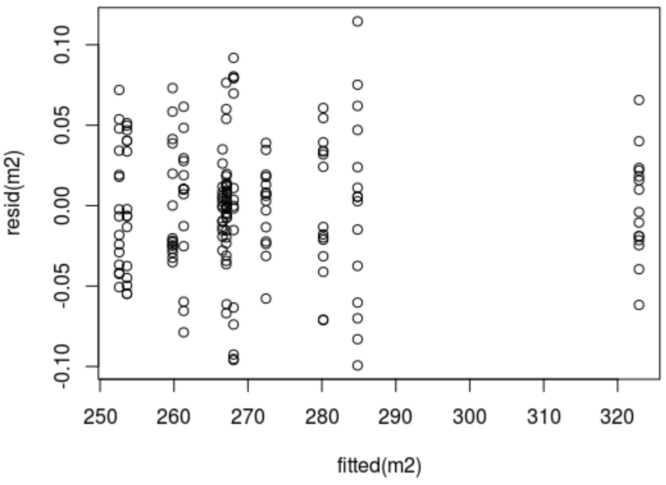

The results of the ANOVA are shown in the following table:

Response: DT_mean

	LR Chisq	Df	Pr(>Chisq)
Cactus	1.684	1	0.1944331
Condition	25.375	2	3.089e-06 ***
Species	0.000	1	0.9891831
Cactus:Condition	35.848	2	1.644e-08 ***
Cactus:Species	12.558	1	0.0003945 ***
Condition:Species	6.325	2	0.0423113 *
Cactus:Condition:Species	0.414	2	0.8130941

Significancy: 0 ‘***’ 0.001 ‘**’ 0.01 ‘*’ 0.05 ‘.’ 0.1 ‘ ’ 1

Based on these results, we decided to analyze DT data of each fly species separately, modelling the levels in each case and selecting the model with the best balance between explanatory power (measured as the joint probability of the data under the chosen parameters) and complexity (measured in the number of parameters that fit the model). For *D. koepferae* the best model included a random slope for “Condition” and a random intercept for “Line” (AIC = 574,046), while the best model for *D. buzzatii* included a random slope for “Cactus” and a random intercept for the combination of “Line” and “Condition” (AIC = 611,291). Unlike the model selected to analyze DT data in *D. koepferae*, the assumptions of normality and homoscedasticity were not met by the *D. buzzatii* model, therefore the data was fitted to a gamma distribution instead of a gaussian distribution.

D. koepferae dataset:

Fitting to a normal distribution for *D. koepferae* data was accomplished by the following plot of sample quantiles (Y axis) vs theoretical quantiles (X axis) in which the 95% confidence interval is shown:

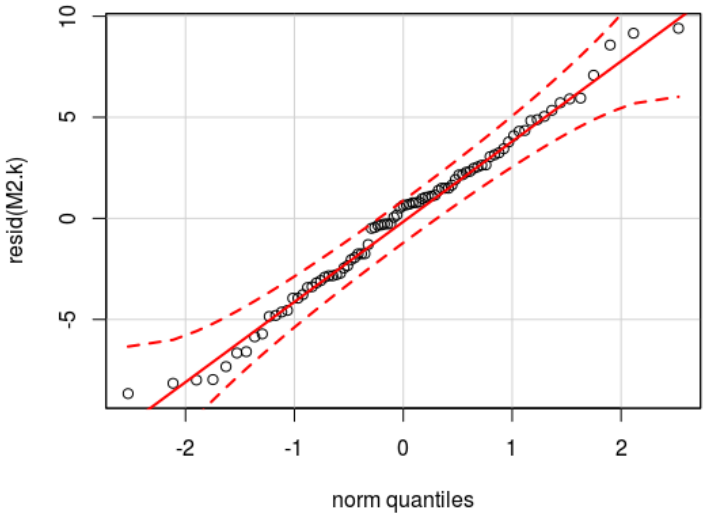

In addition, we verified the fit to a normal distribution through the Shapiro-Wilk test:

data: resid(M2)

W = 0.9881, p-value = 0.6151

We also verified the assumption of homoscedasticity by means of Levene test:

	Df	F value	Pr(>F)
Group	5	1.1783	0.3271
	81		

Next, it is shown the significance of the inclusion of random terms:

M1: DT_mean ~ Cactus * Condition

M2: DT_mean ~ Cactus * Condition + (Condition | Line)

	Df	AIC	BIC	logLik	deviance	Chisq Chi	Df	Pr(>Chisq)
M1	7	702.64	719.90	-344.32	688.64			
M2	13	539.39	571.45	-256.70	513.39	175.25	6	< 2.2e-16 ***

Significancy: 0 ‘***’ 0.001 ‘**’ 0.01 ‘*’ 0.05 ‘.’ 0.1 ‘ ’ 1

The results of the corresponding ANOVA are shown in the following table:

Response: DT_mean

	Chisq	Df	Pr(>Chisq)
(Intercept)	1734.3815	1	< 2e-16 ***
Cactus	86.7790	1	< 2e-16 ***
Condition	7.5759	2	0.02264 *
Cactus:Condition	182.0961	2	< 2e-16 ***

Significancy: 0 ‘***’ 0.001 ‘**’ 0.01 ‘*’ 0.05 ‘.’ 0.1 ‘ ’ 1

D. buzzatii dataset:

We verified the fit to a gamma distribution for *D. buzzatii* data by the following plot of sample quantiles (Y axis) vs. theoretical quantiles (X axis) in which the 95% confidence interval is shown:

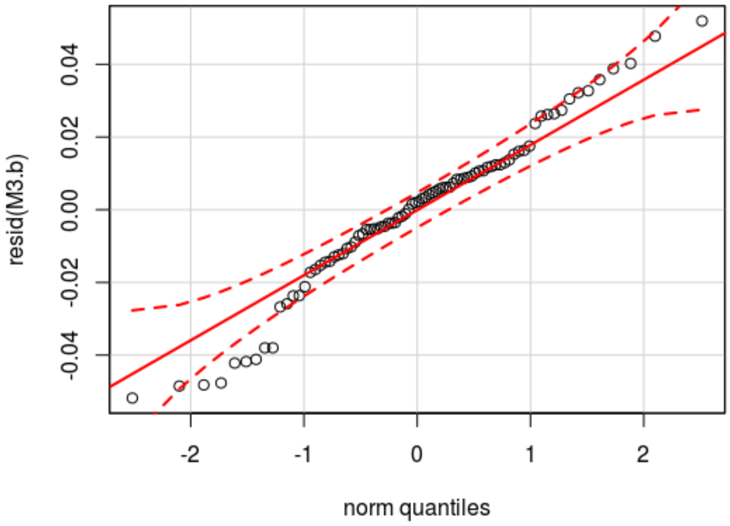

We also verified homoscedasticity through the following plot of sample residues (Y axis) vs. adjusted residues (X axis):

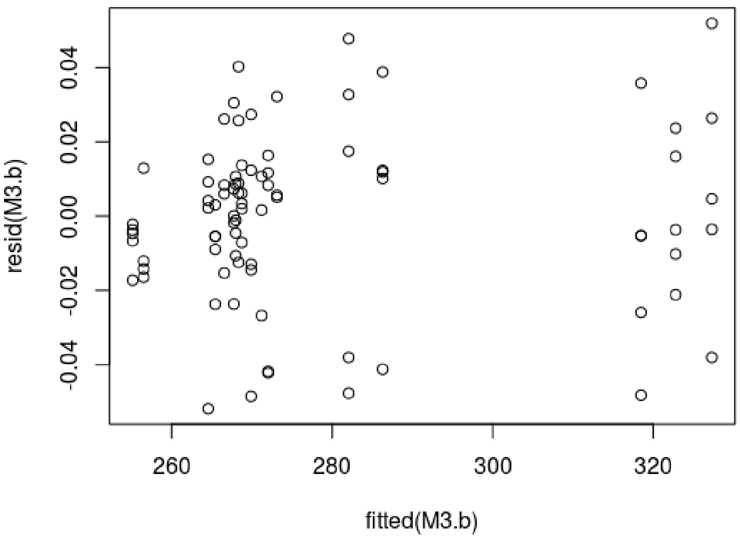

Next, it is shown the significance of the inclusion of random terms:

M1: DT_mean ~ Cactus * Condition

M2: DT_mean ~ Cactus * Condition + (Cactus | Line:Condition)

	Df	AIC	BIC	logLik	deviance	Chisq Chi	Df	Pr(>Chisq)
M1	7	607.44	624.46	-296.72	593.44			
M2	10	583.34	607.65	-281.67	563.34	30.1	3	1.315e-06 ***

Significancy: 0 ‘***’ 0.001 ‘**’ 0.01 ‘*’ 0.05 ‘.’ 0.1 ‘ ’ 1

The ANOVA resulted as shown in the following table:

Response: DT_mean

	Chisq	Df	Pr(>Chisq)
(Intercept)	17571.5284	1	< 2.2e-16 ***
Cactus	2.9705	1	0.0847948 .
Condition	14.6565	2	0.0006567 ***
Cactus:Condition	37.1882	2	8.408e-09 ***

Significancy: 0 ‘***’ 0.001 ‘**’ 0.01 ‘*’ 0.05 ‘.’ 0.1 ‘ ’ 1

For the analysis of the nutritional component, the best models for each species were the same as those previously fitted, with the difference that both fitted to a gamma distribution because the assumptions for gaussian distribution were not met.

D. buzzatii nutritional component dataset:

The verification of the fitting to a gamma distribution for *D. buzzatii* (only nutritional component) through the following plot of sample quantiles (Y axis) vs. theoretical quantiles (X axis) in which the 95% confidence interval is shown next:

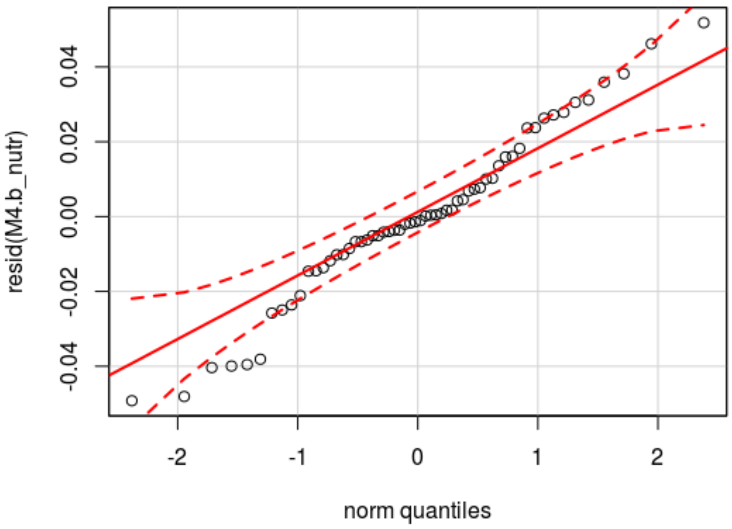

We also verified homoscedasticity through the following plot of sample residues (Y axis) vs. adjusted residues (X axis):

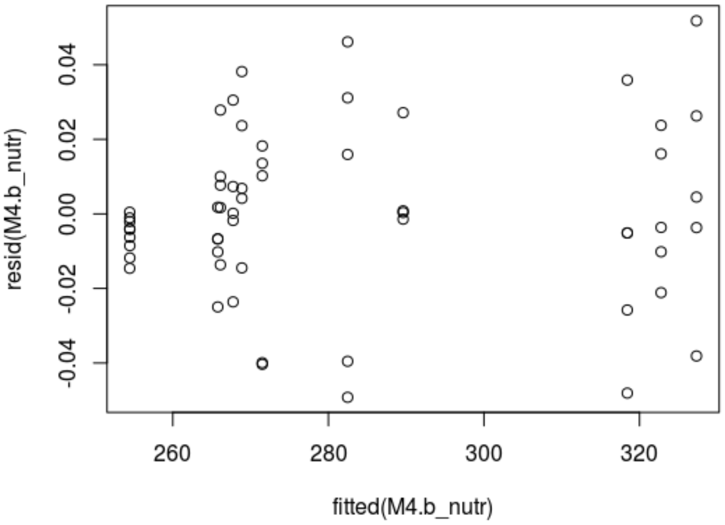

Next, it is shown the significance of the inclusion of random terms:

M1: DT_mean ~ Cactus * Condition

M2: DT_mean ~ Cactus * Condition + (Cactus | Line:Condition)

	Df	AIC	BIC	logLik	deviance	Chisq Chi	Df	Pr(>Chisq)
M1	5	433.56	443.86	-211.78	423.56			
M2	8	408.12	424.60	-196.06	392.12	31.439	3	6.87e-07 ***

Significancy: 0 ‘***’ 0.001 ‘**’ 0.01 ‘*’ 0.05 ‘.’ 0.1 ‘ ’ 1

The ANOVA resulted as shown in the following table:

Response: DT_mean

	Chisq	Df	Pr(>Chisq)
(Intercept)	15143.6897	1	< 2.2e-16 ***
Cactus	9.5393	1	0.002011 **
Condition	41.0241	1	1.504e-10 ***
Cactus:Condition	33.2105	1	8.270e-09 ***

Significancy: 0 ‘***’ 0.001 ‘**’ 0.01 ‘*’ 0.05 ‘.’ 0.1 ‘ ’ 1

D. koepferae nutritional component dataset:

The verification of the fitting to a gamma distribution for *D. koepferae* (only nutritional component) through the following plot of sample quantiles (Y axis) vs. theoretical quantiles (X axis) in which the 95% confidence interval is shown next:

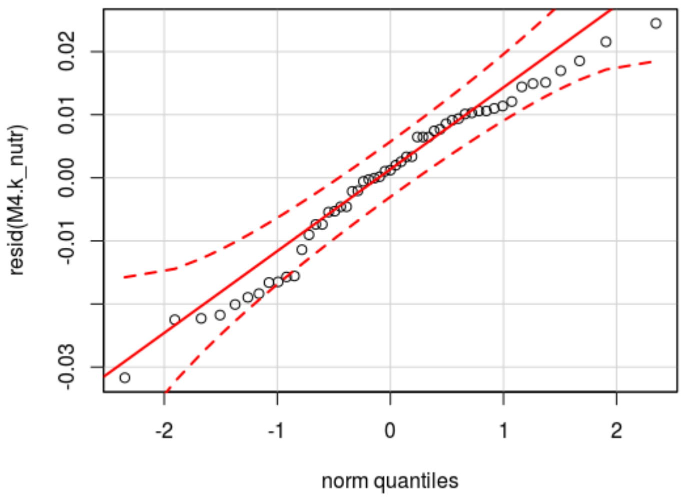

We also verified homoscedasticity through the following plot of sample residues (Y axis) vs. adjusted residues (X axis):

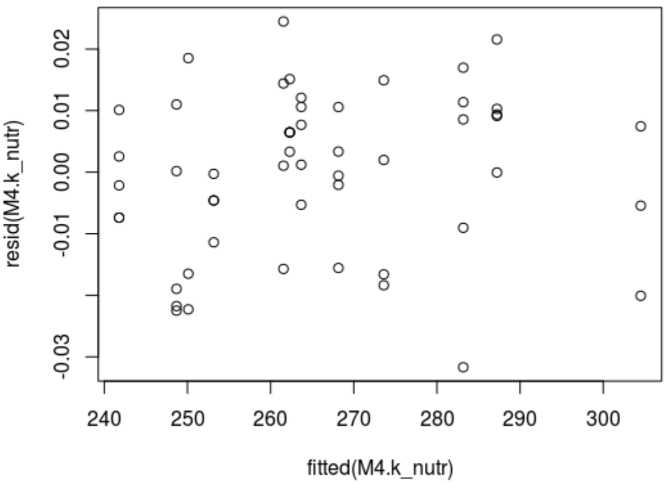

Next, it is shown the significance of the inclusion of random terms:

M1: DT_mean ~ Cactus * Condition

M2: DT_mean ~ Cactus * Condition + (Condition | Line)

	Df	AIC	BIC	logLik	deviance	Chisq Chi	Df	Pr(>Chisq)
M1	5	439.12	448.97	-214.56	429.12			
M2	8	308.08	323.84	-146.04	292.08	137.04	3	< 2.2e-16 ***

Significancy: 0 ‘***’ 0.001 ‘**’ 0.01 ‘*’ 0.05 ‘.’ 0.1 ‘ ’ 1

The ANOVA resulted as shown in the following table:

Response: DT_mean

	Chisq	Df	Pr(>Chisq)
(Intercept)	7505.1964	1	< 2.2e-16 ***
Cactus	66.4973	1	3.504e-16 ***
Condition	7.4107	1	0.006484 **
Cactus:Condition	259.7600	1	< 2.2e-16 ***

Significancy: 0 ‘***’ 0.001 ‘**’ 0.01 ‘*’ 0.05 ‘.’ 0.1 ‘ ’ 1

In contrast, for the chemical component, the specific random terms for the best model for *D. buzzatii* included a random intercept for both the combination of “Line” and “Cactus”, and for the combination of “Line” and “Condition” (AIC = 371.958). At the same time, the best model for *D. koepferae* included a random intercept for “Line” (AIC = 363.162). Both models met the assumptions of normality and homoscedasticity to fit to a gaussian distribution.

D. buzzatii chemical component dataset:

The fit to a normal distribution for *D. buzzatii* (chemical component) through the following plot of sample quantiles (Y axis) vs. theoretical quantiles (X axis) in which the 95% confidence interval is shown next:

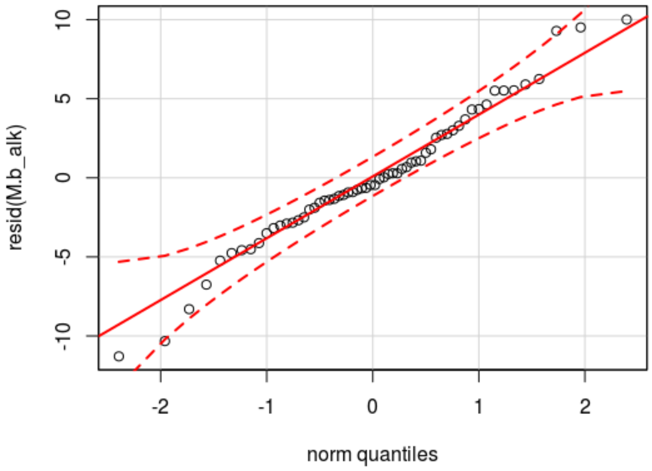

In addition, we verified the adjustment to a normal distribution using Shapiro-Wilk test:

data: resid(M2)

W = 0.9796, p-value = 0.4118

We also verified the assumption of homoscedasticity through the Levene test:

	Df	F value	Pr(>F)
Group	3	0.7268	0.5403
	56		

Next, we show the significance of the inclusion of random terms:

M1: DT_mean ~ Cactus * Condition

M2: DT_mean ~ Cactus * Condition + (1 | Line:Cactus) + (1 | Line:Condition)

	Df	AIC	BIC	logLik	deviance	Chisq Chi	Df	Pr(>Chisq)
M1	5	406.62	417.09	-198.31	396.62			
M2	7	387.81	402.47	-186.91	373.81	22.812	2	1.113e-05 ***

Significancy: 0 ‘***’ 0.001 ‘**’ 0.01 ‘*’ 0.05 ‘.’ 0.1 ‘ ’ 1

The ANOVA resulted as shown in the following table:

Response: DT_mean

	Chisq	Df	Pr(>Chisq)
(Intercept)	4676.5595	1	< 2e-16 ***
Cactus	1.9556	1	0.16199
Condition	2.8206	1	0.09306 .
Cactus:Condition	0.3548	1	0.55143

Significancy: 0 ‘***’ 0.001 ‘**’ 0.01 ‘*’ 0.05 ‘.’ 0.1 ‘ ’ 1

D. koepferae chemical component dataset:

The verification of the fitting to a normal distribution for *D. koepferae* (chemical component only) through the following plot of sample quantiles (Y axis) vs. theoretical quantiles (X axis) in which the 95% confidence interval is shown next:

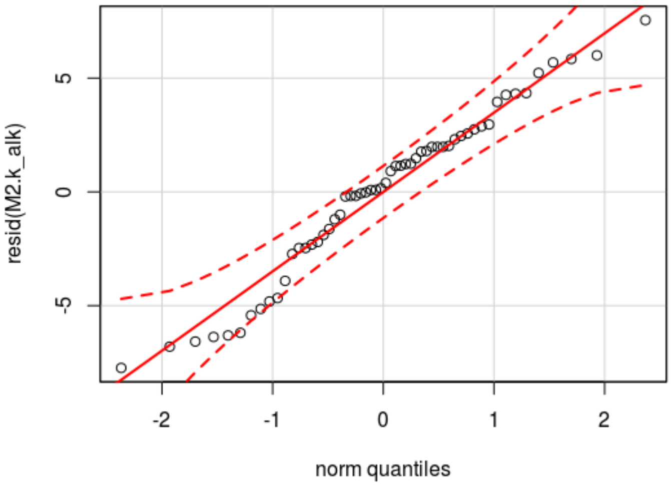

In addition, we verified the adjustment to a normal distribution through the Shapiro-Wilk test:

data: resid(M2)

W = 0.96896, p-value = 0.1574

We also verified the assumption of homoscedasticity through the Levene test:

	Df	F value	Pr(>F)
Group	3	1.8578	0.1482
	52		

Next, it is shown the significance of the inclusion of random terms:

M1: DT_mean ~ Cactus * Condition

M2: DT_mean ~ Cactus * Condition + (1 | Line)

	Df	AIC	BIC	logLik	deviance	Chisq Chi	Df	Pr(>Chisq)
M1	5	421.21	431.34	-205.61	411.21			
M2	6	333.55	345.70	-160.78	321.55	89.659	1	< 2.2e-16 ***

Significancy: 0 '***' 0.001 '**' 0.01 '*' 0.05 '.' 0.1 ' ' 1

The ANOVA resulted as shown in the following table:

Response: DT_mean

	Chisq	Df	Pr(>Chisq)
(Intercept)	1782.6587	1	< 2e-16 ***
Cactus	90.8852	1	< 2e-16 ***
Condition	5.9941	1	0.01435 *
Cactus:Condition	3.5696	1	0.05885 .

--- Significancy: 0 '***' 0.001 '**' 0.01 '*' 0.05 '.' 0.1 ' ' 1

H. References

Andrews, S. (2010). FastQC: a quality control tool for high throughput sequence data.

Aranda, P. S., LaJoie, D. M., & Jorcyk, C. L. (2012). Bleach gel: a simple agarose gel for analyzing RNA quality. *Electrophoresis*, 33(2), 366-369.

Ashburner, M. (1989). Drosophila. A laboratory handbook. Cold spring harbor laboratory press.

Bankevich, A., Nurk, S., Antipov, D., Gurevich, A. A., Dvorkin, M., Kulikov, A. S., ... & Pyshkin, A. V. (2012). SPAdes: a new genome assembly algorithm and its applications to single-cell sequencing. *Journal of computational biology*, 19(5), 455-477.

Bates, D., Mächler, M., Bolker, B., & Walker, S. (2015). Fitting Linear Mixed-Effects Models Using lme4. 2015 67, 48.

Boetzer, M., Henkel, C. V., Jansen, H. J., Butler, D., & Pirovano, W. (2011). Scaffolding pre-assembled contigs using SSPACE. *Bioinformatics*, 27(4), 578-579.

Boetzer, M., & Pirovano, W. (2012). Toward almost closed genomes with GapFiller. *Genome biology*, 13(6), R56.

Boetzer, M., & Pirovano, W. (2014). SSPACE-LongRead: scaffolding bacterial draft genomes using long read sequence information. *BMC bioinformatics*, 15 (1), 211.

Bogart, K., & Andrews, J. (2006). Extraction of total RNA from Drosophila. Center for Genomics and Bioinformatics *CGB Technical Report*, 10, 1-4.

Bolger, AM, Lohse, M., & Usadel, B. (2014). Trimmomatic: a flexible trimmer for Illumina sequence data. *Bioinformatics*, 30 (15), 2114-2120.

De Panis, D. N., Padró, J., Furió‐Tarí, P., Tarazona, S., Milla Carmona, P. S., Soto, I. M., ... & Hasson, E. (2016). Transcriptome modulation during host shift is driven by secondary metabolites in desert *Drosophila*. *Molecular ecology*, 25(18), 4534-4550.

De Rosario-Martinez, H. (2015). phia: Post-hoc interaction analysis. R package version 0.2-1.

Fox, J., & Weisberg, S. (2011). Car: companion to applied regression Available at: http: // CRAN. R-project. org / package = car Accessed, 20.

Frith, M. C., Hamada, M., & Horton, P. (2010). Parameters for accurate genome alignment. *BMC bioinformatics*, 11(1), 80.

Grabherr, M. G., Haas, B. J., Yassour, M., Levin, J. Z., Thompson, D. A., Amit, I., ... & Chen, Z. (2011). Trinity: reconstructing a full-length transcriptome without a genome from RNA-Seq data. *Nature biotechnology*, 29(7), 644.

Hothorn, T., Bretz, F., Westfall, P., & Heiberger, R. M. (2008). Multcomp: simultaneous inference for general linear hypotheses. R package version, 1-0.

Hunt, M., Kikuchi, T., Sanders, M., Newbold, C., Berriman, M., & Otto, T. D. (2013). REAPR: a universal tool for genome assembly evaluation. *Genome biology*, 14(5), R47.

Hurtado, J. P., Almeida, F., Revale, S., & Hasson, E. (2019). Revised phylogenetic relationships within the Drosophila buzzatii species cluster (Diptera: Drosophilidae: Drosophila repleta group) using genomic data. *Arthropod Systematics and Phylogeny*, *77*(2).

Kajitani, R., Toshimoto, K., Noguchi, H., Toyoda, A., Ogura, Y., Okuno, M., ... & Kohara, Y. (2014). Efficient de novo assembly of highly heterozygous genomes from whole-genome shotgun short reads. *Genome research*, 24(8), 1384-1395.

Li, B., & Dewey, C. N. (2011). RSEM: accurate transcript quantification from RNA-Seq data with or without a reference genome. *BMC bioinformatics*, 12(1), 323.

Nieuwenhuis, R., Te Grotenhuis, H. F., & Pelzer, B. J. (2012). Influence. ME: tools for detecting influential data in mixed effects models.

Oliveira, DC, Almeida, FC, O'Grady, PM, Armella, MA, DeSalle, R., & Etges, WJ (2012). Monophyly, divergence times, and evolution of host plant use inferred from a revised phylogeny of the Drosophila repleta species group. *Molecular Phylogenetics and Evolution* , *64* (3), 533-544.

Reimand, J., Kull, M., Peterson, H., Hansen, J., & Vilo, J. (2007). g: Profiler—a web-based toolset for functional profiling of gene lists from large-scale experiments. *Nucleic acids research*, 35(suppl_2), W193-W200.

Roig-Juñent, S., Flores, G., Claver, S., Debandi, G., & Marvaldi, A. (2001). Monte Desert (Argentina): insect biodiversity and natural areas. *Journal of Arid Environments*, 47(1), 77-94.

Saint Esteven, A., Benedictto, M., Garolla, F. A., Padró, J., & Soto, I. M. (2021). A survey of cacti richness in a biodiversity hotspot of Western Argentina. *Bradleya*, 2021(39), 5-15.

Simpson, J. T., Wong, K., Jackman, S. D., Schein, J. E., Jones, S. J., & Birol, I. (2009). ABySS: a parallel assembler for short read sequence data. *Genome research*, 19(6), 1117-1123.

Song, L., Florea, L., & Langmead, B. (2014). Lighter: fast and memory-efficient sequencing error correction without counting. *Genome biology*, 15(11), 509.

Supek, F., Bošnjak, M., Škunca, N., & Šmuc, T. (2011). REVIGO summarizes and visualizes long lists of gene ontology terms. *PloS one*, *6*(7), e21800.

Wickham, H., Chang, W., & Wickham, M. H. (2016). Package 'ggplot2'. Create Elegant Data Visualisations Using the Grammar of Graphics. Version, 2(1), 1-189.

Xu, H., Luo, X., Qian, J., Pang, X., Song, J., Qian, G., ... & Chen, S. (2012). FastUniq: a fast de novo duplicates removal tool for paired short reads. *PloS one*, 7(12), e52249.

Ye, J., Zhang, Y., Cui, H., Liu, J., Wu, Y., Cheng, Y., ... & Zhang, X. (2018). WEGO 2.0: a web tool for analyzing and plotting GO annotations, 2018 update. *Nucleic acids research*, 46(W1), W71-W75.

Young, M. D., Wakefield, M. J., Smyth, G. K., & Oshlack, A. (2010). Gene ontology analysis for RNA-seq: accounting for selection bias. *Genome biology*, 11(2), R14.

Zhang, Z., Schwartz, S., Wagner, L., & Miller, W. (2000). A greedy algorithm for aligning DNA sequences. *Journal of Computational biology*, 7 (1-2), 203-214.
