# Supplementary material for: Transcriptional responses are oriented towards different components of the rearing environment in two Drosophila sibling species
Source: BMC Genomics. 2022 Jul 16;23:515. doi: 10.1186/s12864-022-08745-9 (PMC9288027; doi:10.1186/s12864-022-08745-9)
Supplement: Supplementary file 2 — Additional file 2. Supporting tables: TableS1-S7. [file 12864_2022_8745_MOESM2_ESM.pdf]

Table S1. Summarized functional profiling based on gene ontology (GO) enrichment analysis and pathways overrepresentation (q<0.05) for exploratory INTER-specific analyses. The name of each species in a particular treatment is followed by categorized enriched terms within the respective relative overexpressed gene set.

Treatment		GO terms enrichment* Biological process	GO terms enrichment* Cellular component	GO terms enrichment* Molecular function	Pathway overrepresentation**
O. sulphurea 'Low nutrition'	*D. buzzatii*	-	GO:0022626 'cytosolic ribosome' [7]; GO:0044391 'ribosomal subunit' [7]	-	-
	D. koepferae	-	-	-	-
O. sulphurea 'Native'	*D. buzzatii*	GO:0006820 'anion transport' [25]; GO:0007623 'circadian rhythm' [23]; GO:0008063 'Toll signaling pathway' [15]; GO:0009056 'catabolic process' [79]; GO:0009072 'aromatic amino acid family metabolic process' [7]; GO:0042335 'cuticle development' [29]; GO:0043473 'pigmentation' [17]; GO:0046189 'phenol-containing compound biosynthetic process' [8]; GO:0048066 'developmental pigmentation' [17]; GO:0071702 'organic substance transport' [65]; GO:1901565 'organonitrogen compound catabolic process' [49]	GO:0000502 'proteasome complex' [12]; GO: 0005576 'extracellular region' [88]; GO:0005737 'cytoplasm' [227]; GO:1905369 'endopeptidase complex' [12]	GO:0003824 'catalytic activity' [207]; GO:0008483 'transaminase activity' [5]; GO:0016769 'transferase activity, transferring nitrogenous groups' [5]; GO:0022804 'active transmembrane transporter activity' [24]	KEGG:03050 'Proteasome' [11]; KEGG:00270 'Cysteine and methionine metabolism' [7]; KEGG: 00350 'Tyrosine metabolism' [6]; WP: 281 'Proteasome Degradation' [10]
	D. koepferae	GO:0002181 'cytoplasmic translation' [25]; GO: 0009071 'serine family amino acid catabolic process' [4]; GO:0043603 'cellular amide metabolic process' [42]; GO:0046034 'ATP metabolic process' [16]; GO: 0017144 'drug metabolic process' [34]; GO: 1901564 'organonitrogen compound metabolic process' [135]	GO:0005737 'cytoplasm' [184]; GO:0005739 'mitochondrion' [50]; GO:0005743 'mitochondrial inner membrane' [19]; GO:0005829 'cytosol' [59]; GO:0005840 'ribosome' [24]; GO:0030017 'sarcomere' [8]; GO:0043228 'non-membrane-bounded organelle' [74]; GO:0098798 'mitochondrial protein complex' [20]; GO:1990204 'oxidoreductase complex' [11]; GO:1990904 'ribonucleoprotein complex' [37]	GO:0003723 'RNA binding' [42]; GO:0003735 'structural constituent of ribosome' [24]; GO: 0005198 'structural molecule activity' [41]; GO: 0008135 'translation factor activity, RNA binding' [10]; GO:0045182 'translation regulator activity' [12]	KEGG:00260 'Glycine, serine and threonine metabolism' [6]; KEGG: 03010 'Ribosome' [23]; WP:537 'Translation Factors' [9]
O. sulphurea '2X alkaloids'	*D. buzzatii*	GO:0006575 'cellular modified amino acid metabolic process' [22]; GO:0006749 'glutathione metabolic process' [15]; GO:0006790 'sulfur compound metabolic process' [27]; GO:0006820 'anion transport' [36]; GO:0006979 'response to oxidative stress' [23]; GO:0009056 'catabolic process' [122]; GO:0009636 'response to toxic substance' [37]; GO:0015833 'peptide transport' [65]; GO:0042440 'pigment metabolic process' [24]; GO:0042737 'drug catabolic process' [16]; GO:0043473 'pigmentation' [22]; GO:0071705 'nitrogen compound transport' [78]; GO:0071985 'multivesicular body sorting pathway' [11]; GO: 0098754 'detoxification' [18]	GO:0005576 'extracellular region' [122]; GO: 0005737 'cytoplasm' [385]; GO:0005764 'lysosome' [20]; GO:0005773 'vacuole' [27]; GO: 0070993 'translation preinitiation complex' [8]; GO: 0097708 'intracellular vesicle' [48]	GO:0003824 'catalytic activity' [341]; GO:0004364 'glutathione transferase activity' [12]; GO:0016209 'antioxidant activity' [13]; GO:0016491 'oxidoreductase activity' [68]; GO:0016765 'transferase activity, transferring alkyl or aryl (other than methyl) groups' [18]; GO:0048037 'cofactor binding' [47]	KEGG:00480 'Glutathione metabolism' [18]; KEGG:00790 'Folate biosynthesis' [10]; KEGG:04142 'Lysosome' [21]; WP:281 'Proteasome Degradation' [8]
	D. koepferae	GO:0017144 'drug metabolic process' [31]; GO: 0030029 'actin filament-based process' [24]; GO: 0032502 'developmental process' [116]; GO: 0040003 'chitin-based cuticle development' [17]; GO:0042692 'muscle cell differentiation' [19]; GO: 0044085 'cellular component biogenesis' [69]; GO: 0046716 'muscle cell cellular homeostasis' [7]; GO: 0048856 'anatomical structure development' [109]; GO:0061061 'muscle structure development' [26]; GO:0070925 'organelle assembly' [26]; GO: 0097435 'supramolecular fiber organization' [21]	GO:0030016 'myofibril' [19]; GO:0019866 'organelle inner membrane' [20]; GO:0031966 'mitochondrial membrane' [21]; GO:0043228 'non-membrane-bounded organelle' [76]; GO:0045169 'fusome' [6]; GO:0005737 'cytoplasm' [167]; GO: 0005829 'cytosol' [48]; GO:0015629 'actin cytoskeleton' [19]	GO:0003779 'actin binding' [20]; GO:0005198 'structural molecule activity' [41]; GO:0008092 'cytoskeletal protein binding' [25]; GO:0046933 'proton-transporting ATP synthase activity, rotational mechanism' [5]	KEGG:00190 'Oxidative phosphorylation' [13]; WP:542 'Electron Transport Chain' [10]
T. terscheckii 'Low nutrition'	*D. buzzatii*	GO:0006574 'valine catabolic process' [2]	GO:0005576 'extracellular region' [15]; GO: 0044444 'cytoplasmic part' [27]	GO:0004075 'biotin carboxylase activity' [2]; GO: 0016885 'ligase activity, forming carbon-carbon bonds' [2]	KEGG:00071 'Fatty acid degradation' [3]; KEGG:00280 'Valine, leucine and isoleucine degradation' [4]; KEGG: 00640 'Propanoate metabolism' [3]; WP:568 'Fatty Acid Biosynthesis' [3]
	D. koepferae	GO:0007623 'circadian rhythm' [7]; GO:0048511 'rhythmic process' [7]	GO:0000502 'proteasome complex' [4]; GO: 1905369 'endopeptidase complex' [4]	-	KEGG:03050 'Proteasome' [3]; WP: 281 'Proteasome Degradation' [4]
T. terscheckii 'Native'	*D. buzzatii*	GO:0002181 'cytoplasmic translation' [28]; GO: 0006575 'cellular modified amino acid metabolic process' [18]; GO:0009056 'catabolic process' [94]; GO:0009636 'response to toxic substance' [27]; GO:0043604 'amide biosynthetic process' [48]; GO:0055114 'oxidation-reduction process' [66]; GO:1901564 'organonitrogen compound metabolic process' [230]	GO:0000502 'proteasome complex' [15]; GO: 0005576 'extracellular region' [100]; GO:0005737 'cytoplasm' [291]; GO:0005829 'cytosol' [86]; GO: 0005840 'ribosome' [26]; GO:0070993 'translation preinitiation complex' [8]; GO:1905368 'peptidase complex' [15];	GO:0003735 'structural constituent of ribosome' [23]; GO:0003824 'catalytic activity' [263]; GO: 0008483 'transaminase activity' [6]; GO:0016209 'antioxidant activity' [10]; GO:0016491 'oxidoreductase activity' [61]; GO:0016765 'transferase activity, transferring alkyl or aryl (other than methyl) groups' [12]	KEGG:00270 'Cysteine and methionine metabolism' [9]; KEGG: 00480 'Glutathione metabolism' [17]; KEGG:03050 'Proteasome' [12]; WP: 281 'Proteasome Degradation' [9]
	D. koepferae	GO:0032787 'monocarboxylic acid metabolic process' [22]; GO:0055001 'muscle cell development' [13]	GO:0005700 'polytene chromosome' [25]; GO: 0005737 'cytoplasm' [238]; GO:0030016 'myofibril' [10]; GO:0099080 'supramolecular complex' [25]	GO:0004197 'cysteine-type endopeptidase activity' [8]; GO:0051015 'actin filament binding' [10];	KEGG:04512 'ECM-receptor interaction' [5]
T. terscheckii '2X alkaloids'	*D. buzzatii*	GO:0006575 'cellular modified amino acid metabolic process' [20]; GO:0006631 'fatty acid metabolic process' [21]; GO:0006749 'glutathione metabolic process' [14]; GO:0006790 'sulfur compound metabolic process' [25]; GO:0055114 'oxidation-reduction process' [95]; GO:0017144 'drug metabolic process' [48]	GO:0005576 'extracellular region' [109]; GO: 0005737 'cytoplasm' [310]; GO:0070993 'translation preinitiation complex' [8]	GO:0020037 'heme binding' [20]; GO:0003824 'catalytic activity' [309]; GO:0004035 'alkaline phosphatase activity' [7]; GO:0004364 'glutathione transferase activity' [10]; GO:0016491 'oxidoreductase activity' [87]; GO:0016765 'transferase activity, transferring alkyl or aryl (other than methyl) groups' [15]	KEGG:00270 'Cysteine and methionine metabolism' [10]; KEGG: 00480 'Glutathione metabolism' [19]; KEGG:01100 'Metabolic pathways' [92]
	D. koepferae	GO:0006950 'response to stress' [116]; GO: 0007292 'female gamete generation' [71]; GO: 0009888 'tissue development' [100]; GO:0032502 'developmental process' [250]; GO:0048513 'animal organ development' [111]; GO:0048585 'negative regulation of response to stimulus' [51]; GO:0048699 'generation of neurons' [89]; GO: 0048731 'system development' [157]; GO:0048856 'anatomical structure development' [234]; GO: 0050896 'response to stimulus' [254]; GO:0055001 'muscle cell development' [14]; GO:0060429 'epithelium development' [91]	GO:0000502 'proteasome complex' [15]; GO: 0005700 'polytene chromosome' [33]; GO: 0005737 'cytoplasm' [317]; GO:0005829 'cytosol' [89]; GO:0030864 'cortical actin cytoskeleton' [7]; GO:0043228 'non-membrane-bounded organelle' [130]; GO:0045169 'fusome' [13]; GO:0062023 'collagen-containing extracellular matrix' [7]; GO: 0097708 'intracellular vesicle' [41]; GO:1905369 'endopeptidase complex' [15]	GO:0005515 'protein binding' [201]; GO: 0005198 'structural molecule activity' [55]	KEGG:04141 'Protein processing in endoplasmic reticulum' [21]; KEGG: 04512 'ECM-receptor interaction' [5]; WP:281 'Proteasome Degradation' [11]

*The order is as follows: GO term ID 'GO term name' [number of DE genes annotated with that term]. **The order is as follows: KEGG pathway ID or WP pathway ID 'pathway name' [number of DE genes that mapped to that pathway].

Table S2. INTRA-specific gene expression analyses in *D. buzzatii*. The name of each treatment in a particular comparison is followed by the number of overexpressed genes in that condition (q<0.01).

	Pairwise comparison between cacti for each treatment	Overexpressed genes
1	*O. sulphurea* 'Low nutrition'	7
	T. terscheckii 'Low nutrition'	24
2	*O. sulphurea* 'Native'	60
	T. terscheckii 'Native'	10
3	*O. sulphurea* '2X alkaloids'	2
	T. terscheckii '2X alkaloids'	1

Three *a priori* planned INTRA-specific between cacti comparisons (1: exploratory 'Low nutrition', 2:'Native', 3:'2X alkaloids')

	Pairwise comparison between treatment for each cactus	Overexpressed genes
1	*O. sulphurea* 'Low nutrition'	13
	O. sulphurea 'Native'	33
2	*O. sulphurea* 'Native'	80
	O. sulphurea '2X alkaloids'	57
3	*T. terscheckii* 'Low nutrition'	38
	T. terscheckii 'Native'	9
4	*T. terscheckii* 'Native'	7
	T. terscheckii '2X alkaloids'	10

Four *a priori* planned INTRA-specific between treatments comparisons (1: exploratory *O. sulphurea*/Nutritional component, 2:*O. sulphurea*/Chemical component, 3:exploratory *T. terscheckii*/Nutritional component, 4:*T. terscheckii*/Chemical component)

Table S3. INTRA-specific gene expression analyses in *D. koepferae*. The name of each treatment in a particular comparison is followed by the number of overexpressed genes in that condition (q<0.01).

	Pairwise comparison between cacti for each treatment	Overexpressed genes
1	*O. sulphurea* 'Low nutrition'	27
	T. terscheckii 'Low nutrition'	31
2	*O. sulphurea* 'Native'	97
	T. terscheckii 'Native'	52
3	*O. sulphurea* '2X alkaloids'	115
	T. terscheckii '2X alkaloids'	124

Three *a priori* planned INTRA-specific between cacti comparisons (1: exploratory 'Low nutrition', 2:'Native', 3:'2X alkaloids')

	Pairwise comparison between treatment for each cactus	Overexpressed genes
1	*O. sulphurea* 'Low nutrition'	14
	O. sulphurea 'Native'	135
2	*O. sulphurea* 'Native'	6
	O. sulphurea '2X alkaloids'	12
3	*T. terscheckii* 'Low nutrition'	9
	T. terscheckii 'Native'	39
4	*T. terscheckii* 'Native'	7
	T. terscheckii '2X alkaloids'	15

Four *a priori* planned INTRA-specific between treatments comparisons (1: exploratory *O. sulphurea*/Nutritional component, 2:*O. sulphurea*/Chemical component, 3:exploratory *T. terscheckii*/Nutritional component, 4:*T. terscheckii*/Chemical component)

Table S4. Summarized functional profiling based on gene ontology enrichment analysis and enzyme code enrichment (q<0.05) of differentially expressed genes for *D. buzzatti* INTRA-specific analyses

D. buzzatii						
	Pairwise comparison between cacti for each treatment	GO terms enrichment* Biological process	Cellular component	Molecular function	Enzyme code enrichment**
1	*O. sulphurea* 'Low nutrition'	-	-	-	-
1	*T. terscheckii* 'Low nutrition'	GO:0055114 'oxidation-reduction process' [9]	-	GO:0004497 'monooxygenase activity' [4]; GO:0020037 'heme binding' [6]	-
2	*O. sulphurea* 'Native'	GO:0006094 'gluconeogenesis' [3]; GO: 0006096 'glycolytic process' [4]; GO: 0007507 'heart development' [4]; GO: 0019543 'propionate catabolic process' [2]; GO:0033993 'response to lipid' [2]; GO:0043149 'stress fiber assembly' [3]; GO:0055093 'response to hyperoxia' [3]	GO:0001725 'stress fiber' [3]; GO: 0005616 'larval serum protein complex' [2]; GO:0016529 'sarcoplasmic reticulum' [3]	GO:0045735 'nutrient reservoir activity' [2]; GO:0004613 'phosphoenolpyruvate carboxykinase (GTP) activity' [2]; GO: 0051015 'actin filament binding' [6]	EC:4.1.1.32 'phosphoenolpyruvate carboxykinase' [2]
2	*T. terscheckii* 'Native'	-	-	-	-
3	*O. sulphurea* '2X alkaloids'	-	-	-	-
3	*T. terscheckii* '2X alkaloids'	-	-	-	-

Three *a priori* planned INTRA-specific between cacti comparisons (1:exploratory 'Low nutrition', 2:'Native', 3:'2X alkaloids')

*The order is as follows: GO term ID 'GO term name' [number of DE genes annotated with that term]. **The order is as follows: Enzyme code number 'enzyme name' [number of DE genes annotated with that enzyme code].

D. buzzatii					
	Pairwise comparison between treatment for each cactus	GO terms enrichment* Biological process	Cellular component	Molecular function	Enzyme code enrichment**
1	*O. sulphurea* 'Low nutrition'	-	-	-	-
1	*O. sulphurea* 'Native'	GO:0055114 'oxidation-reduction process' [9]	-	-	-
2	*O. sulphurea* 'Native'	GO:0040003 'chitin-based cuticle development' [8]	-	GO:0042302 'structural constituent of cuticle' [4]	-
2	*O. sulphurea* '2X alkaloids'	GO:0006069 'ethanol oxidation' [3]; GO: 0006117 'acetaldehyde metabolic process' [3]; GO:0006749 'glutathione metabolic process' [6]; GO:0017143 'insecticide metabolic process' [2]; GO: 0048252 'lauric acid metabolic process' [2]; GO:0055114 'oxidation-reduction process' [17]	GO:0005789 'endoplasmic reticulum membrane' [8]	GO:0020037 'heme binding' [9]; GO: 0004364 'glutathione transferase activity' [6]; GO:0004497 'monooxygenase activity' [6]; GO:0004602 'glutathione peroxidase activity' [3]; GO:0008774 'acetaldehyde dehydrogenase (acetylating) activity' [3]; GO:0018685 'alkane 1-monooxygenase activity' [2]	EC:2.5.1.18 'glutathione S-transferase' [6]; EC:1.1.1.1 'alcohol dehydrogenase' [3]; EC:1.2.1.10 'acetaldehyde dehydrogenase (acetylating)' [3]; EC:1.11.1.9 'glutathione peroxidase' [3]; EC: 1.14.15 'oxidoreductase' [2]
3	*T. terscheckii* 'Low nutrition'	-	-	-	-
3	*T. terscheckii* 'Native'	GO:0006574 'valine catabolic process' [2]; GO:0055114 'oxidation-reduction process' [9]; GO:0099132 'ATP hydrolysis coupled cation transmembrane transport' [3]	GO:0005739 'mitochondrion' [7]	-	-
4	*T. terscheckii* 'Native'	-	-	-	-
4	*T. terscheckii* '2X alkaloids'	GO:0055114 'oxidation-reduction process' [5]	GO:0005789 'endoplasmic reticulum membrane' [4]	GO:0004497 'monooxygenase activity' [3]; GO:0020037 'heme binding' [5]	-

Four *a priori* planned INTRA-specific between treatments comparisons (1:exploratory *O. sulphurea*/Nutritional component, 2:*O. sulphurea*/Chemical component, 3:exploratory *T. terscheckii*/Nutritional component, 4:*T. terscheckii*/Chemical component)

*The order is as follows: GO term ID 'GO term name' [number of DE genes annotated with that term]. **The order is as follows: Enzyme code number 'enzyme name' [number of DE genes annotated with that enzyme code].

Table S5. Summarized functional profiling based on gene ontology enrichment analysis and enzyme code enrichment (q<0.05) of differentially expressed genes for *D. koepferae* INTRA-specific analyses

D. koepferae					
	Pairwise comparison between cacti for each treatment	GO terms enrichment*			Enzyme code enrichment**
		Biological process	Cellular component	Molecular function	
1	*O. sulphurea* 'Low nutrition'	-	-	-	-
1	*T. terscheckii* 'Low nutrition'	GO:0055114 'oxidation-reduction process' [10]	GO:0005789 'endoplasmic reticulum membrane' [6]	GO:0020037 'heme binding' [6]	-
2	*O. sulphurea* 'Native'	GO:0002181 'cytoplasmic translation' [16]; GO:0006094 'gluconeogenesis' [3]; GO: 0008363 'larval chitin-based cuticle development' [5]; GO:0019543 'propionate catabolic process' [2]; GO: 0033993 'response to lipid' [2]	GO:0005840 'ribosome' [5]	GO:0003735 'structural constituent of ribosome' [22]; GO:0004613 'phosphoenolpyruvate carboxykinase (GTP) activity' [2]	EC:4.1.1.32 'phosphoenolpyruvate carboxykinase' [2]
2	*T. terscheckii* 'Native'	GO:0001692 'histamine metabolic process [2]; GO:0042136 'neurotransmitter biosynthetic process [2]; GO:0048022 'negative regulation of melanin biosynthetic process [2]; GO: 0048082 'regulation of adult chitin-containing cuticle pigmentation [4]	-	GO:0000036 'acyl carrier activity' [2]; GO: 0003833 'beta-alanyl-dopamine synthase activity' [2]; GO:0004511 'tyrosine 3-monooxygenase activity' [2]	EC:1.14.16.2 'tyrosine 3-monooxygenase' [2]
3	*O. sulphurea* '2X alkaloids'	GO:0001666 'response to hypoxia' [6]; GO:0006096 'glycolytic process' [5]; GO: 0007498 'mesoderm development' [8]; GO:0007527 'adult somatic muscle development' [6]; GO:0008363 'larval chitin-based cuticle development' [8]; GO: 0009636 'response to toxic substance' [4]; GO:0034620 'cellular response to unfolded protein' [5]; GO:0042026 'protein refolding' [8]; GO:0042593 'glucose homeostasis' [5]; GO:0043149 'stress fiber assembly' [2]	GO:0005840 'ribosome' [4]; GO:0031012 'extracellular matrix' [14]; GO:0032982 'myosin filament' [4]	GO:0008307 'structural constituent of muscle' [8]; GO:0030898 'actin-dependent ATPase activity' [4]; GO:0031072 'heat shock protein binding' [5]; GO:0042302 'structural constituent of cuticle' [4]; GO: 0044183 'protein binding involved in protein folding' [5]	-
3	*T. terscheckii* '2X alkaloids'	GO:0006585 'dopamine biosynthetic process from tyrosine' [3]; GO:0048082 'regulation of adult chitin-containing cuticle pigmentation' [5]	-	-	-

Three *a priori* planned INTRA-specific between cacti comparisons (1:exploratory 'Low nutrition', 2:'Native', 3:'2X alkaloids')

*The order is as follows: GO term ID 'GO term name' [number of DE genes annotated with that term]. **The order is as follows: Enzyme code number 'enzyme name' [number of DE genes annotated with that enzyme code].

D. koepferae					
	Pairwise comparison between treatment for each cactus	GO terms enrichment*			Enzyme code enrichment**
		Biological process	Cellular component	Molecular function	
1	*O. sulphurea* 'Low nutrition'	GO:0006585 'dopamine biosynthetic process from tyrosine' [2]; GO:0006726 'eye pigment biosynthetic process' [2]; GO:0009611 'response to wounding' [2]; GO:0042136 'neurotransmitter biosynthetic process' [2]; GO:0042542 'response to hydrogen peroxide' [2]	-	GO:0004505 'phenylalanine 4-monooxygenase activity' [2]; GO:0004511 'tyrosine 3-monooxygenase activity' [2]	EC:1.14.16.1 'phenylalanine 4-monooxygenase' [2]; EC:1.14.16.2 'tyrosine 3-monooxygenase' [2]
1	*O. sulphurea* 'Native'	GO:0002181 'cytoplasmic translation' [12]; GO:0006094 'gluconeogenesis' [4]; GO: 0019464 'glycine decarboxylation via glycine cleavage system' [3]; GO:0055114 'oxidation-reduction process' [22]	GO:0005739 'mitochondrion' [12]; GO: 0005960 'glycine cleavage complex' [3]; GO:0022627 'cytosolic small ribosomal subunit' [6]	GO:0030170 'pyridoxal phosphate binding' [6]; GO:0003735 'structural constituent of ribosome' [14]	-
2	*O. sulphurea* 'Native'	-	-	-	-
2	*O. sulphurea* '2X alkaloids'	-	-	-	-
3	*T. terscheckii* 'Low nutrition'	-	-	-	-
3	*T. terscheckii* 'Native'	-	-	-	-
4	*T. terscheckii* 'Native'	-	-	-	-
4	*T. terscheckii* '2X alkaloids'	-	-	-	-

Four *a priori* planned INTRA-specific between treatments comparisons (1:exploratory *O. sulphurea*/Nutritional component, 2:*O. sulphurea*/Chemical component, 3:exploratory *T. terscheckii*/Nutritional component, 4:*T. terscheckii*/Chemical component)

*The order is as follows: GO term ID 'GO term name' [number of DE genes annotated with that term]. **The order is as follows: Enzyme code number 'enzyme name' [number of DE genes annotated with that enzyme code].

Table S6. Summarized functional profiling based on gene ontology enrichment analysis and pathways overrepresentation (q<0.05) of *D. melanogaster* homolog genes for *D. buzzatti* INTRA-specific analyses

	D. buzzatii				
	Pairwise comparison between cacti for each treatment	GO terms enrichment* Biological process	Cellular component	Molecular function	Pathway overrepresentation**
1	*O. sulphurea* 'Low nutrition'	-	-	-	-
1	*T. terscheckii* 'Low nutrition'	GO:0006117 'acetaldehyde metabolic process' [2]; GO:0009636 'response to toxic substance' [5]; GO:0034308 'primary alcohol metabolic process' [2]; GO:0055114 'oxidation-reduction process' [10]	GO:0005789 'endoplasmic reticulum membrane' [5]	GO:0004022 'alcohol dehydrogenase (NAD) activity' [2]; GO:0004497 'monooxygenase activity' [5]; GO:0008774 'acetaldehyde dehydrogenase (acetylating) activity' [2]; GO:0016491 'oxidoreductase activity' [10]; GO:0020037 'heme binding' [6]	KEGG:00010 'Glycolysis / Gluconeogenesis' [3]; KEGG:00071 'Fatty acid degradation' [3]; KEGG:00350 'Tyrosine metabolism' [2]; KEGG:00830 'Retinol metabolism' [3]; KEGG:00980 'Metabolism of xenobiotics by cytochrome P450' [5]; KEGG:00981 'Insect hormone biosynthesis' [2]
2	*O. sulphurea* 'Native'	GO:0003012 'muscle system process' [8]; GO:0005975 'carbohydrate metabolic process' [10]; GO:0006090 'pyruvate metabolic process' [7]; GO:0006733 'oxidoreduction coenzyme metabolic process' [5]; GO:0006757 'ATP generation from ADP' [5]; GO:0007498 'mesoderm development' [6]; GO:0007519 'skeletal muscle tissue development' [3]; GO:0009743 'response to carbohydrate' [4]; GO:0016052 'carbohydrate catabolic process' [5]; GO:0019432 'triglyceride biosynthetic process' [3]; GO:0030029 'actin filament-based process' [11]; GO:0042593 'glucose homeostasis' [6]; GO:0042866 'pyruvate biosynthetic process' [5]; GO:0048646 'anatomical structure formation involved in morphogenesis' [12]; GO:0048856 'anatomical structure development' [26]; GO:0048878 'chemical homeostasis' [11]; GO:0061061 'muscle structure development' [13]; GO:0072524 'pyridine-containing compound metabolic process' [5]; GO:0097435 'supramolecular fiber organization' [10]	GO:0005616 'larval serum protein complex' [2]; GO:0005737 'cytoplasm' [31]; GO:0005829 'cytosol' [15]; GO:0005856 'cytoskeleton' [11]; GO:0016529 'sarcoplasmic reticulum' [3]; GO:0043228 'non-membrane-bounded organelle' [19]; GO:0099512 'supramolecular fiber' [17];	GO:0045735 'nutrient reservoir activity' [2]; GO:0003779 'actin binding' [8]; GO:0004613 'phosphoenolpyruvate carboxykinase (GTP) activity' [2]; GO:0005198 'structural molecule activity' [12]; GO:0008092 'cytoskeletal protein binding' [11]; GO:0008307 'structural constituent of muscle' [5]; GO:0036094 'small molecule binding' [15]	KEGG:00010 'Glycolysis / Gluconeogenesis' [8]; KEGG:00030 'Pentose phosphate pathway' [3]; KEGG:00051 'Fructose and mannose metabolism' [3]; KEGG:01200 'Carbon metabolism' [6]; KEGG:01230 'Biosynthesis of amino acids' [5]; WP:144 'Glycolysis and Gluconeogenesis' [6]
2	*T. terscheckii* 'Native'	-	-	GO:0070330 'aromatase activity' [1]	-
3	*O. sulphurea* '2X alkaloids'	-	-	-	-
3	*T. terscheckii* '2X alkaloids'	GO:0019428 'allantoin biosynthetic process' [1]; GO:0019628 'urate catabolic process' [1]; GO:0046415 'urate metabolic process' [1]	-	GO:0004846 'urate oxidase activity' [1]; GO:0016661 'oxidoreductase activity, acting on other nitrogenous compounds as donors' [1]	KEGG:00232 'Caffeine metabolism' [1]

Three *a priori* planned INTRA-specific between cacti comparisons (1:exploratory 'Low nutrition', 2:'Native', 3:'2X alkaloids')

*The order is as follows: GO term ID '*GO term name*' [number of DE genes annotated with that term]. **The order is as follows: KEGG pathway ID or WP pathway ID 'pathway name' [number of DE genes that mapped to that pathway].

	D. buzzatii				
	Pairwise comparison between treatment for each cactus	GO terms enrichment* Biological process	Cellular component	Molecular function	Pathway overrepresentation**
1	*O. sulphurea* 'Low nutrition'	-	-	GO:0004035 'alkaline phosphatase activity' [2]; GO:0005198 'structural molecule activity' [5]	KEGG:00730 'Thiamine metabolism' [2]; WP:3653 'Equilibrium Signalling Pathway of Differentiated Cells' [1]
1	*O. sulphurea* 'Native'	GO:0006067 'ethanol metabolic process' [2]; GO:0006090 'pyruvate metabolic process' [4]; GO:0006117 'acetaldehyde metabolic process' [2]; GO:0042335 'cuticle development' [6]; GO:0055114 'oxidation-reduction process' [9]	GO:0031430 'M band' [2]	GO:0008106 'alcohol dehydrogenase (NADP+) activity' [2]; GO:0008774 'acetaldehyde dehydrogenase (acetylating) activity' [2]; GO:0016491 'oxidoreductase activity' [9]	KEGG:00010 'Glycolysis / Gluconeogenesis' [5]; KEGG:00561 'Glycerolipid metabolism' [3]; KEGG:00620 'Pyruvate metabolism' [3]; WP:144 'Glycolysis and Gluconeogenesis' [3]
2	*O. sulphurea* 'Native'	GO:0007498 'mesoderm development' [7]; GO:0007591 'molting cycle, chitin-based cuticle' [6]; GO:0010927 'cellular component assembly involved in morphogenesis' [8]; GO:0017144 'drug metabolic process' [12]; GO:0032502 'developmental process' [37]; GO:0031032 'actomyosin structure organization' [6]; GO:0040003 'chitin-based cuticle development' [10]; GO:0042692 'muscle cell differentiation' [8]; GO:0048856 'anatomical structure development' [36]	GO:0005576 'extracellular region' [26]; GO:0015629 'actin cytoskeleton' [7]; GO:0031012 'extracellular matrix' [10]; GO:0033017 'sarcoplasmic reticulum membrane' [2]; GO:0099512 'supramolecular fiber' [10]	GO:0005198 'structural molecule activity' [16]; GO:0005214 'structural constituent of chitin-based cuticle' [9]; GO:0008307 'structural constituent of muscle' [3]; GO:0043167 'ion binding' [29]	WP:125 'Biogenic Amine Synthesis' [2]
2	*O. sulphurea* '2X alkaloids'	GO:0006067 'ethanol metabolic process' [2]; GO:0006575 'cellular modified amino acid metabolic process' [6]; GO:0006749 'glutathione metabolic process' [5]; GO:0006790 'sulfur compound metabolic process' [6]; GO:0009636 'response to toxic substance' [8]; GO:0055114 'oxidation-reduction process' [20]	GO:0005737 'cytoplasm' [31]; GO:0042175 'nuclear outer membrane-endoplasmic reticulum membrane network' [8]	GO:0003824 'catalytic activity' [34]; GO:0004364 'glutathione transferase activity' [6]; GO:0004497 'monooxygenase activity' [8]; GO:0008774 'acetaldehyde dehydrogenase (acetylating) activity' [2]; GO:0016491 'oxidoreductase activity' [20]; GO:0016765 'transferase activity, transferring alkyl or aryl (other than methyl) groups' [6]; GO:0020037 'heme binding' [8]; GO:0050660 'flavin adenine dinucleotide binding' [4]	KEGG:00053 'Ascorbate and aldarate metabolism' [4]; KEGG:00071 'Fatty acid degradation' [4]; KEGG:00480 'Glutathione metabolism' [6]; KEGG:00830 'Retinol metabolism' [5]; KEGG:00980 'Metabolism of xenobiotics by cytochrome P450' [11]; KEGG:00981 'Insect hormone biosynthesis' [3]
3	*T. terscheckii* 'Low nutrition'	GO:0006082 'organic acid metabolic process' [9]; GO:0006573 'valine metabolic process' [2]; GO:0055001 'muscle cell development' [4]; GO:0055114 'oxidation-reduction process' [16]	GO:0016529 'sarcoplasmic reticulum' [2]; GO:0005737 'cytoplasm' [23]; GO:0005739 'mitochondrion' [12]; GO:0043292 'contractile fiber' [4]; GO:0045261 'proton-transporting ATP synthase complex, catalytic core F(1)' [2]	GO:0016491 'oxidoreductase activity' [11]; GO:0036094 'small molecule binding' [13]; GO:0003824 'catalytic activity' [23]; GO:0000166 'nucleotide binding' [11]; GO:0043167 'ion binding' [17]; GO:0016614 'oxidoreductase activity, acting on CH-OH group of donors' [4]; GO:0050662 'coenzyme binding' [5]	KEGG:00020 'Citrate cycle (TCA cycle)' [3]; KEGG:00071 'Fatty acid degradation' [4]; KEGG:00280 'Valine, leucine and isoleucine degradation' [5]; KEGG:00410 'beta-Alanine metabolism' [3]; KEGG:01100 'Metabolic pathways' [13]; KEGG:01210 '2-Oxocarboxylic acid metabolism' [3]; KEGG:01212 'Fatty acid metabolism' [3]; WP:568 'Fatty Acid Biosynthesis' [2]
3	*T. terscheckii* 'Native'	GO:0004099 'chitin deacetylase activity'[2]	-	-	-
4	*T. terscheckii* 'Native'	GO:0040003 'chitin-based cuticle development' [3]	GO:0005576 'extracellular region' [5]; GO:0031012 'extracellular matrix' [3]	GO:0005214 'structural constituent of chitin-based cuticle' [3]	-
4	*T. terscheckii* '2X alkaloids'	GO:0055114 'oxidation-reduction process' [5]	GO:0042175 'nuclear outer membrane-endoplasmic reticulum membrane network' [4]	GO:0004497 'monooxygenase activity' [4]; GO:0016491 'oxidoreductase activity' [5]; GO:0020037 'heme binding' [5]	-

Four *a priori* planned INTRA-specific between treatments comparisons (1:exploratory *O. sulphurea*/Nutritional component, 2:*O. sulphurea*/Chemical component, 3:exploratory *T. terscheckii*/Nutritional component, 4:*T. terscheckii*/Chemical component)

*The order is as follows: GO term ID '*GO term name*' [number of DE genes annotated with that term]. **The order is as follows: KEGG pathway ID or WP pathway ID 'pathway name' [number of DE genes that mapped to that pathway].

Table S7. Summarized functional profiling based on gene ontology enrichment analysis and pathways overrepresentation (q<0.05) of *D. melanogaster* homolog genes for *D. koepferae* INTRA-specific analyses

D. koepferae		GO terms enrichment*			
	Pairwise comparison between cacti for each treatment	Biological process	Cellular component	Molecular function	Pathway overrepresentation**
1	*O. sulphurea* 'Low nutrition'	-	GO:0031012 'extracellular matrix' [4]	-	KEGG:00980 'Metabolism of xenobiotics by cytochrome P450' [2]
1	*T. terscheckii* 'Low nutrition'	GO:0006067 'ethanol metabolic process' [2]; GO:0006117 'acetaldehyde metabolic process' [2]; GO:0009636 'response to toxic substance' [5]; GO:0055114 'oxidation-reduction process' [12]	GO:0042175 'nuclear outer membrane-endoplasmic reticulum membrane network' [6]	GO:0003824 'catalytic activity' [21]; GO: 0004022 'alcohol dehydrogenase (NAD) activity' [2]; GO:0004497 'monooxygenase activity' [5]; GO:0008774 'acetaldehyde dehydrogenase (acetylating) activity' [2]; GO:0016491 'oxidoreductase activity' [12]; GO:0020037 'heme binding' [6]	KEGG:00010 'Glycolysis / Gluconeogenesis' [3]; KEGG:00053 'Ascorbate and aldarate metabolism' [3]; KEGG:00071 'Fatty acid degradation' [3]; KEGG:00830 'Retinol metabolism' [4]; KEGG:00980 'Metabolism of xenobiotics by cytochrome P450' [5]; KEGG:00981 'Insect hormone biosynthesis' [3]
2	*O. sulphurea* 'Native'	GO:0002181 'cytoplasmic translation' [21]; GO:0006082 'organic acid metabolic process' [13]; GO:0019401 'alditol biosynthetic process' [2]; GO:0042254 'ribosome biogenesis' [10]; GO:0043603 'cellular amide metabolic process' [23]; GO:0044282 'small molecule catabolic process' [8]; GO:0048878 'chemical homeostasis' [10]; GO:1901564 'organonitrogen compound metabolic process' [42]	GO:0005576 'extracellular region' [20]; GO:0005737 'cytoplasm' [51]; GO: 0005829 'cytosol' [34]; GO:0005840 'ribosome' [22]; GO:1990904 'ribonucleoprotein complex' [23]; GO: 0043228 'non-membrane-bounded organelle' [30]	GO:0019843 'rRNA binding' [6]; GO: 0003735 'structural constituent of ribosome' [21]; GO:0004613 'phosphoenolpyruvate carboxykinase (GTP) activity' [2]; GO:0005214 'structural constituent of chitin-based cuticle' [7]	KEGG:00010 'Glycolysis / Gluconeogenesis' [5]; KEGG:00500 'Starch and sucrose metabolism' [4]; KEGG:03010 'Ribosome' [21]; WP: 144 'Glycolysis and Gluconeogenesis' [3]
2	*T. terscheckii* 'Native'	-	GO:0005576 'extracellular region' [12]	-	-
3	*O. sulphurea* '2X alkaloids'	GO:0006090 'pyruvate metabolic process' [6]; GO:0006757 'ATP generation from ADP' [5]; GO:0007498 'mesoderm development' [8]; GO:0007525 'somatic muscle development' [7]; GO:0007591 'molting cycle, chitin-based cuticle' [7]; GO:0016052 'carbohydrate catabolic process' [6]; GO:0030036 'actin cytoskeleton organization' [15]; GO: 0040003 'chitin-based cuticle development' [11]; GO:0042026 'protein refolding' [4]; GO:0048646 'anatomical structure formation involved in morphogenesis' [16]; GO:0048856 'anatomical structure development' [47]; GO:0061061 'muscle structure development' [20]; GO:0072525 'pyridine-containing compound biosynthetic process' [5]	GO:0005576 'extracellular region' [24]; GO:0005829 'cytosol' [20]; GO:0015629 'actin cytoskeleton' [13]; GO:0016459 'myosin complex' [6]; GO:0022626 'cytosolic ribosome' [6]; GO:0031012 'extracellular matrix' [11]; GO:0043228 'non-membrane-bounded organelle' [32]; GO:0099512 'supramolecular fiber' [21]	GO:0003779 'actin binding' [9]; GO: 0005198 'structural molecule activity' [30]; GO:0005214 'structural constituent of chitin-based cuticle' [9]; GO:0005509 'calcium ion binding' [8]; GO:0008092 'cytoskeletal protein binding' [11]; GO: 0008307 'structural constituent of muscle' [6]	KEGG:00010 'Glycolysis / Gluconeogenesis' [6]; KEGG:01200 'Carbon metabolism' [7]; KEGG: 01230 'Biosynthesis of amino acids' [6]; WP:144 'Glycolysis and Gluconeogenesis' [5]
3	*T. terscheckii* '2X alkaloids'	GO:0007561 'imaginal disc eversion' [3]; GO:0009072 'aromatic amino acid family metabolic process' [4]	-	-	WP:125 'Biogenic Amine Synthesis' [2]

Three *a priori* planned INTRA-specific between cacti comparisons (1:exploratory 'Low nutrition', 2:'Native', 3:'2X alkaloids')

*The order is as follows: GO term ID '*GO term name*' [number of DE genes annotated with that term]. **The order is as follows: KEGG pathway ID or WP pathway ID 'pathway name' [number of DE genes that mapped to that pathway].

D. koepferae		GO terms enrichment*			
	Pairwise comparison between treatment for each cactus	Biological process	Cellular component	Molecular function	Pathway overrepresentation**
1	*O. sulphurea* 'Low nutrition'	-	-	-	-
1	*O. sulphurea* 'Native'	GO:0006082 'organic acid metabolic process' [24]; GO:0006094 'gluconeogenesis' [4]; GO:0006518 'peptide metabolic process' [17]; GO: 0008152 'metabolic process' [97]; GO: 0017144 'drug metabolic process' [22]; GO:0042593 'glucose homeostasis' [5]; GO:0042737 'drug catabolic process' [8]; GO:0055114 'oxidation-reduction process' [27]; GO: 1901564 'organonitrogen compound metabolic process' [58]	GO:0005737 'cytoplasm' [75]; GO: 0005739 'mitochondrion' [22]; GO: 0005829 'cytosol' [33]; GO:0005840 'ribosome' [14]	GO:0003824 'catalytic activity' [75]; GO: 0004613 'phosphoenolpyruvate carboxykinase (GTP) activity' [2]; GO: 0005198 'structural molecule activity' [17]; GO:0050662 'coenzyme binding' [10]; GO:0016614 'oxidoreductase activity, acting on CH-OH group of donors' [8]	KEGG:00010 'Glycolysis / Gluconeogenesis' [6]; KEGG:00500 'Starch and sucrose metabolism' [5]; KEGG:01100 'Metabolic pathways' [37]; KEGG:01200 'Carbon metabolism' [12]; WP:144 'Glycolysis and Gluconeogenesis' [6]
2	*O. sulphurea* 'Native'	GO:0006591 'ornithine metabolic process' [2]; GO:0008652 'cellular amino acid biosynthetic process' [2]; GO:0009064 'glutamine family amino acid metabolic process' [2]	GO:0030056 'hemidesmosome' [1]	GO:0004587 'ornithine-oxo-acid transaminase activity' [1]; GO:0004056 'argininosuccinate lyase activity' [1]	-
2	*O. sulphurea* '2X alkaloids'	-	-	GO:0020037 'heme binding '[3]	KEGG:00040 'Pentose and glucuronate interconversions '[2]; KEGG:00053 'Ascorbate and aldarate metabolism '[2]; KEGG:00830 'Retinol metabolism '[2]; KEGG:00860 'Porphyrin and chlorophyll metabolism '[2]
3	*T. terscheckii* 'Low nutrition'	-	-	-	-
3	*T. terscheckii* 'Native'	-	-	-	-
4	*T. terscheckii* 'Native'	-	-	-	-
4	*T. terscheckii* '2X alkaloids'	-	-	-	-

Four *a priori* planned INTRA-specific between treatments comparisons (1:exploratory *O. sulphurea*/Nutritional component, 2:*O. sulphurea*/Chemical component, 3:exploratory *T. terscheckii*/Nutritional component, 4:*T. terscheckii*/Chemical component)

*The order is as follows: GO term ID '*GO term name*' [number of DE genes annotated with that term]. **The order is as follows: KEGG pathway ID or WP pathway ID 'pathway name' [number of DE genes that mapped to that pathway].
