# Supplementary material for: Transcriptional responses are oriented towards different components of the rearing environment in two Drosophila sibling species
Source: BMC Genomics. 2022 Jul 16;23:515. doi: 10.1186/s12864-022-08745-9 (PMC9288027; doi:10.1186/s12864-022-08745-9)
Supplement: Supplementary file 3 — Additional file 3. Supporting figures: Fig.S1-S10. [file 12864_2022_8745_MOESM3_ESM.pdf]

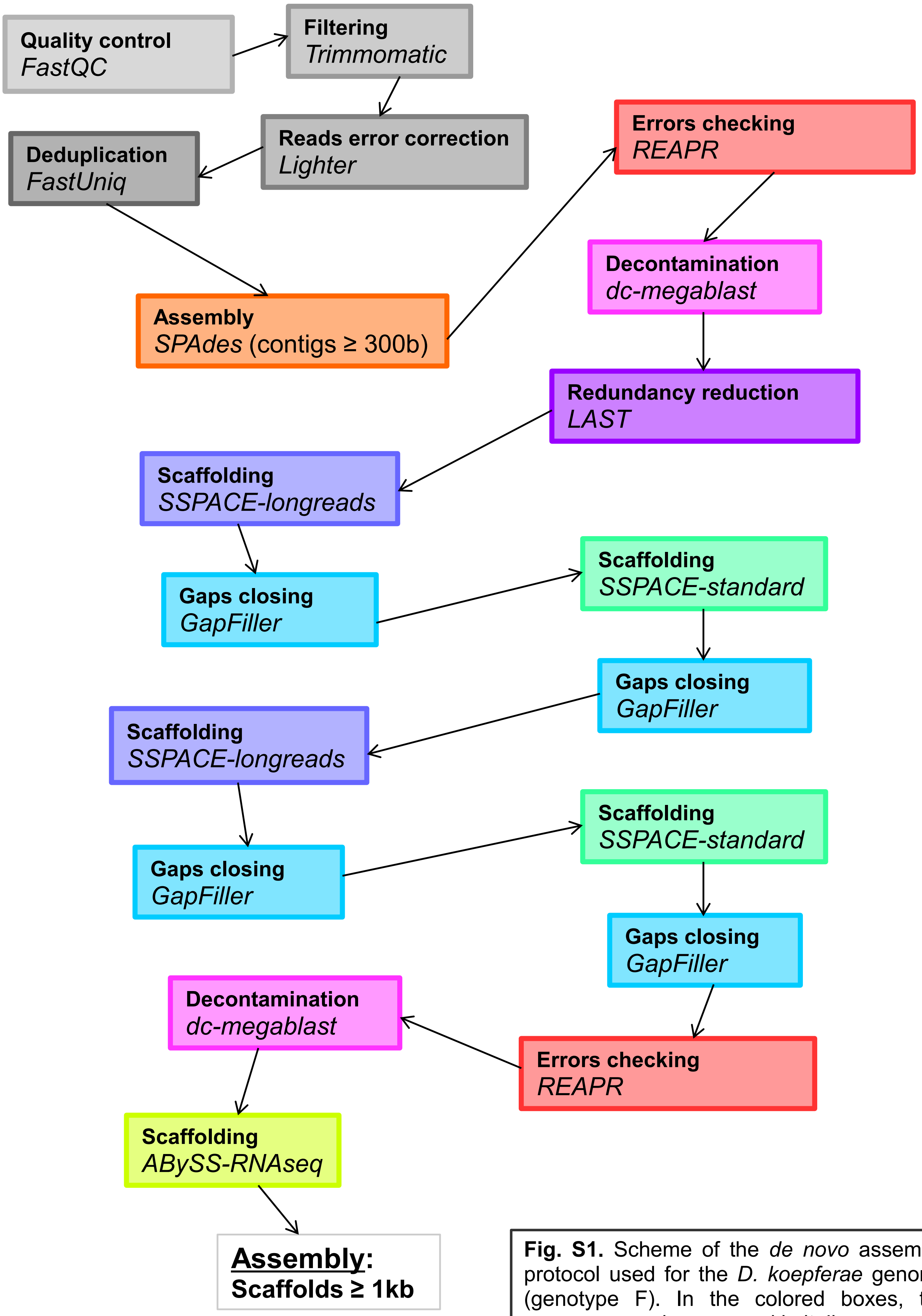

Fig. S1. Scheme of the *de novo* assembly protocol used for the *D. koepferae* genome (genotype F). In the colored boxes, the programs used are named in italics.

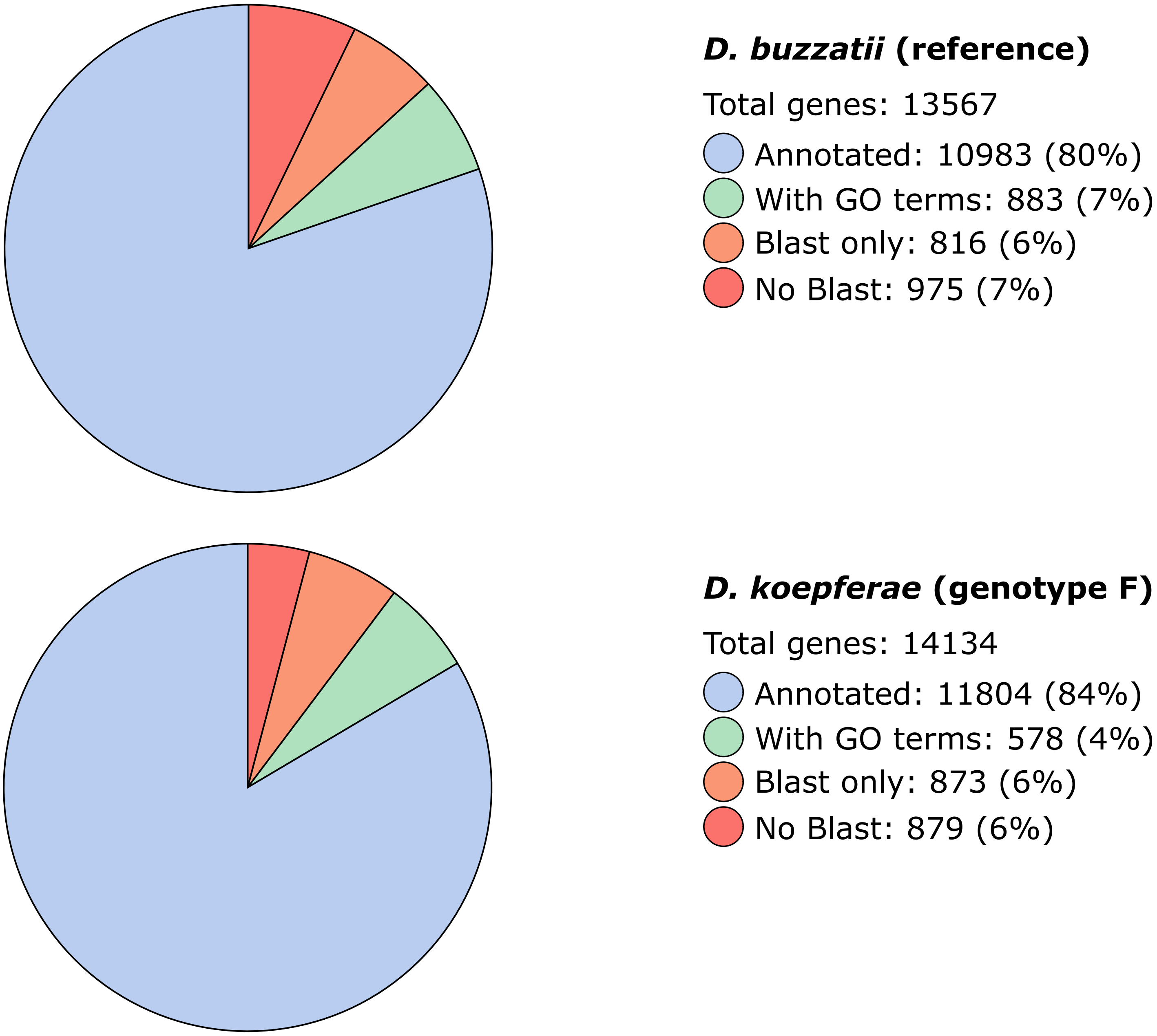

Fig. S2. Data distribution chart for the functional annotation pipeline with Blast2GO. "Annotated" depicts the amount of genes finally annotated at the end of the pipeline; "With GO terms" depicts the amount of genes with GO terms association that did not exceed the threshold of the default parameters to be tagged as annotated, "Blast Only" depicts the amount of sequences with a positive Blast that did not mapped to GO terms; "No Blast" depicts the amount of genes without a positive blast for the default parameters.

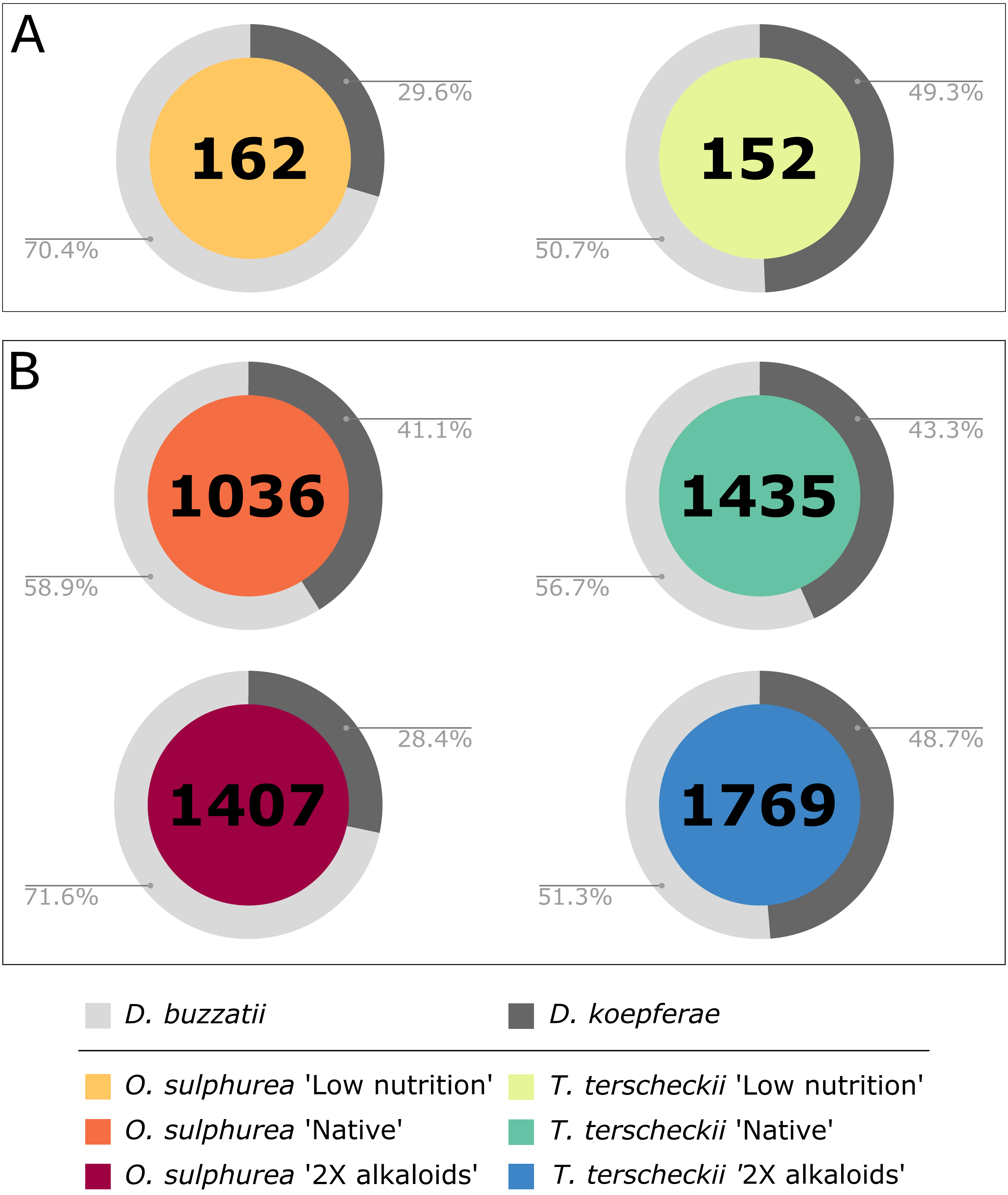

Fig. S3. Doughnuts plots representing the proportion of the DE genes comparatively overexpressed in each species during exploratory INTER-specific comparisons involving treatments with two (A) and three (B) biological replicates. The number in the centre corresponds to total DE genes, the portion and percentage corresponds to overexpressed genes in *D. buzzatii* or *D. koepferae*.

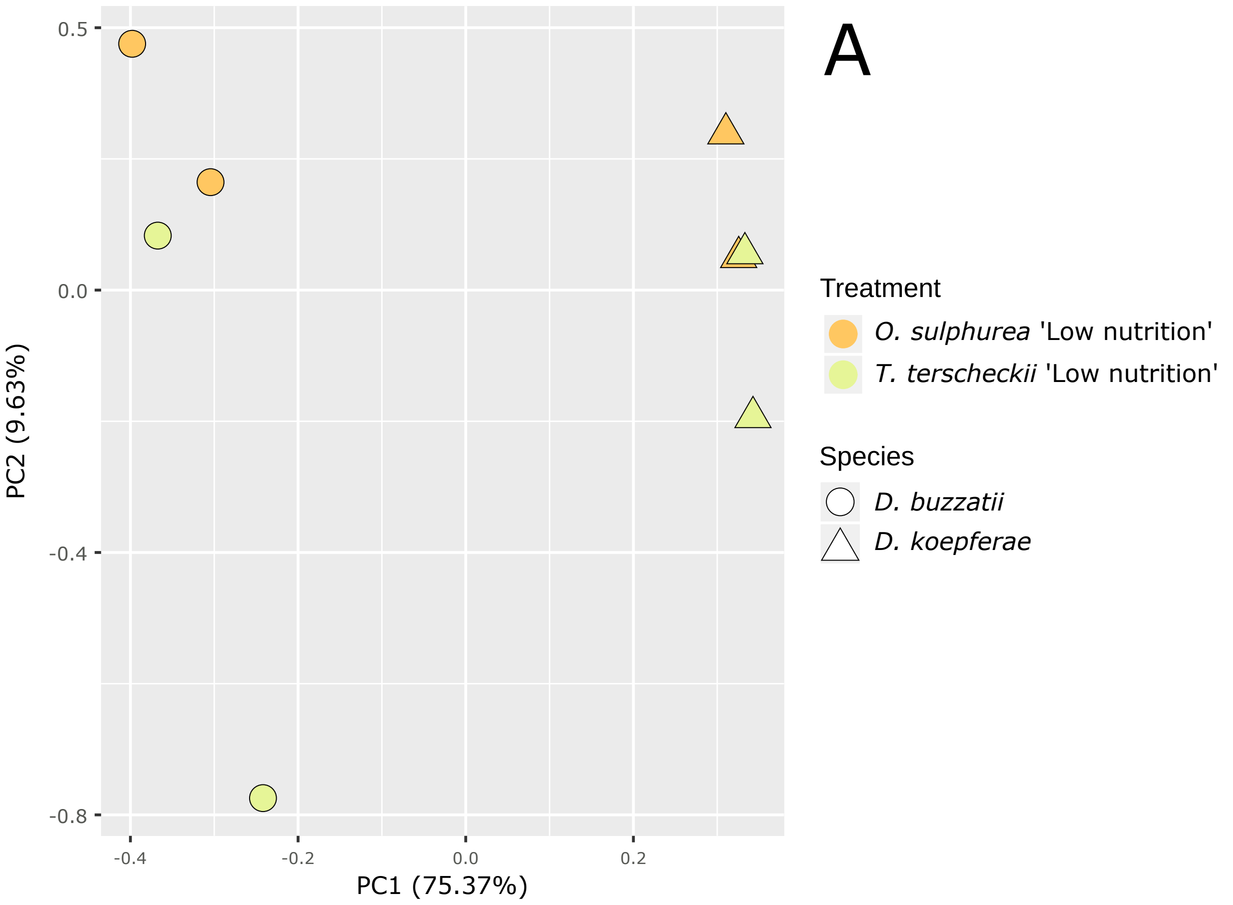

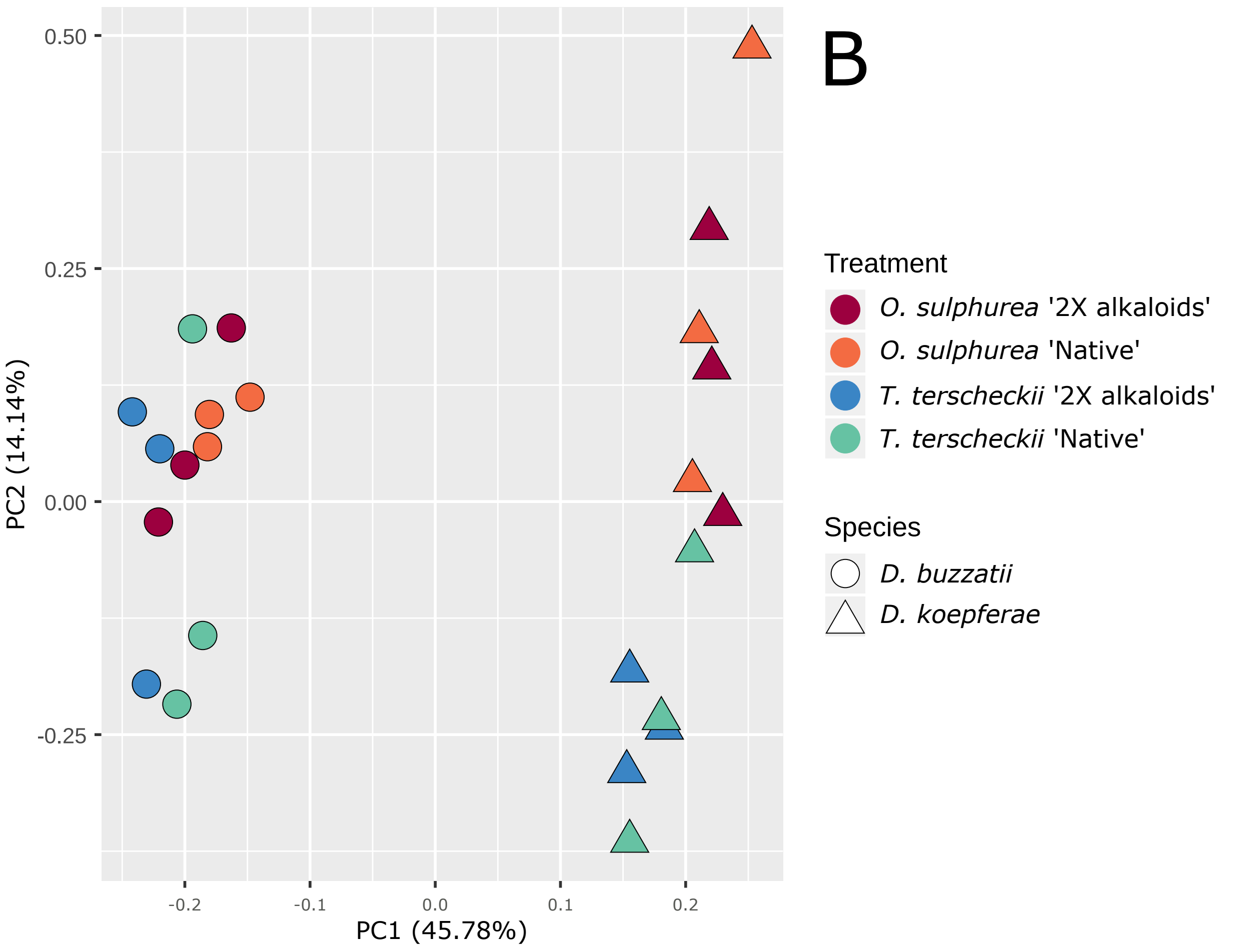

Fig. S4. Principal component analysis to visualize patterns of gene expression in exploratory INTER-specific analysis. **A.** Treatments without nutritional supplementation (three-replicate treatments). Variance explained by the axis: 85%. **B.** Nutritionally-supplemented treatments (two-replicate treatments). Variance explained by the axis: ~60%.

Fig. S5. Differentially expressed genes in exploratory INTER-specific analysis across treatments involved in the four phases of general xenobiotic transport and metabolism, corresponding mainly to six enzymatic groups: Solute carriers (SLC) transporters involved in Phase 0; Carboxylesterases (CE) and Oxidoreductases (OXR) in Phase I; Glutathione S-transferases (GST) and Glycosyltransferases (GT) in Phase II; and ATP-binding cassette (ABC) transporters in Phase III. Genes are identified by the respective *D. melanogaster* homolog. Row dendrograms are not shown. Heatmaps are split by species general overexpression for better visualization.

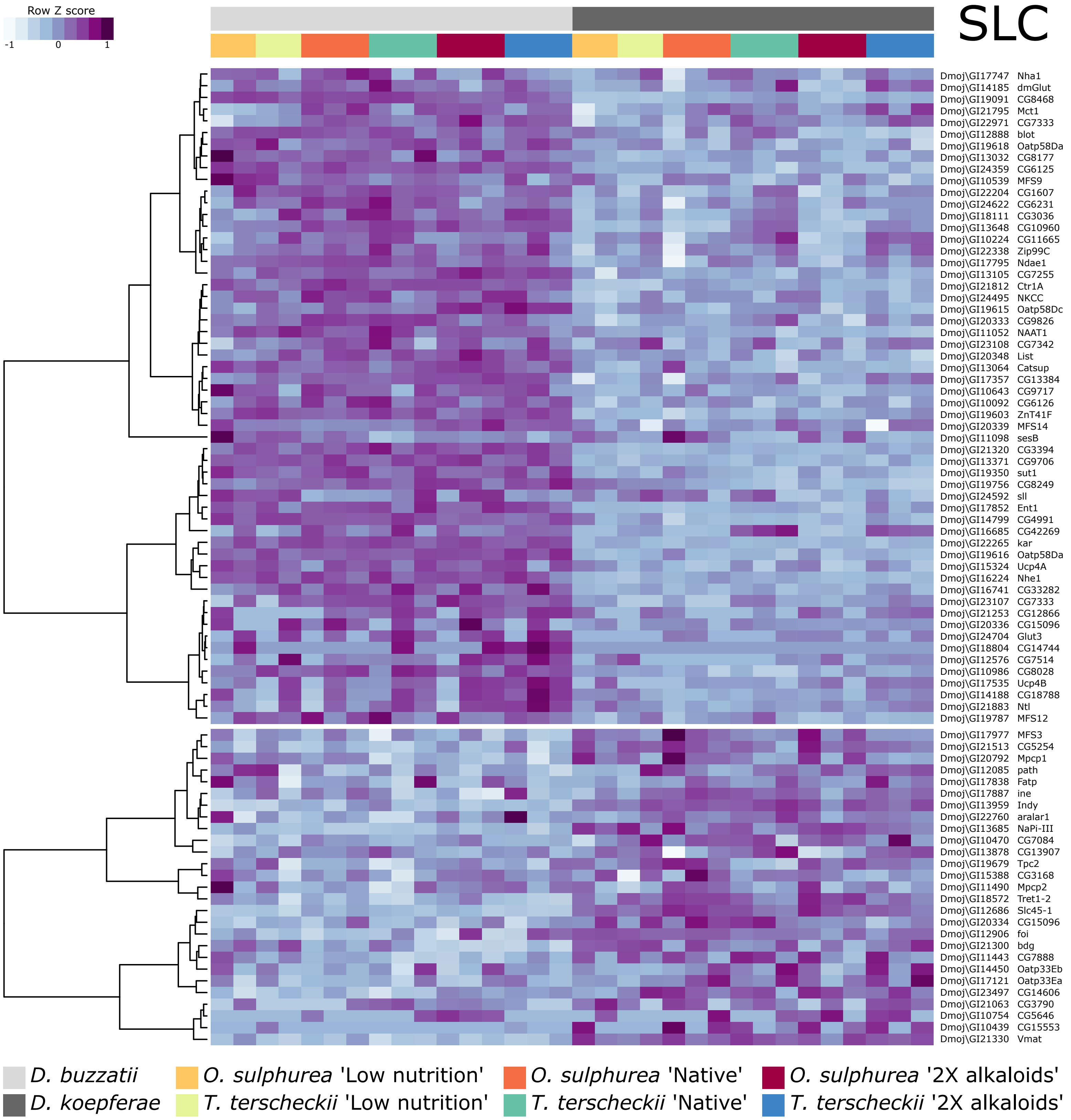

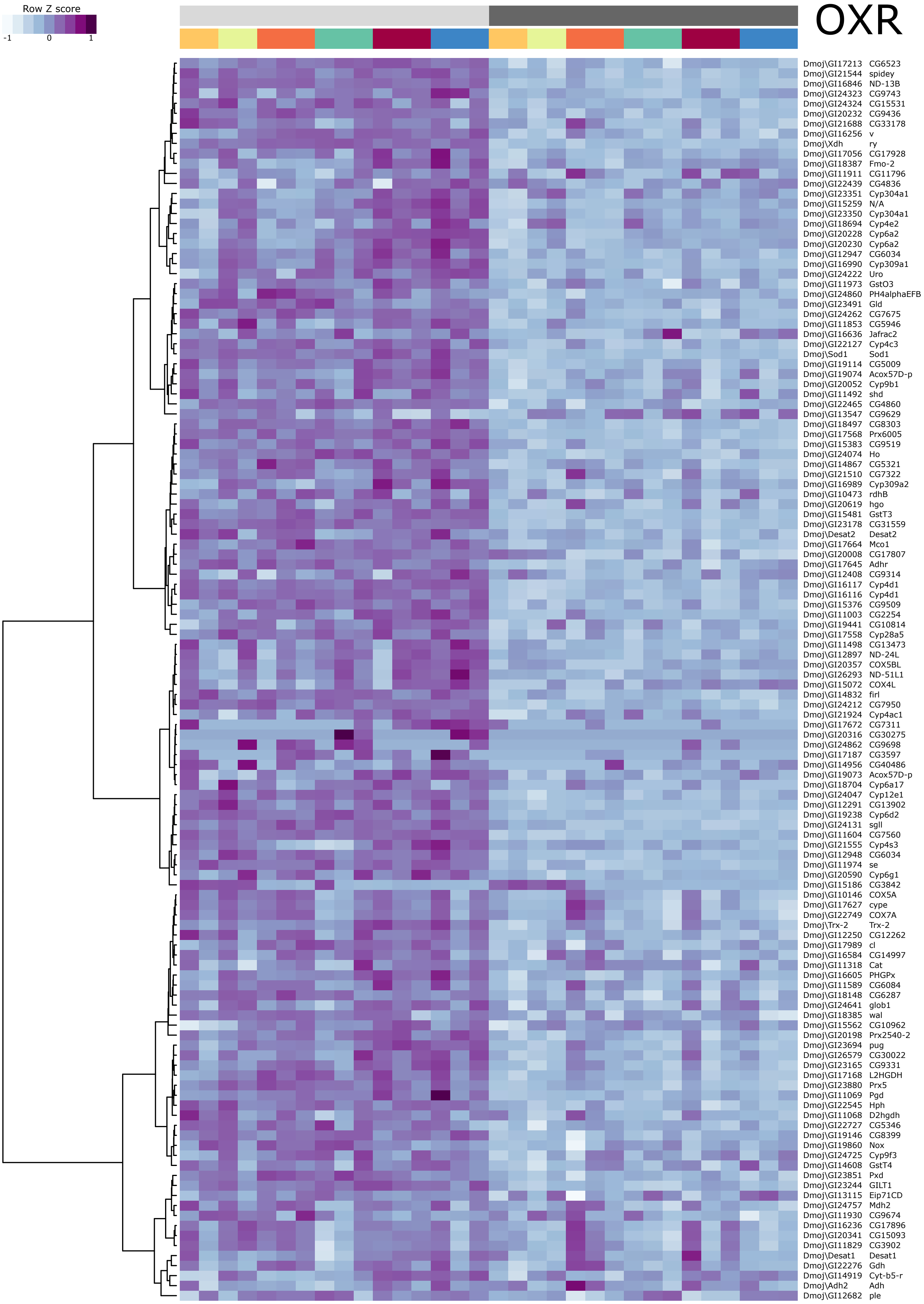

Row Z score
-1
0
1
OXR
Dmoj\GI17213 CG6523
Dmoj\GI21544 spidey
Dmoj\GI16846 ND-13B
Dmoj\GI24323 CG9743
Dmoj\GI24324 CG15531
Dmoj\GI20232 CG9436
Dmoj\GI21688 CG33178
Dmoj\GI16256 v
Dmoj\Xdh ry
Dmoj\GI17056 CG17928
Dmoj\GI18387 Fmo-2
Dmoj\GI11911 CG11796
Dmoj\GI22439 CG4836
Dmoj\GI23351 Cyp304a1
Dmoj\GI15259 N/A
Dmoj\GI23350 Cyp304a1
Dmoj\GI18694 Cyp4e2
Dmoj\GI20228 Cyp6a2
Dmoj\GI20230 Cyp6a2
Dmoj\GI12947 CG6034
Dmoj\GI16990 Cyp309a1
Dmoj\GI24222 Uro
Dmoj\GI11973 GstO3
Dmoj\GI24860 PH4alphaEFB
Dmoj\GI23491 Gld
Dmoj\GI24262 CG7675
Dmoj\GI11853 CG5946
Dmoj\GI16636 Jafrac2
Dmoj\GI22127 Cyp4c3
Dmoj\Sod1 Sod1
Dmoj\GI19114 CG5009
Dmoj\GI19074 Acox57D-p
Dmoj\GI20052 Cyp9b1
Dmoj\GI11492 shd
Dmoj\GI22465 CG4860
Dmoj\GI13547 CG9629
Dmoj\GI18497 CG8303
Dmoj\GI17568 Prx6005
Dmoj\GI15383 CG9519
Dmoj\GI24074 Ho
Dmoj\GI14867 CG5321
Dmoj\GI21510 CG7322
Dmoj\GI16989 Cyp309a2
Dmoj\GI10473 rdhB
Dmoj\GI20619 hgo
Dmoj\GI15481 GstT3
Dmoj\GI23178 CG31559
Dmoj\Desat2 Desat2
Dmoj\GI17664 Mco1
Dmoj\GI20008 CG17807
Dmoj\GI17645 Adhr
Dmoj\GI12408 CG9314
Dmoj\GI16117 Cyp4d1
Dmoj\GI16116 Cyp4d1
Dmoj\GI15376 CG9509
Dmoj\GI11003 CG2254
Dmoj\GI19441 CG10814
Dmoj\GI17558 Cyp28a5
Dmoj\GI11498 CG13473
Dmoj\GI12897 ND-24L
Dmoj\GI20357 COX5BL
Dmoj\GI26293 ND-51L1
Dmoj\GI15072 COX4L
Dmoj\GI14832 firl
Dmoj\GI24212 CG7950
Dmoj\GI21924 Cyp4ac1
Dmoj\GI17672 CG7311
Dmoj\GI20316 CG30275
Dmoj\GI24862 CG9698
Dmoj\GI17187 CG3597
Dmoj\GI14956 CG40486
Dmoj\GI19073 Acox57D-p
Dmoj\GI18704 Cyp6a17
Dmoj\GI24047 Cyp12e1
Dmoj\GI12291 CG13902
Dmoj\GI19238 Cyp6d2
Dmoj\GI24131 sgll
Dmoj\GI11604 CG7560
Dmoj\GI21555 Cyp4s3
Dmoj\GI12948 CG6034
Dmoj\GI11974 se
Dmoj\GI20590 Cyp6g1
Dmoj\GI15186 CG3842
Dmoj\GI10146 COX5A
Dmoj\GI17627 cype
Dmoj\GI22749 COX7A
Dmoj\Trx-2 Trx-2
Dmoj\GI12250 CG12262
Dmoj\GI17989 cl
Dmoj\GI16584 CG14997
Dmoj\GI11318 Cat
Dmoj\GI16605 PHGPx
Dmoj\GI11589 CG6084
Dmoj\GI18148 CG6287
Dmoj\GI24641 glob1
Dmoj\GI18385 wal
Dmoj\GI15562 CG10962
Dmoj\GI20198 Prx2540-2
Dmoj\GI23694 pug
Dmoj\GI26579 CG30022
Dmoj\GI23165 CG9331
Dmoj\GI17168 L2HGDH
Dmoj\GI23880 Prx5
Dmoj\GI11069 Pgd
Dmoj\GI22545 Hph
Dmoj\GI11068 D2hgdh
Dmoj\GI22727 CG5346
Dmoj\GI19146 CG8399
Dmoj\GI19860 Nox
Dmoj\GI24725 Cyp9f3
Dmoj\GI14608 GstT4
Dmoj\GI23851 Pxd
Dmoj\GI23244 GILT1
Dmoj\GI13115 Eip71CD
Dmoj\GI24757 Mdh2
Dmoj\GI11930 CG9674
Dmoj\GI16236 CG17896
Dmoj\GI20341 CG15093
Dmoj\GI11829 CG3902
Dmoj\Desat1 Desat1
Dmoj\GI22276 Gdh
Dmoj\GI14919 Cyt-b5-r
Dmoj\Adh2 Adh
Dmoj\GI12682 ple

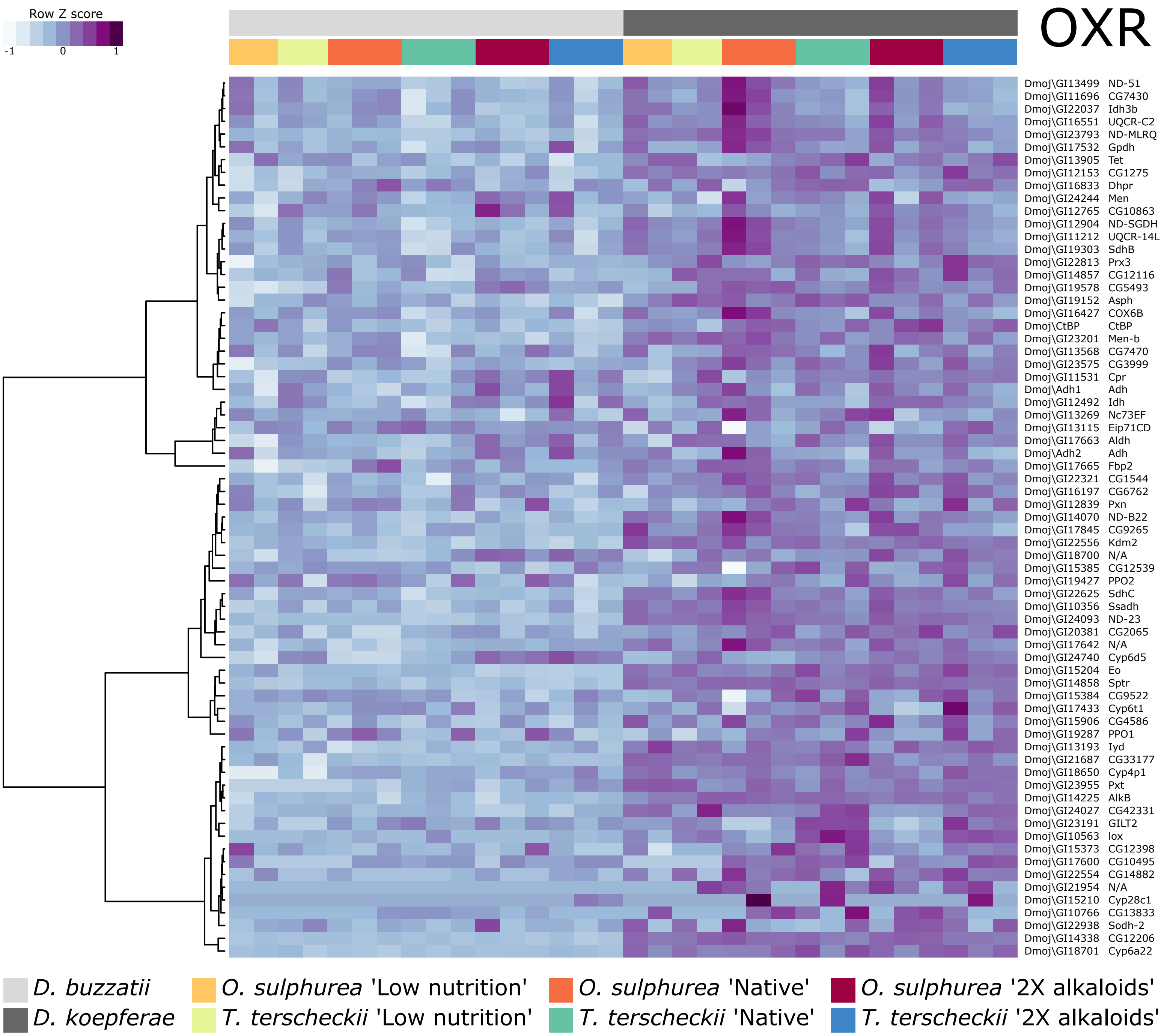

Row Z score
-1
0
1
OXR
Dmoj\GI13499 ND-51
Dmoj\GI11696 CG7430
Dmoj\GI22037 Idh3b
Dmoj\GI16551 UQCR-C2
Dmoj\GI23793 ND-MLRQ
Dmoj\GI17532 Gpdh
Dmoj\GI13905 Tet
Dmoj\GI12153 CG1275
Dmoj\GI16833 Dhpr
Dmoj\GI24244 Men
Dmoj\GI12765 CG10863
Dmoj\GI12904 ND-SGDH
Dmoj\GI11212 UQCR-14L
Dmoj\GI19303 SdhB
Dmoj\GI22813 Prx3
Dmoj\GI14857 CG12116
Dmoj\GI19578 CG5493
Dmoj\GI19152 Asph
Dmoj\GI16427 COX6B
Dmoj\CtBP CtBP
Dmoj\GI23201 Men-b
Dmoj\GI13568 CG7470
Dmoj\GI23575 CG3999
Dmoj\GI11531 Cpr
Dmoj\Adh1 Adh
Dmoj\GI12492 Idh
Dmoj\GI13269 Nc73EF
Dmoj\GI13115 Eip71CD
Dmoj\GI17663 Aldh
Dmoj\Adh2 Adh
Dmoj\GI17665 Fbp2
Dmoj\GI22321 CG1544
Dmoj\GI16197 CG6762
Dmoj\GI12839 Pxn
Dmoj\GI14070 ND-B22
Dmoj\GI17845 CG9265
Dmoj\GI22556 Kdm2
Dmoj\GI18700 N/A
Dmoj\GI15385 CG12539
Dmoj\GI19427 PPO2
Dmoj\GI22625 SdhC
Dmoj\GI10356 Ssadh
Dmoj\GI24093 ND-23
Dmoj\GI20381 CG2065
Dmoj\GI17642 N/A
Dmoj\GI24740 Cyp6d5
Dmoj\GI15204 Eo
Dmoj\GI14858 Sptr
Dmoj\GI15384 CG9522
Dmoj\GI17433 Cyp6t1
Dmoj\GI15906 CG4586
Dmoj\GI19287 PPO1
Dmoj\GI13193 Iyd
Dmoj\GI21687 CG33177
Dmoj\GI18650 Cyp4p1
Dmoj\GI23955 Pxt
Dmoj\GI14225 AlkB
Dmoj\GI24027 CG42331
Dmoj\GI23191 GILT2
Dmoj\GI10563 lox
Dmoj\GI15373 CG12398
Dmoj\GI17600 CG10495
Dmoj\GI22554 CG14882
Dmoj\GI21954 N/A
Dmoj\GI15210 Cyp28c1
Dmoj\GI10766 CG13833
Dmoj\GI22938 Sodh-2
Dmoj\GI14338 CG12206
Dmoj\GI18701 Cyp6a22
D. buzzatii
D. koepferae
O. sulphurea 'Low nutrition'
T. terscheckii 'Low nutrition'
O. sulphurea 'Native'
T. terscheckii 'Native'
O. sulphurea '2X alkaloids'
T. terscheckii '2X alkaloids'

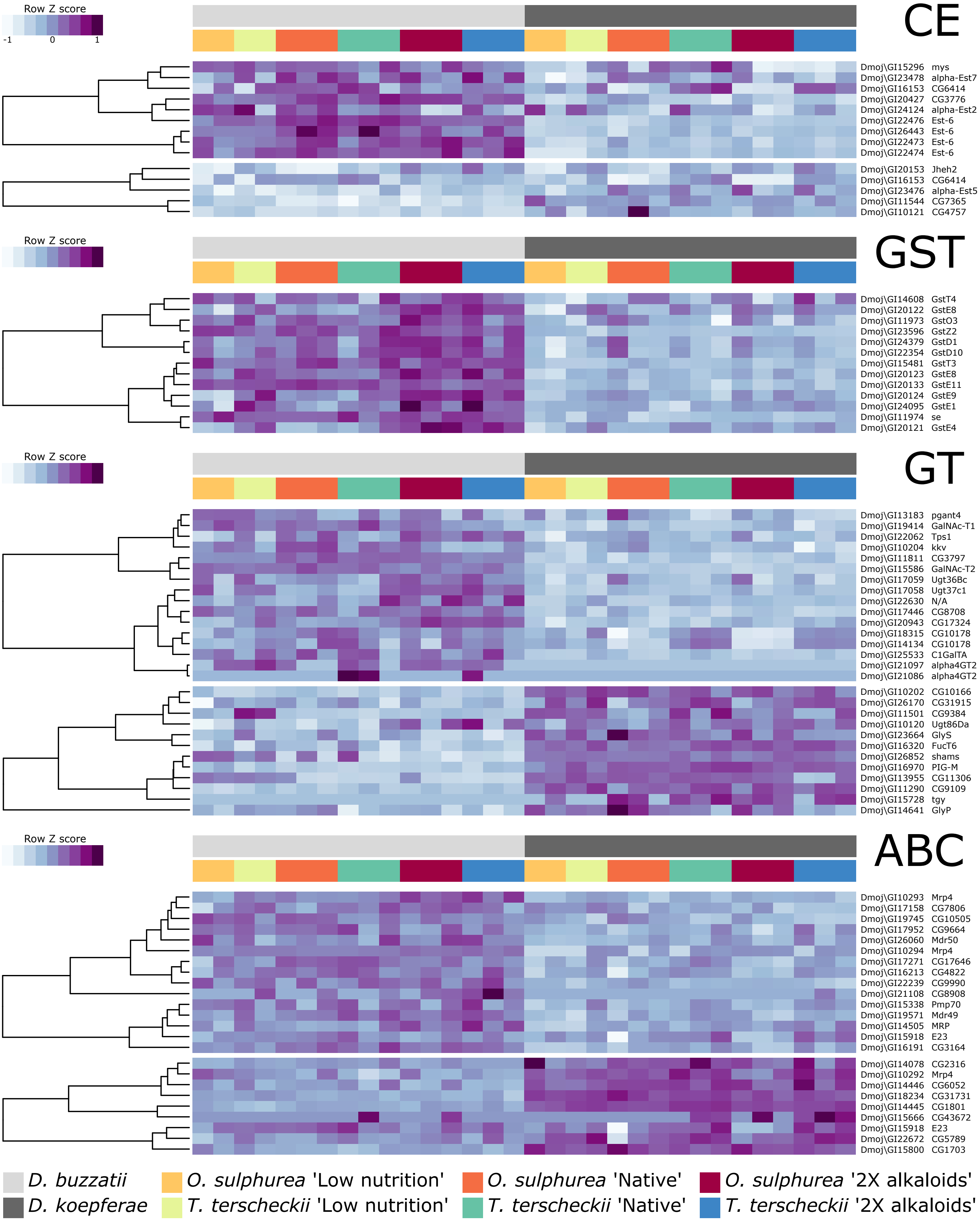
Row Z score
-1
0
1
CE
Dmoj\GI15296 mys
Dmoj\GI23478 alpha-Est7
Dmoj\GI16153 CG6414
Dmoj\GI20427 CG3776
Dmoj\GI24124 alpha-Est2
Dmoj\GI22476 Est-6
Dmoj\GI26443 Est-6
Dmoj\GI22473 Est-6
Dmoj\GI22474 Est-6
Dmoj\GI20153 Jheh2
Dmoj\GI16153 CG6414
Dmoj\GI23476 alpha-Est5
Dmoj\GI11544 CG7365
Dmoj\GI10121 CG4757
Row Z score
GST
Dmoj\GI14608 GstT4
Dmoj\GI20122 GstE8
Dmoj\GI11973 GstO3
Dmoj\GI23596 GstZ2
Dmoj\GI24379 GstD1
Dmoj\GI22354 GstD10
Dmoj\GI15481 GstT3
Dmoj\GI20123 GstE8
Dmoj\GI20133 GstE11
Dmoj\GI20124 GstE9
Dmoj\GI24095 GstE1
Dmoj\GI11974 se
Dmoj\GI20121 GstE4
Row Z score
GT
Dmoj\GI13183 pgant4
Dmoj\GI19414 GalNAc-T1
Dmoj\GI22062 Tps1
Dmoj\GI10204 kkv
Dmoj\GI11811 CG3797
Dmoj\GI15586 GalNAc-T2
Dmoj\GI17059 Ugt36Bc
Dmoj\GI17058 Ugt37c1
Dmoj\GI22630 N/A
Dmoj\GI17446 CG8708
Dmoj\GI20943 CG17324
Dmoj\GI18315 CG10178
Dmoj\GI14134 CG10178
Dmoj\GI25533 C1GalTA
Dmoj\GI21097 alpha4GT2
Dmoj\GI21086 alpha4GT2
Dmoj\GI10202 CG10166
Dmoj\GI26170 CG31915
Dmoj\GI11501 CG9384
Dmoj\GI10120 Ugt86Da
Dmoj\GI23664 GlyS
Dmoj\GI16320 FucT6
Dmoj\GI26852 shams
Dmoj\GI16970 PIG-M
Dmoj\GI13955 CG11306
Dmoj\GI11290 CG9109
Dmoj\GI15728 tgy
Dmoj\GI14641 GlyP
Row Z score
ABC
Dmoj\GI10293 Mrp4
Dmoj\GI17158 CG7806
Dmoj\GI19745 CG10505
Dmoj\GI17952 CG9664
Dmoj\GI26060 Mdr50
Dmoj\GI10294 Mrp4
Dmoj\GI17271 CG17646
Dmoj\GI16213 CG4822
Dmoj\GI22239 CG9990
Dmoj\GI21108 CG8908
Dmoj\GI15338 Pmp70
Dmoj\GI19571 Mdr49
Dmoj\GI14505 MRP
Dmoj\GI15918 E23
Dmoj\GI16191 CG3164
Dmoj\GI14078 CG2316
Dmoj\GI10292 Mrp4
Dmoj\GI14446 CG6052
Dmoj\GI18234 CG31731
Dmoj\GI14445 CG1801
Dmoj\GI15666 CG43672
Dmoj\GI15918 E23
Dmoj\GI22672 CG5789
Dmoj\GI15800 CG1703
D. buzzatii
D. koepferae
O. sulphurea 'Low nutrition'
T. terscheckii 'Low nutrition'
O. sulphurea 'Native'
T. terscheckii 'Native'
O. sulphurea '2X alkaloids'
T. terscheckii '2X alkaloids'

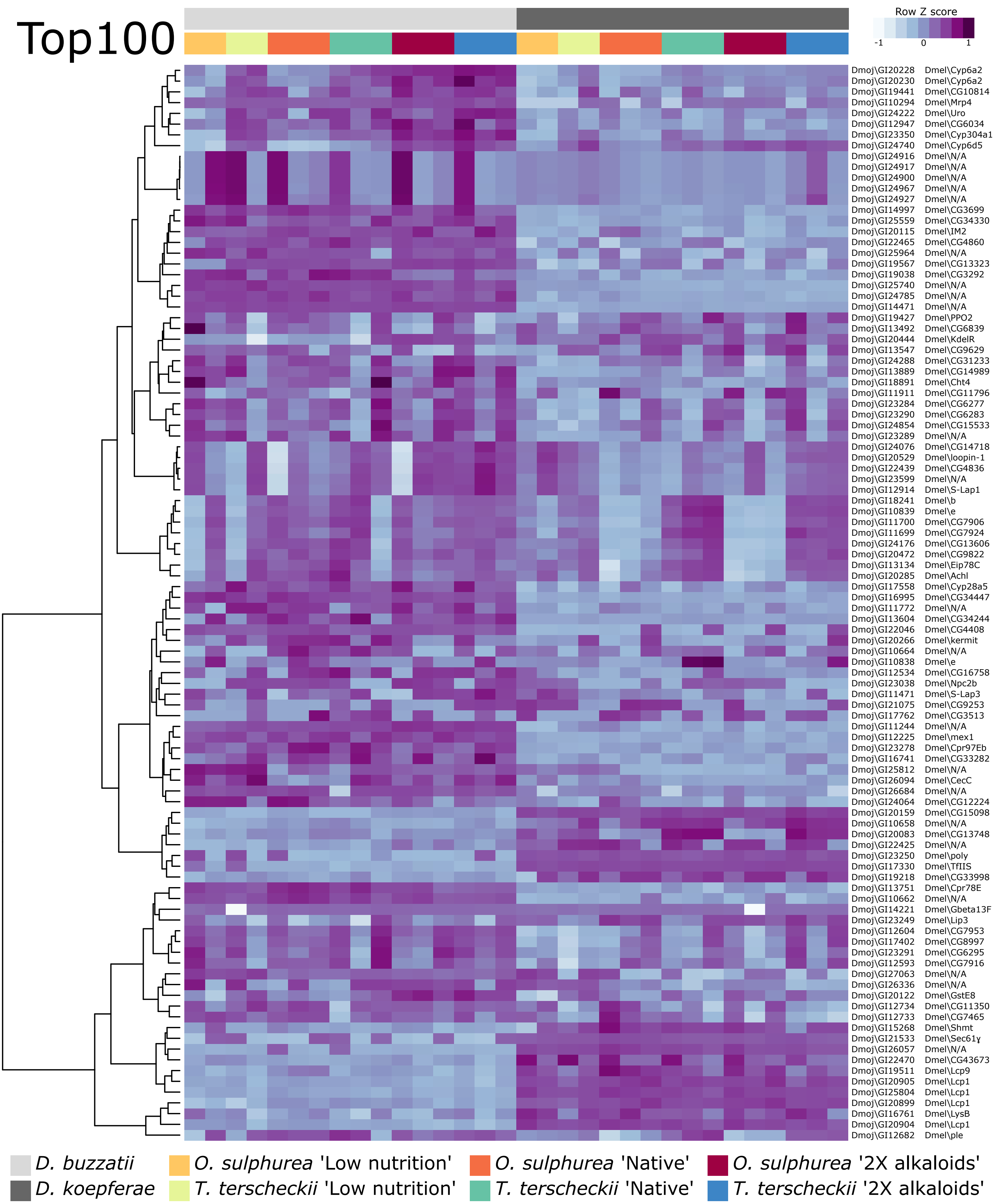

Fig. S6. Top 100 most variable differentially expressed genes in exploratory INTER-specific analyzes across treatments. Genes are identified by the respective *D. melanogaster* homolog. Row dendrograms are not shown.

Fig. S7. Main ontology terms taking into account all differentially expressed genes in INTRA-specific analyzes.

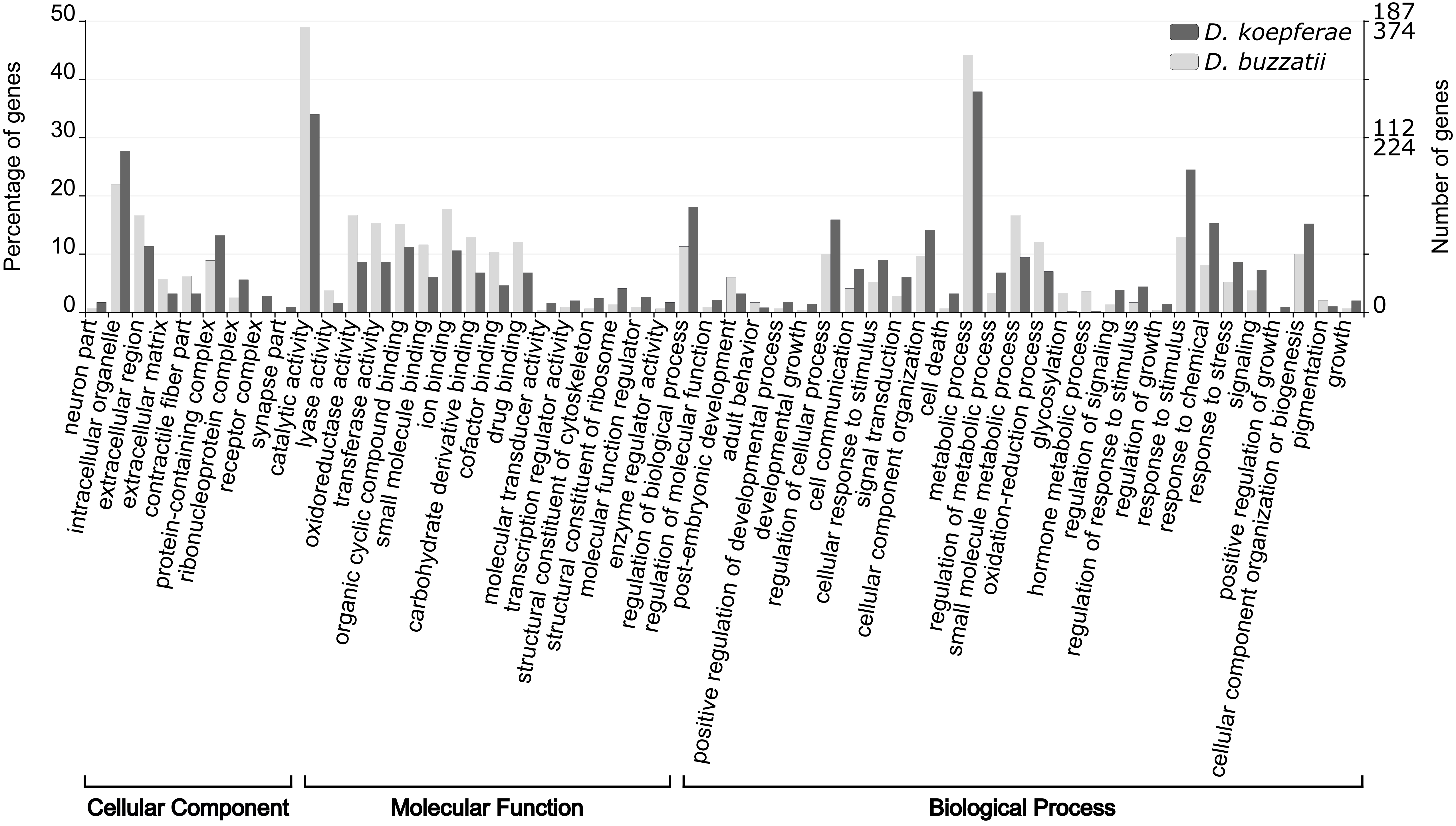

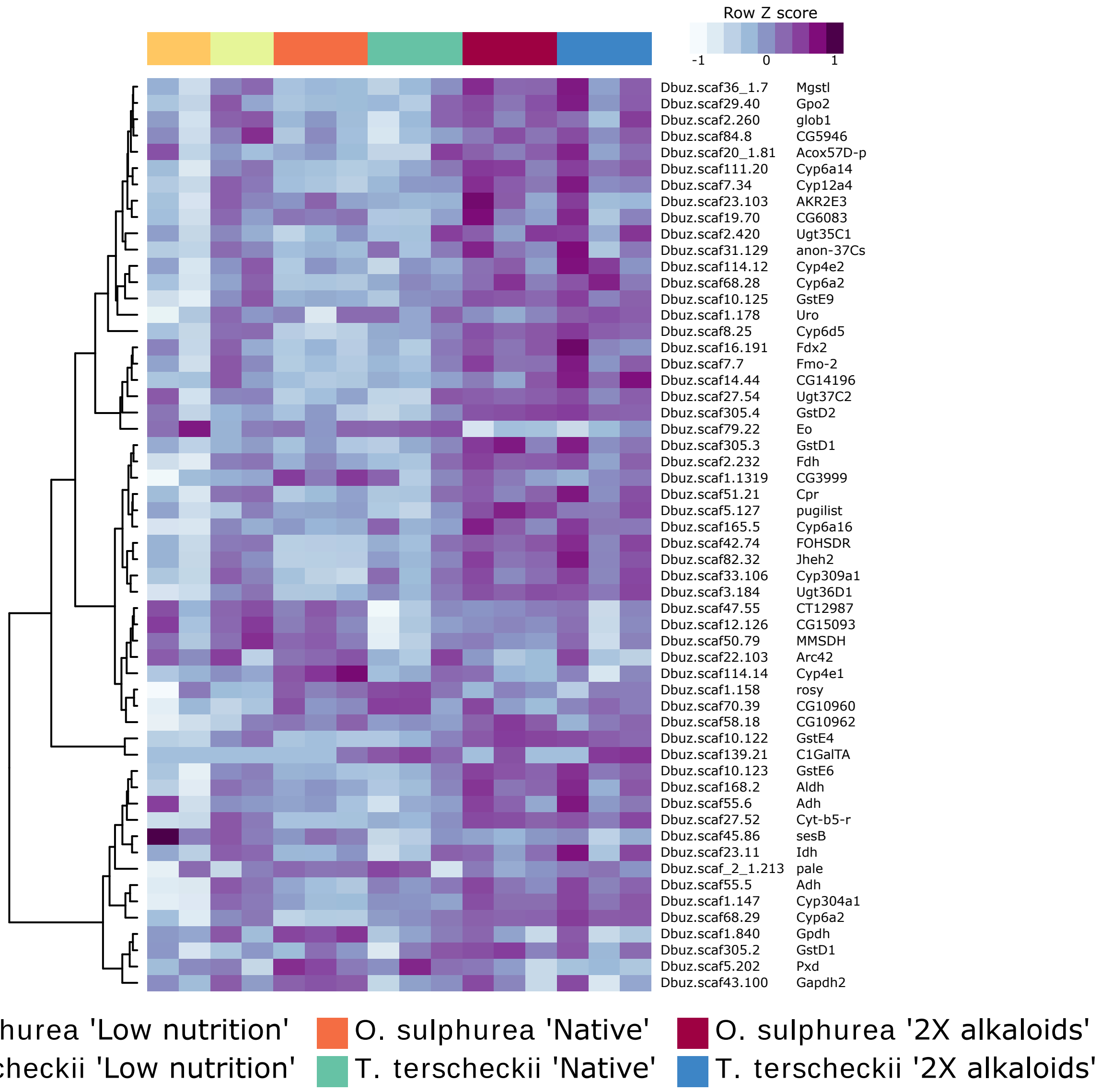

Fig. S8. D. buzzatii differentially expressed genes in INTRA-specific analyzes across treatments involved in the four phases of general xenobiotic transport and metabolism. Genes are identified by the respective D. melanogaster homolog. Row dendrograms are not shown.

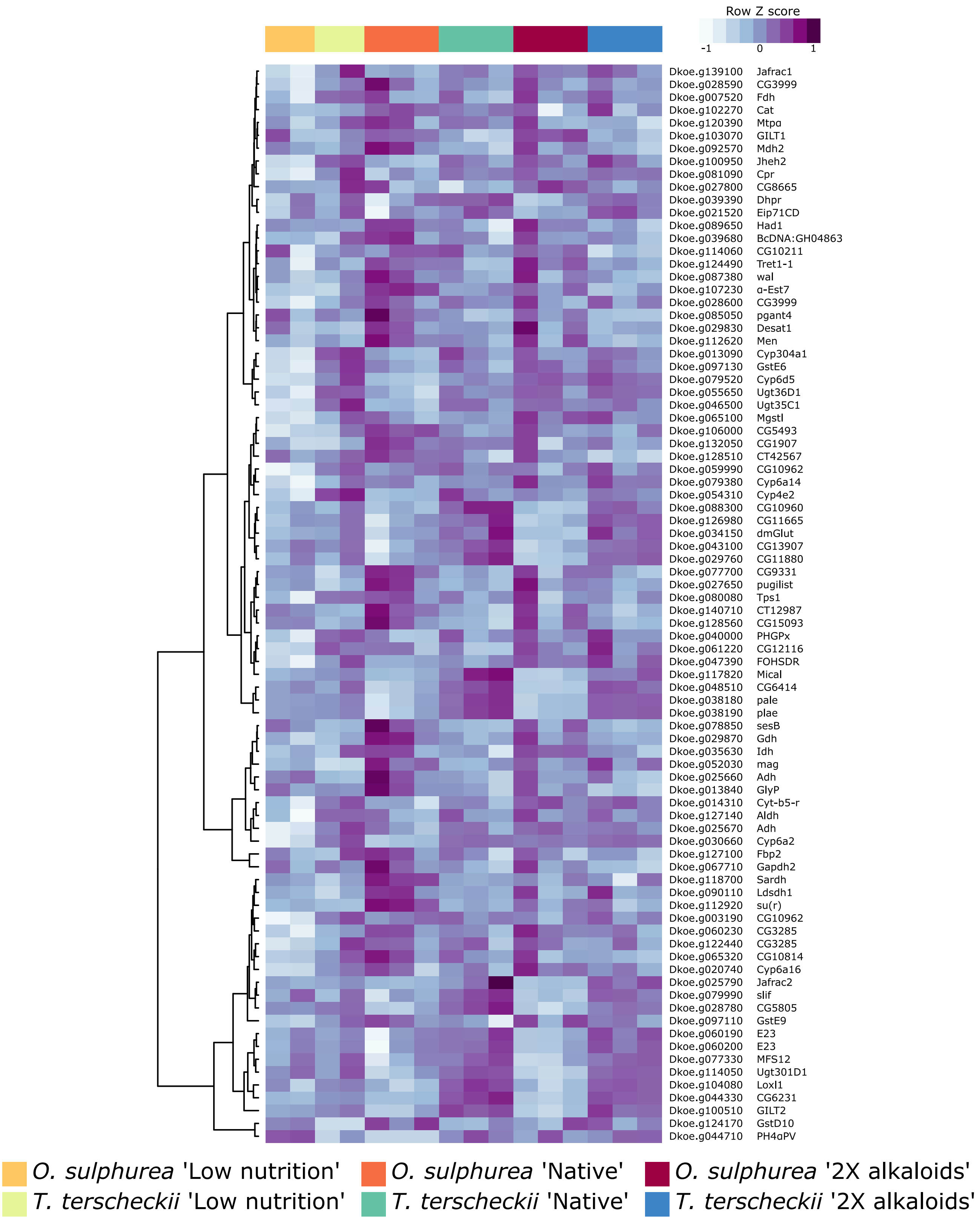

Fig. S9. *D. koepferae* differentially expressed genes in INTRA-specific analyzes across treatments involved in the four phases of general xenobiotic transport and metabolism. Genes are identified by the respective *D. melanogaster* homolog. Row dendrograms are not shown.

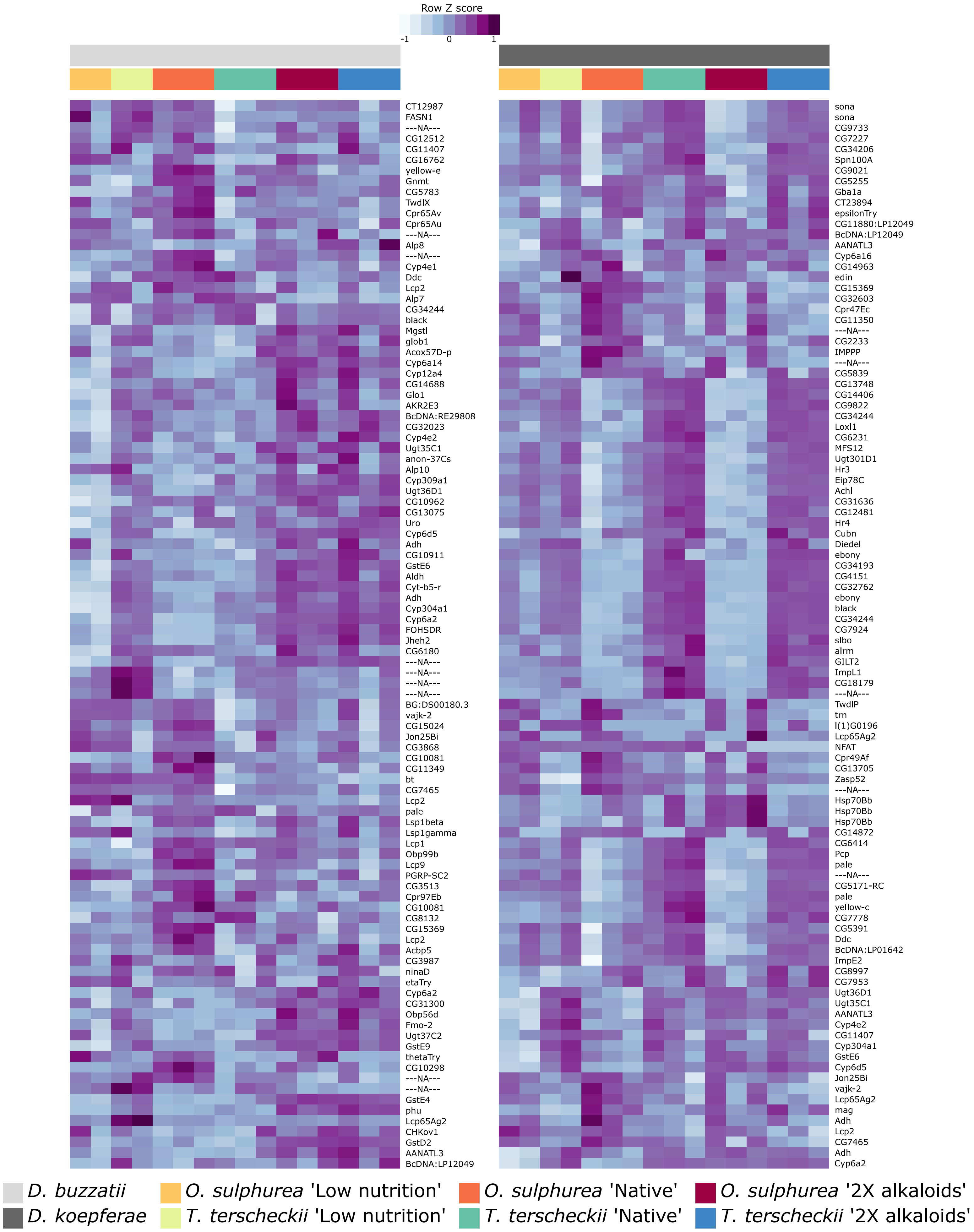

Fig. S10. Top 100 most variable differentially expressed genes in INTRA-specific analyzes in each species across treatments. Genes are identified by the respective *D. melanogaster* homolog. Row dendrograms are not shown.
